# Deciphering the Cryptic Genome: Genome-wide Analyses of the Rice Pathogen *Fusarium fujikuroi* Reveal Complex Regulation of Secondary Metabolism and Novel Metabolites

**DOI:** 10.1371/journal.ppat.1003475

**Published:** 2013-06-27

**Authors:** Philipp Wiemann, Christian M. K. Sieber, Katharina W. von Bargen, Lena Studt, Eva-Maria Niehaus, Jose J. Espino, Kathleen Huß, Caroline B. Michielse, Sabine Albermann, Dominik Wagner, Sonja V. Bergner, Lanelle R. Connolly, Andreas Fischer, Gunter Reuter, Karin Kleigrewe, Till Bald, Brenda D. Wingfield, Ron Ophir, Stanley Freeman, Michael Hippler, Kristina M. Smith, Daren W. Brown, Robert H. Proctor, Martin Münsterkötter, Michael Freitag, Hans-Ulrich Humpf, Ulrich Güldener, Bettina Tudzynski

**Affiliations:** 1 Institut für Biologie und Biotechnologie der Pflanzen, Molecular Biology and Biotechnology of Fungi, Westfälische Wilhelms-Universität Münster, Münster, Germany; 2 Institute of Bioinformatics and Systems Biology, Helmholtz Zentrum München, German Research Center for Environmental Health (GmbH), Neuherberg, Germany; 3 Institute for Food Chemistry, Westfälische Wilhelms-Universität Münster, Corrensstraße 45, Münster, Germany; 4 Institut für Biologie und Biotechnologie der Pflanzen, Plant Biochemistry and Biotechnology, Westfälische Wilhelms-Universität Münster, Münster, Germany; 5 Department of Biochemistry and Biophysics, Center for Genome Research and Biocomputing, Oregon State University, Corvallis, Oregon, United States of America; 6 Institut of Genetics/Developmental Genetics, Martin-Luther-Universität Halle-Wittenberg, Halle, Germany; 7 Department of Genetics, University of Pretoria, Hatfield, Pretoria, South Africa; 8 Institute of Plant Sciences, Genomics, Agricultural Research Organization (ARO), The Volcani Center, Bet-Dagan, Israel; 9 Department of Plant Pathology, Agricultural Research Organization (ARO), The Volcani Center, Bet-Dagan, Israel; 10 National Center for Agricultural Utilization Research, United States Department of Agriculture, Peoria, Illinois, United States of America; Carnegie Mellon University, United States of America

## Abstract

The fungus *Fusarium fujikuroi* causes “bakanae” disease of rice due to its ability to produce gibberellins (GAs), but it is also known for producing harmful mycotoxins. However, the genetic capacity for the whole arsenal of natural compounds and their role in the fungus' interaction with rice remained unknown. Here, we present a high-quality genome sequence of *F. fujikuroi* that was assembled into 12 scaffolds corresponding to the 12 chromosomes described for the fungus. We used the genome sequence along with ChIP-seq, transcriptome, proteome, and HPLC-FTMS-based metabolome analyses to identify the potential secondary metabolite biosynthetic gene clusters and to examine their regulation in response to nitrogen availability and plant signals. The results indicate that expression of most but not all gene clusters correlate with proteome and ChIP-seq data. Comparison of the *F. fujikuroi* genome to those of six other fusaria revealed that only a small number of gene clusters are conserved among these species, thus providing new insights into the divergence of secondary metabolism in the genus *Fusarium*. Noteworthy, GA biosynthetic genes are present in some related species, but GA biosynthesis is limited to *F. fujikuroi*, suggesting that this provides a selective advantage during infection of the preferred host plant rice. Among the genome sequences analyzed, one cluster that includes a polyketide synthase gene (*PKS19*) and another that includes a non-ribosomal peptide synthetase gene (*NRPS31*) are unique to *F. fujikuroi*. The metabolites derived from these clusters were identified by HPLC-FTMS-based analyses of engineered *F. fujikuroi* strains overexpressing cluster genes. *In planta* expression studies suggest a specific role for the *PKS19*-derived product during rice infection. Thus, our results indicate that combined comparative genomics and genome-wide experimental analyses identified novel genes and secondary metabolites that contribute to the evolutionary success of *F. fujikuroi* as a rice pathogen.

## Author Summary

Fungi produce numerous “secondary metabolites” (SMs) that are not essential for life but can provide an advantage under natural conditions, e.g. in fungal-host interactions. Here, we conducted the most comprehensive analysis to date of secondary metabolism in fungi using *Fusarium fujikuroi*. This fungus causes “bakanae” disease of rice and is best known for its ability to produce gibberellins (GAs). We show that GA production is limited to *F. fujikuroi* and provides a selective advantage during infection of the preferred host plant rice. Generation and analysis of a high-quality *de novo F. fujikuroi* genome sequence combined with comparisons to six other *Fusarium* genomes revealed the presence of 45 mostly unknown SM gene clusters. We provide a broad spectrum of experimental data including epigenetic, transcriptional, proteomic and chemical product analyses under different nitrogen and pH conditions. Two of the SM clusters (PKS19 and NRPS31) are not present in any other sequenced fungal genome. *In planta* expression studies revealed that the otherwise silent PKS19 cluster is induced on rice, but not on maize, suggesting a specific role for the *PKS19*-derived product during rice infection. Together, our results demonstrate the tremendous potential of a single fungal species to produce a diversity of SMs that likely contributes to adaptation to environmental changes.

## Introduction

**Figure 1:**
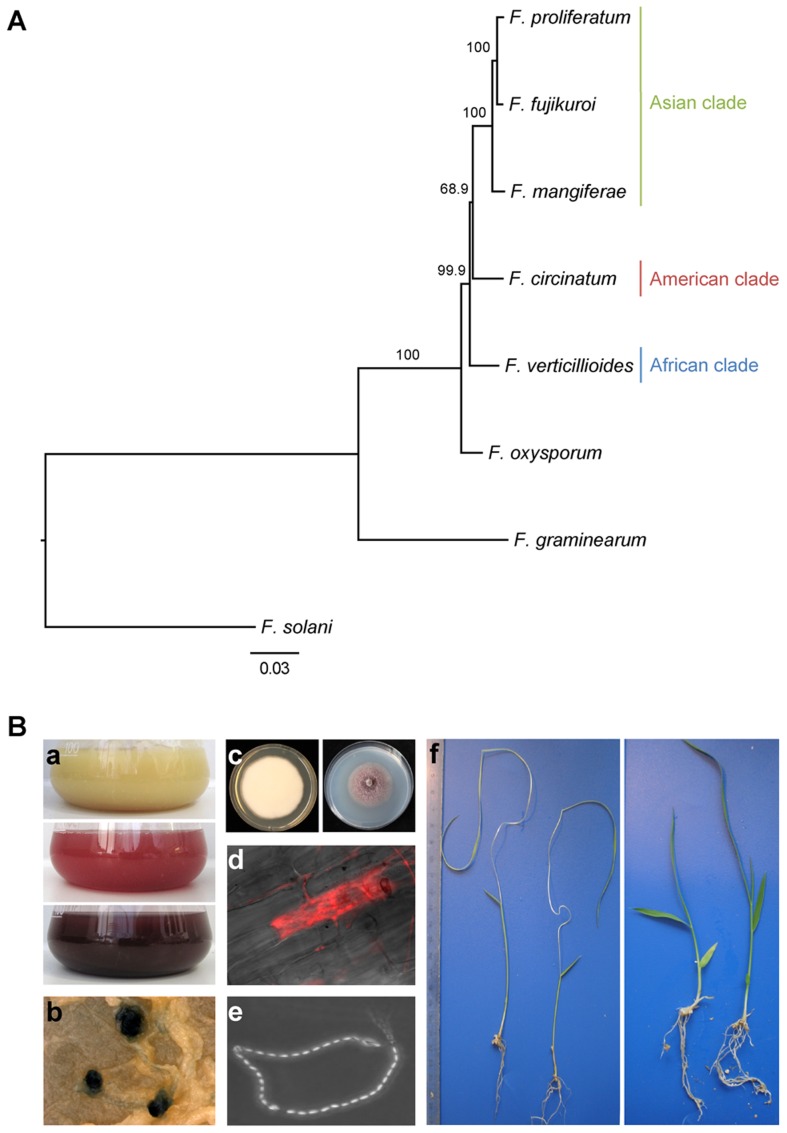
Phylogenetic and phenotypic characteristics of *F. fujikuroi*. **A**: Maximum likelihood tree showing phylogenetic relationships of *F. fujikuroi* and other species representing the Asian, African and American clades of the *Gibberella fujikuroi* complex (GFC), as well as *F. oxysporum*, *F. graminearum* and *F. solani*. The midpoint rooted tree is based on concatenated nucleotide sequences of 28 genes involved in primary metabolism that are highly homologous in different fusaria. Branches show bootstrap values (%), scale bar indicates amino acid substitutions per site. **B**: Phenotypic characteristics: a) Variation in pigmentation of the wild-type *F. fujikuroi* grown in a liquid medium containing (from top to bottom) 60 mM glutamine (fusarins), 6 mM NaNO_3_ (fusarubins) and 6 mM glutamine (bikaverin); b) Perithecia resulting from a sexual cross of two isolates of *F. fujikuroi* with opposite mating types. Fusarubins account for the dark color of perithecia [15]; c) *F. fujikuroi* grown on complete medium and regeneration medium; d) fluorescent microscopy image of the DsRed-labeled *F. fujikuroi* wild type penetrating rice root cells during infection; e) Microscopic image of microconidial chain on KCl agar medium; f) characteristic symptoms of bakanae disease due to wild-type-infected rice seedlings (left) compared to the GA-deficient mutant (right).

The genus *Fusarium* is one of the most important groups of phytopathogenic fungi. They infect a broad spectrum of crops worldwide and are responsible for huge economic losses due to yield reductions and mycotoxin contamination. The *Gibberella fujikuroi* species complex (GFC) constitutes a monophyletic but diverse subgroup of over 50 *Fusarium* species with similar morphological features. The complex is divided into the African, American and Asian clades, according to DNA-based phylogenetic analyses [1]–[3] (Figure 1A).

The species *Fusarium fujikuroi* Nirenberg (teleomorph *Gibberella fujikuroi* (Sawada) Wollenweber) was first described more than 100 years ago as the causative agent of the “bakanae” (“foolish seedling”) disease of rice in Japan [2]–[4]. The most characteristic symptom of this disease is excessively elongated seedlings with chlorotic stems and leaves (Figure 1B). In addition, affected plants are infertile and therefore do not produce edible grains. The disease symptoms result from the ability of *F. fujikuroi* to produce and secrete gibberellic acids (GAs), a family of plant hormones [5], [6]. Today, the fungus is used worldwide for the commercial production of GAs, which are applied extensively in horticulture to regulate plant growth and development [7]. Although GAs control growth in higher plants, they are considered as secondary metabolites (SMs) in *Fusarium* because they are not essential for fungal growth and development but instead are thought to contribute to the virulence of the pathogen.

Many fusaria, including multiple species in the GFC, are noted for their production of other SMs, particularly pigments and mycotoxins. In fungi, genes responsible for the synthesis of a SM are typically located adjacent to one another in a gene cluster. Such clusters typically include a gene encoding a polyketide synthase (PKS), non-ribosomal peptide synthetase (NRPS) or terpene cyclase (TC) that is responsible for conversion of primary metabolite(s) to a molecule that serves as a precursor for synthesis of a biologically active SM or family of structurally related SMs (e.g. GAs). SM biosynthetic gene clusters can also include genes that encode: 1) tailoring enzymes that catalyze modification of the precursor molecule or subsequent intermediates in a SM biosynthetic pathway; 2) proteins that transport SMs or intermediates across cellular membranes; and 3) pathway-specific transcription factors that typically induce expression of all the genes in a cluster.

The best studied SMs in *F. fujikuroi* are the diterpenoid GAs. Two major milestones in research on GA biosynthesis in this fungus were the identification of the seven-gene GA biosynthetic gene cluster in *F. fujikuroi* [8], [9] and the discovery that these genes are regulated by the global nitrogen regulator AreA which had not previously been linked to secondary metabolism [10], [11]. Subsequent work revealed a correlation between nitrogen availability and production of other SMs by *F. fujikuroi* [12], including carotenoids [13], the red PKS-derived pigments bikaverin and fusarubins [14], [15], and the mycotoxins fusarins [16]–[18] (Figure 1B).

The availability of genome sequences has significantly impacted examination of secondary metabolism in fungi [19]–[30]. To date, publicly available genome sequences of five *Fusarium* species (*F. graminearum*, *F. oxysporum*, *F. pseudograminearum*, *F. solani* and *F. verticillioides*) have aided *in silico* examination of secondary metabolism in *Fusarium*. The sequences have facilitated the establishment of a preliminary catalogue of PKS and NRPS genes in *Fusarium* [31], [32], examinations of their phylogenetic relationships [33], [34], and bioinformatic identification of novel SM biosynthetic gene clusters [22]. However, none of the functionally characterized gene clusters have led to the identification of any new secondary metabolite that has not been found to be produced by *Fusarium* spp. before.

Most species in the GFC produce multiple SMs [2], but only a fraction of them have been linked to specific biosynthetic genes. Prior to this study, a complete genome sequence has been available for only one member of the GFC, the African clade species *F. verticillioides*, which causes ear and stalk rot of maize [22]. The genomes of the American clade species *F. circinatum*, the cause of pitch canker of pine [35], and the Asian clade species *F. mangiferae*, that causes mango malformation [36], [37] (recently sequenced by S. Freeman and coworkers), are not yet publicly available but were used in the current study for comparison of secondary metabolism both *in silico* and in laboratory experiments.

The objective of the current study is the comprehensive analysis of secondary metabolism in *F. fujikuroi*, the cause of “bakanae” disease of rice. We present a draft genome sequence and *de novo* assembly of exceptional quality for *F. fujikuroi*, a member of the Asian clade of the GFC. We assembled 12 scaffolds that correspond to the 12 previously identified GFC chromosomes [38], [39]. In addition to the well-known GA gene cluster, we describe novel genes coding for key SM biosynthetic enzymes, such as PKSs, NRPSs, TCs, dimethylallyl tryptophane synthases (DMATSs), and cytochrome P450 monooxygenases (P450s) and thereby decoded the complete potential of this important species to produce SMs. Our analyses revealed differences and similarities between species of different GFC clades and the more distantly related *F. oxysporum* (Figure 1A). By applying a combination of microarrays, ChIP-seq, proteomics, and HPLC-FTMS analyses, we demonstrate that nitrogen availability has an enormous impact on secondary metabolism by affecting gene expression, histone modification patterns, protein composition, and SM product levels. Two of the gene clusters (PKS19 and NRPS31) are not present in any other sequenced fungal genome and thus unique to *F. fujikuroi*. The forced expression of these unique clusters by genetic engineering led to structural characterization of corresponding metabolites by HPLC-FTMS. *In planta* expression of the *PKS19* gene cluster suggest a specific role for the derived chemical product during rice infection thereby adding a second novel metabolite, in addition to GAs, that may contribute to the ability of *F. fujikuroi* to uniquely infect rice.

## Results/Discussion

### The *F. fujikuroi* genome: General features

Whole genome shotgun sequencing of *F. fujikuroi* (strain IMI58289) by 454 pyrosequencing yielded 0.94 Gb of raw sequence data that was assembled into only 12 scaffolds (N50 of 4.2 Mb; 73 contigs spanning 43.9 Mb with an average read coverage of 19). A total of 14,813 gene models were predicted using a combination of gene prediction tools. Table 1 summarizes physical genome features which are similar to those of closely related species. To assess the completeness of the *F. fujikuroi* genome draft, we did BLAST searches with two separate highly conserved core gene sets from 39 and 21 higher eukaryotes species, respectively [40], [41]. None of the expected single-copy core orthologs were missing from the *F. fujikuroi* gene model set indicating that the core gene space has been completely covered.

**Table 1:** Comparative genome statistics.

	*F. fujikuroi*	*F. verticillioides*	*F. oxysporum*	*F. circinatum*	*F. graminearum*	*F. mangiferae*	*F. solani*
Genome size (Mb)	43.9	41.8	61.4	44.3	36.4	45.6	51.3
GC-content (%)	47.4	48.6	47.3	47.3	48.0	48.8	50.7
Protein coding genes	14813	14180	17458	15022	13826	16261	15702
Gene density (Number of genes per Mb)	338	339	284	339	379	356	306
Total exon length (Mb)	21.5	17.9	21.5	19.7	18.8	21.9	22.6
Total intron length (Mb)	1.9	2.3	3.0	1.8	1.9	2.1	2.7
Average distance between genes (kb)	1.4	1.5	2.1	1.5	1.1	1.3	1.7
Percent coding (%)	49.2	48.32	39.79	48.44	56.75	52.59	49.22
GC-content coding (%)	51.5	52.14	52.00	51.75	51.57	51.62	54.49
Average gene length (kb)	1.5	1.3	1.2	1.3	1.4	1.3	1.4
Mean protein length (amino acids)	484.7	419.6	409.7	436.5	453.1	447.8	479.6
Exons	41578	38477	46670	41023	38453	43543	48203
Average exon length (bp)	518.11	463.96	459.87	479.20	488.83	501.81	468.56
Exons/gene	2.81	2.71	2.67	2.73	2.78	2.68	3.07
Introns	26765	24297	29212	26001	24627	27282	32501
Average intron length (bp)	69.2	96.16	101.12	68.52	76.62	77.61	81.73

In order to predict protein functions and reconstruction of evolutionary genesis, a Similarity Matrix of Proteins (SIMAP) [42] was generated for the *F. fujikuroi* gene model set and then queried against the Swiss-Prot (UniProt Consortium, 2011) database. This analysis revealed 390 *F. fujikuroi* proteins with higher than 80% identity to proteins in the database, while 4,639 *F. fujikuroi* proteins had little similarity (<10%), indicating novel, species-specific proteins. In a bidirectional best hits (BBH) analysis of the protein set from *F. fujikuroi*, 71, 77 and 90% of the proteins were >50% identical to protein sets from the closely related species *F. verticillioides*, *F. circinatum* and *F. mangiferae*, respectively. In contrast, only 63% of the *F. fujikuroi* protein set had >50% identity to a *F. graminearum* protein set.

In *F. fujikuroi*, the annotated ORFs account for 49.2% of the genome with an average coding length of 1,457 nt and 2.8 exons per gene; the average exon length is 518 nt. The overall GC content is 47.4%, while the average GC content of ORFs is 51.5%. All of these key genome features are similar to those reported for *F. verticillioides* (Table 1).

Previous electrophoretic karyotype analysis of *F. fujikuroi* IMI58289 by contour-clamped homogeneous electric field (CHEF) gel electrophoresis led to the assignment of eleven chromosomes to seven bands. By Southern blot hybridization of the CHEF gel, eight genes were localized to either one or two of the separated chromosome bands [39]. Due to the high quality assembly of the *F. fujikuroi* genome into 12 scaffolds corresponding to the 12 chromosomes, we were able to assign these eight genes to specific chromosomes and to link the hybridization signals with the number of the chromosomes (Figure S1).

**Figure 2:**
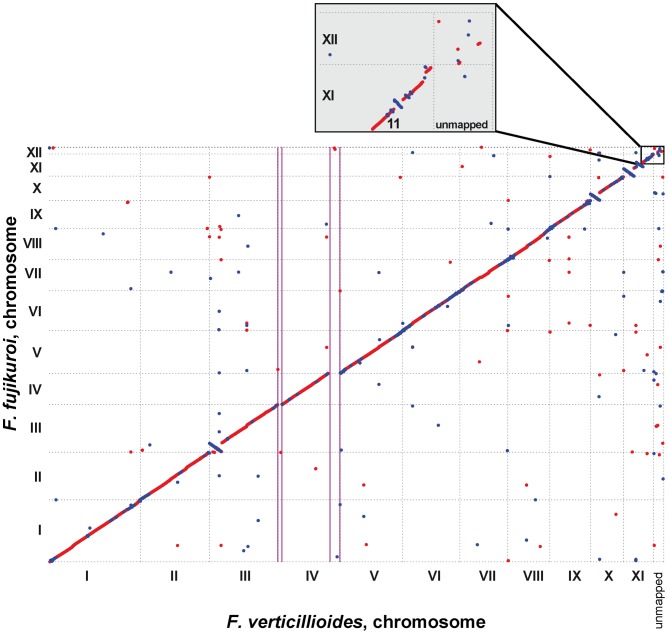
Whole genome comparison of *F. fujikuroi* with*F. verticillioides*. Dotplot of *F. fujikuroi* chromosomes and scaffolds against *F. verticillioides* calculated using MUMer [121] highlights overall collinearity. Orthologous DNA is represented by red dots, inverted segments are shown as blue dots. Inset magnifies *F. fujikuroi* chromosome XII, which has no homologue in the *F. verticillioides* scaffold set. The missing subtelomeric regions of chromosome IV in *F. fujikuroi* are highlighted by vertical purple lines. Dots that are located above or below the line indicating collinearity represent largely repetitive DNA.

Despite the shared synteny between the *F. fujikuroi* and *F. verticillioides* genomes [22], there are several significant differences worth noting. First, chromosome XII is present in the *F. fujikuroi* but absent in the genome sequence of *F. verticillioides* (Figure 2). However, the lack of chromosome XII in *F. verticillioides* may be strain-specific as it has been detected in other strains of *F. verticillioides* [38]. In *F. fujikuroi*, chromosome XII spans 693 kb and includes 173 predicted genes. A majority (139) of the corresponding predicted proteins lack sequence similarity to annotated Swiss-Prot proteins. Functional enrichment analysis using the MIPS ‘FunCat’ program [43] revealed that the remaining 35 predicted proteins are enriched (FDR<0.01) in the FunCat category ‘guidance of longitudinal cell extension and cell migration’ [43]. A second significant difference between the two genomes is that *F. fujikuroi* chromosome IV lacks 285 and 820 kb from the left and right arms, respectively, relative to *F. verticillioides*. In the latter species, the 285 and 820-kb regions collectively include 408 predicted genes that share no significant similarity to genes located elsewhere in the *F. fujikuroi* genome and are enriched in the FunCat categories ‘secondary metabolism’, ‘detoxification’ and ‘metabolism of melanin’ (FDR<0.01). There are also three smaller genomic regions in *F. verticillioides* that are absent in *F. fujikuroi*: a 70 kb segment on chromosome VII with 29 genes, a 22 kb segment on chromosome III with eight genes, and a 12 kb segment on chromosome V with six genes. The majority of these genes code for proteins of unknown function.

To determine whether the presence of chromosome XII and the significantly shorter chromosome IV are strain-specific features of *F. fujikuroi*, we analyzed nine additional *F. fujikuroi* isolates from different geographic regions by PCR. For analysis of chromosome XII, we employed three primer pairs that amplify fragments from the arms and near the center of chromosome XII. The results from this PCR analysis indicate that chromosome XII is most likely absent in two *F. fujikuroi* strains (m570 and C1995) from Japan, while only some regions of the chromosome are present in three strains (E289, E292 and E325) from Italy (Figure S2). The fact that all of the *F. fujikuroi* strains examined were isolated from infected rice plants and cause “bakanae” disease suggests that chromosome XII is not essential for pathogenicity on rice. Whether chromosome XII contributes to niche specificity, as has been observed for the supernumerary chromosomes of *F. oxysporum* and *Nectria haematococca* (*F. solani* MP VI) [22], [44], [45] is not yet known. Variability in presence and absence of chromosome XII in different *F. fujikuroi* isolates suggests that it might be an “accessory” supernumerary chromosome consistent with previous analyses of GFC species [38]. For analysis of chromosome IV, we designed two heterologous primer pairs based on *F. verticillioides* sequence present in the 285 and 820-kb regions of chromosome IV that were absent in *F. fujikuroi* IMI58289. All nine isolates of *F. fujikuroi* examined have the characteristic shorter chromosome IV observed in strain IMI58289 (Figure 2 and S2). Taken together, these results indicate that within *F. fujikuroi*, the shortened chromosome IV is species-specific, whereas the presence or absence of chromosome XII is strain-specific.

**Figure 3:**
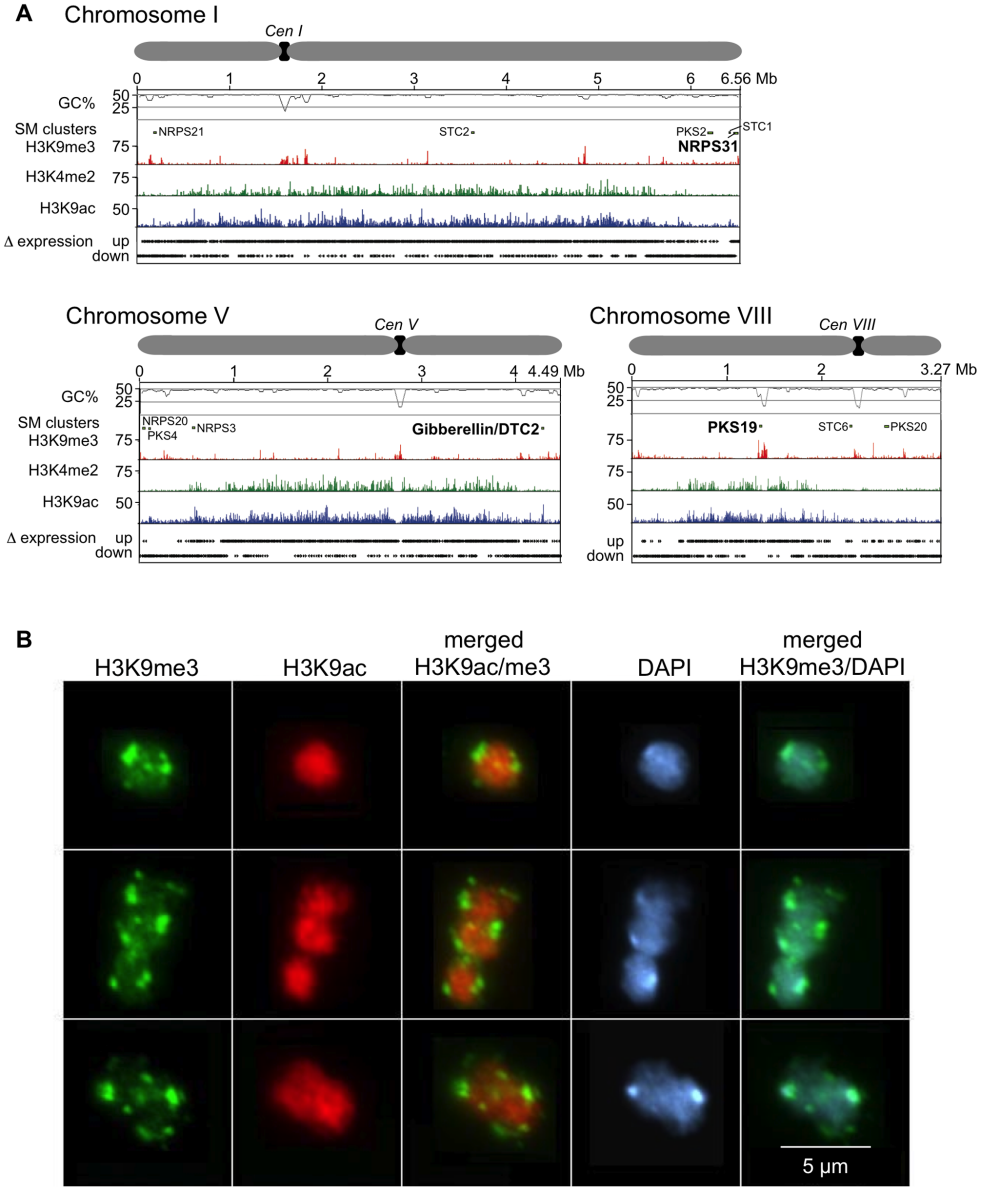
Characterization of *F. fujikuroi* chromosomes and variation in acetylation and methylation statues of histone H3. **A**: Information for chromosomes I, V and VIII is shown as examples of the 12 *F. fujikuroi* chromosomes (seeFigure S3 for additional chromosomes). For each chromosome, the position of the centromere is shown at the top; below this in descending order are: GC content, location of SM biosynthetic gene clusters, acetylation and methylation states of histone H3, and changes in gene expression. Variation in histone H3 modification statues indicates chromosomal regions in which genes are expressed (H3K9ac and H3K4me2) or silent (H3K9me3). “Δ expression up” indicates a more than twofold increase in gene expression during growth of *F. fujikuroi* in nitrogen-rich medium, whereas “Δ expression down” indicates an at least twofold decrease in gene expression. SM biosynthetic gene cluster locations are indicated by arrows labeled with the PKS, NRPS or TC (DTC means diterpene cyclase; STC means sesquiterpene cyclase) gene in each cluster (see Table 4 and Table S4). For the same analyses of other *F. fujikuroi* chromosomes, see Figure S3. **B**: Immunocytological analysis of histone acetylation and methylation in *F. fujikuroi*. Detection of specific histone markers was performed with H3K9me3 and H3K9ac-specific antibodies. DNA was counterstained with DAPI. H3K9me3 is significantly enriched in heterochromatin that forms several chromocenters, while H3K9ac is evenly distributed in the nuclei (scale bar = 5 µm).

Overall, the *F. fujikuroi* genome is low in AT-rich regions, as less than 5% of the genome consists of regions with more than 55% of A+T (Figure 3A). The main AT-rich region on each chromosome is the centromere (Table S1). After *Neurospora crassa* [46], *F. fujikuroi* is the second filamentous fungus with an essentially complete assembly of centromeric sequences. None of the other currently available *Fusarium* genomes contains more than short pericentric regions flanking the actual, yet unknown centromere sequences [46]. The positions of most centromeres are conserved in *F. fujikuroi* and *F. verticillioides*, and in many cases there is synteny of genes close to centromeres, even in the more distantly related *F. graminearum*, in which the genome has been condensed into four chromosomes (Table S2). Overall, centromeres of *F. fujikuroi* are significantly shorter than those of *N. crassa*, ∼50–80 kb compared to 150–280 kb, but similar in size to those of fission yeast [46], [47]. Centromeric DNA of *F. fujikuroi* resembles that of *N. crassa*, as it is rich in transposon relics but lacks highly repetitive alpha-satellite DNA that is typical of centromeric DNA of mammals and plants [46], [47]–[49]. Comparisons with other *Fusarium* species failed to reveal common centromeric or pericentric DNA sequences, suggesting that these regions can serve as lineage or species-specific markers. In *F. fujikuroi*, none of the terminal contig sequences are capped by telomere repeats, indicating that sequence data for all chromosome ends remain incomplete as for the other sequenced fungi.

### *In silico* analysis of prominent gene families and transposable elements revealed significant differences between related species

A genome-wide comparison of prominent gene families in *Fusarium* genomes suggest that some families are expanded while others are underrepresented in *F. fujikuroi* relative to other species.

The number of predicted transcription factors (TF) genes in *F. fujikuroi* is significantly higher (950 genes) in comparison to *F. verticillioides* (640), *F. circinatum* (841), and *F. oxysporum* (876), but almost identical to *F. mangiferae* (945), the closest relative of *F. fujikuroi* among the species examined (Table S3). The expansion of the total number of TFs in both *F. fujikuroi* and *F. mangiferae* is reflected in the Interpro domain group ‘fungal-specific TF/Zn(2)C6 fungal type DNA binding domain’ (IPR007219; IPR001138) (Table S3). This TF gene family is expanded to 235 in *F. fujikuroi* and 208 in *F. mangiferae* compared to 90 in *F. verticillioides* and 144 in *F. graminearum*. *F. fujikuroi* has 53 TFs that do not have a closely related homologue (less than 60% identity) in other fusaria. Interestingly, 33 out of these are predicted to encode Zn(2)C6 TFs (Table S3).

To determine the complete set of secreted proteins, including those secreted by both classical and non-classical pathways, we applied a combination of five bioinformatic approaches on the predicted protein sets for *F. fujikuroi* and four other fusaria. The number of proteins (1,336) in the predicted *F. fujikuroi* secretome is similar to those for the closely related GFC species *F. circinatum*, *F. mangiferae* and *F. verticillioides*, while *F. oxysporum* has 15% more proteins (1,541) in its secretome (Tables 2 and S3). Similarly, the number of predicted transporters, including ABC and MFS transporters, and all classes of substrate-specific permeases, are very similar in *F. fujikuroi* and other fusaria as is the number of histone-modifying enzymes (Tables 2 and S3).

**Table 2:** Occurrence of selected gene families and other genetic elements in genome sequences of seven *Fusarium* species.

	*F. fujikuroi*	*F. verticillioides*	*F. oxysporum*	*F. circinatum*	*F. graminearum*	*F. mangiferae*	*F. solani*
**Secondary Metabolite Biosynthetic Genes** a							
PKS	13 (1)	13 (0)	11 (1)	12 (1)	13 (8)	13 (0)	12 (8)
PKS/NRPS	4 (0)	3 (0)	3 (0)	3 (0)	2 (0)	3 (0)	1 (0)
NRPS	15 (1)	16 (0)	14 (1)	13 (1)	19 (9)	16 (0)	13 (4)
DMATS	2 (0)	2 (0)	2 (0)	4 (0)	0	4 (0)	0
TC	10 (0)	8 (0)	6 (0)	9 (0)	7 (1)	10 (9)	0
P450	143	130	168	145	114	116	156
**Secreted Protein-Encoding Genes**							
Secreted proteins (SP)	1336	1239	1541	1262	1264	1422	1337
Unique secreted proteins (simap-ratio<0.6)	72	168	416	203	450	7	756
Small secreted proteins (<300aa) (SSP)	512	531	694	567	548	586	510
SSP (% of SP)	38.32%	42.86%	45.04%	44.93%	43.35%	41.21%	38.15%
Non-classically secreted proteins (NCSP)	126	150	208	168	204	165	190
NCSP (% of SP)	9.43%	12.11%	13.50%	13.31%	16.14%	11.60%	14.21%
Small Non-classically secreted proteins (<300 aa) (SNCSP)	50	87	126	87	75	78	77
SNCSP (% of SP)	3.74%	7.02%	8.18%	6.89%	5.93%	5.49%	5.76%
**Transporter Genes**							
Transporters	857	840	995	895	673	679	979
ABC Transporters	65	70	77	65	63	33	73
Aminoacid permeases	99	103	126	108	86	70	126
Ammonium permeases	4	5	5	5	4	5	2
**Transcription Factor and tRNA Genes**							
Transcription factors	950	640	876	841	726	643	933
Unique transcription factors (simap-ratio<0.6)	53	77	253	118	726	314	530
tRNA genes	232	293	305	296	319	304	286
**Other Genetic Elements**							
Coverage by repeats	1.8 Mb	0.5 Mb	13.0 Mb	1.6 Mb	0.5 Mb	0.6 Mb	3.0 Mb
Coverage by repeats	4.08%	1.28%	21.24%	3.68%	1.31%	1.35%	5.85%
Transposable elements	2.20%	0.47%	4.76%	1.08%	0.33%	0.54%	1.64%

a SM gene predictions are based on InterPro domains, manually validated and corrected based on reports in the literature and on comparative analysis of fusaria. Values are the number of genes per genome; values in brackets are the numbers of genes unique to a genome among the species examined. Predicted genes were regarded as unique when they had either no match or an e-value≥10^−10^ in BLASTN analysis against available *Fusarium* genome sequences. PKS - polyketide synthase, NRPS - non-ribosomal peptide synthetase, DMATS - dimethylallyl tryptophan synthase, TC – terpene cyclase.

We also determined the coverage of transposable elements (TEs) by scanning for known transposons that have been reported to RepBase [50]. Additionally, we searched for novel TEs in a *de novo* approach which revealed two LTR-retrotransposons encoding a conserved reverse transcriptase or integrase and one DNA-transposon containing a predicted transposase. These three transposon families are not contained in RepBase (BLAST e value<10×10^−10^, bitscore >1,000) but were present in genome sequences of the closely related *F. oxysporum* and *F. mangiferae*. Overall, TEs constitute 2.2% of the *F. fujikuroi* genome, which is higher than in *F. verticillioides* (0.5%) but lower than in *F. oxysporum* (4.8%) (Table S3).

To estimate the genetic potential of *F. fujikuroi* to produce SMs, we identified genes predicted to encode five key classes of SM-associated enzymes: PKSs, NRPSs, TCs, DMATSs and P450s. The genes were identified by the presence of characteristic domains in predicted proteins and by BLAST analyses (Tables 2 and S4). We also examined flanking genes to identify putative gene clusters, which could include genes encoding TFs, transporters and modifying enzymes (e.g. dehydrogenases and acyl transferases) in addition to genes encoding the SM-associated enzymes noted above. These genes and potential gene clusters were then compared to homologous sequences in the genomes of *F. verticillioides*, *F. circinatum*, *F. mangiferae* and *F. oxysporum* as well as the more distantly related species *F. graminearum* and *F. solani* [22], [31]–[34], [45]. This analysis revealed that the *F. fujikuroi* genome comprises 17 genes that encode putative type I PKSs with canonical ketosynthase (KS) and malonyl-CoA:acyl carrier protein (ACP) transacylase (MAT) domains. Based on domain content, 14 of the predicted PKSs are reducing-type PKSs (R-PKS) in that they have the keto-reductase (KR), dehydratase (DH) and enoyl reductase (ER) domains that catalyze complete reduction of β-carbonyl during polyketide synthesis. Four of the R-PKSs include a NRPS module and are also referred to as PKS/NRPS hybrids. The remaining three PKSs are nonreducing-type PKSs (NR-PKSs) because they lack the KR, DH and ER domains. The *F. fujikuroi* genome also includes one type III PKS, an enzyme class typical for plants but that has recently been found in some bacteria and fungi [22], [51]. The analysis also revealed the presence of 15 NRPS, 2 DMATS, 10 TC (2 diterpene and 8 sesquiterpene cyclases) genes.

In total, the *F. fujikuroi* genome has the potential to encode 45 enzymes that could give rise to 45 structurally distinct families of SMs. SMURF analysis and the manual examination of genes flanking the 45 core SM genes indicate that most are part of a SM gene cluster. Finally, the analysis revealed that the *F. fujikuroi* genome encodes 143 putative P450s of which 28 are located in putative SM gene clusters (Table 2).

### Genome-wide distribution of histone marks

Recent studies in several fungi showed that chromatin modifications differ in regions with active (euchromatin) and silent (heterochromatin) gene transcription [52]–[54]. There are several examples demonstrating that SM gene clusters in fungi can be regulated by chromatin-modifying enzymes, a form of gene regulation that represents a general level for coordinated control of larger chromosomal segments [53]–[57]. Modifications of histone proteins can thus serve as markers for changes in chromatin structure associated with gene expression and silencing. For example, gene expression has been associated with acetylation of histone H3 lysine 9 (H3K9ac) and dimethylation of histone H3 lysine 4 (H3K4me2), whereas gene silencing has been associated with trimethylation of histone H3 lysine 9 (H3K9me3) [52]–[54]. In the current study, the presence of acetylated and methylated forms of histone H3 were determined across all *F. fujikuroi* chromosomes by ChIP-seq analysis with H3K9ac, H3K4me2 and H3K9me3-specific antibodies. As in *N. crassa*, H3K9me3 is mainly present near *F. fujikuroi* centromeres (Figures 3A and S3) as pericentric regions have higher levels of H3K9me3 compared to the putative centromeric core regions. Some of this decrease may be due to replacement of canonical H3 with the centromere-specific H3, CenH3, in the centromere cores [46], [47]. This pattern sets *F. fujikuroi* centromeres apart from dispersed large heterochromatic regions that show more uniform enrichment of H3K9me3 (Figures 3 and S3).

Immunofluorescence microscopy with H3K9ac- and H3K9me3-specific dyes revealed that nuclei of *F. fujikuroi* are highly enriched for H3K9ac (e.g. extensive euchromatin) and with no specific subnuclear distribution (Figure 3B). In contrast, H3K9me3 was concentrated in discrete areas of nuclei, predominantly at the periphery. Counterstaining with the DNA-binding fluorescent dye DAPI suggested that these are constitute heterochromatic regions, as previously observed in *N. crassa* [58]. H3K9me1 and H3K9me2 appear to be absent, at least under the conditions used in this study (data not shown).

All putative SM biosynthetic genes identified above were mapped to chromosome I to XI (none were present on chromosome XII) and most were located within subtelomeric regions, loosely defined as within several hundred kb of the putative end of a chromosome (Figures 3A and S3). These chromosomal regions are often subject to regulation by posttranslational modification of histones, including acetylation or methylation of the N-terminal tail of histone H3 [52]–[54], [59].

To assess whether the location of many SM gene clusters within subtelomeric regions corresponds to a punctate enrichment of H3K9me3 and are thus heterochromatic, we performed ChIP-seq analysis applying H3K9me3-specific antibody under nitrogen-limiting conditions that are favorable for the production of GAs and several other SM (Figure 3A). As mentioned above, H3K9me3 occurred largely in the pericentric and centromeric regions but each chromosome had several additional large regions with high levels of this mark for constitutive heterochromatin. All such regions were associated with AT- and transposon-rich DNA that had few, if any, annotated genes. None of the 45 putative SM enzyme-encoding genes and potential clusters was found in regions enriched for H3K9me3 suggesting that this mark is not important for the regulation of SM in *F. fujikuroi*. In contrast, H3K9me3 has been proposed to be critical for regulation of SM clusters in *Aspergillus nidulans* [53], [60]. When the two marks associated with active gene expression, H3K9ac and H3K4me2, were observed, no H3K9me3 marks were simultaneously present. H3K9ac and H3K4me2 occurred primarily at the centers or arms of most chromosomes under nitrogen-limiting conditions. Chromosomes X, XI and XII are unusual in that they nearly completely lack enrichment for H3K9ac and H3K4me2, which we attribute to the absence of expressed genes on these chromosomes, at least under the conditions tested. To visualize the global alterations in gene expression in response to a change in nitrogen availability, we mapped the chromosomal positions of genes that are up- or down-regulated two-fold or more, as determined by microarray analysis, in *F. fujikuroi* grown in high *vs.* low glutamine medium (Figure 3A). Overall, subtelomeric regions exhibited decreased expression in high nitrogen, while genes in regions associated with H3K4me2 and H3K9ac are generally induced in high nitrogen conditions.

### Comparative analysis of SM gene clusters: GA gene cluster structure and distribution

**Figure 4:**
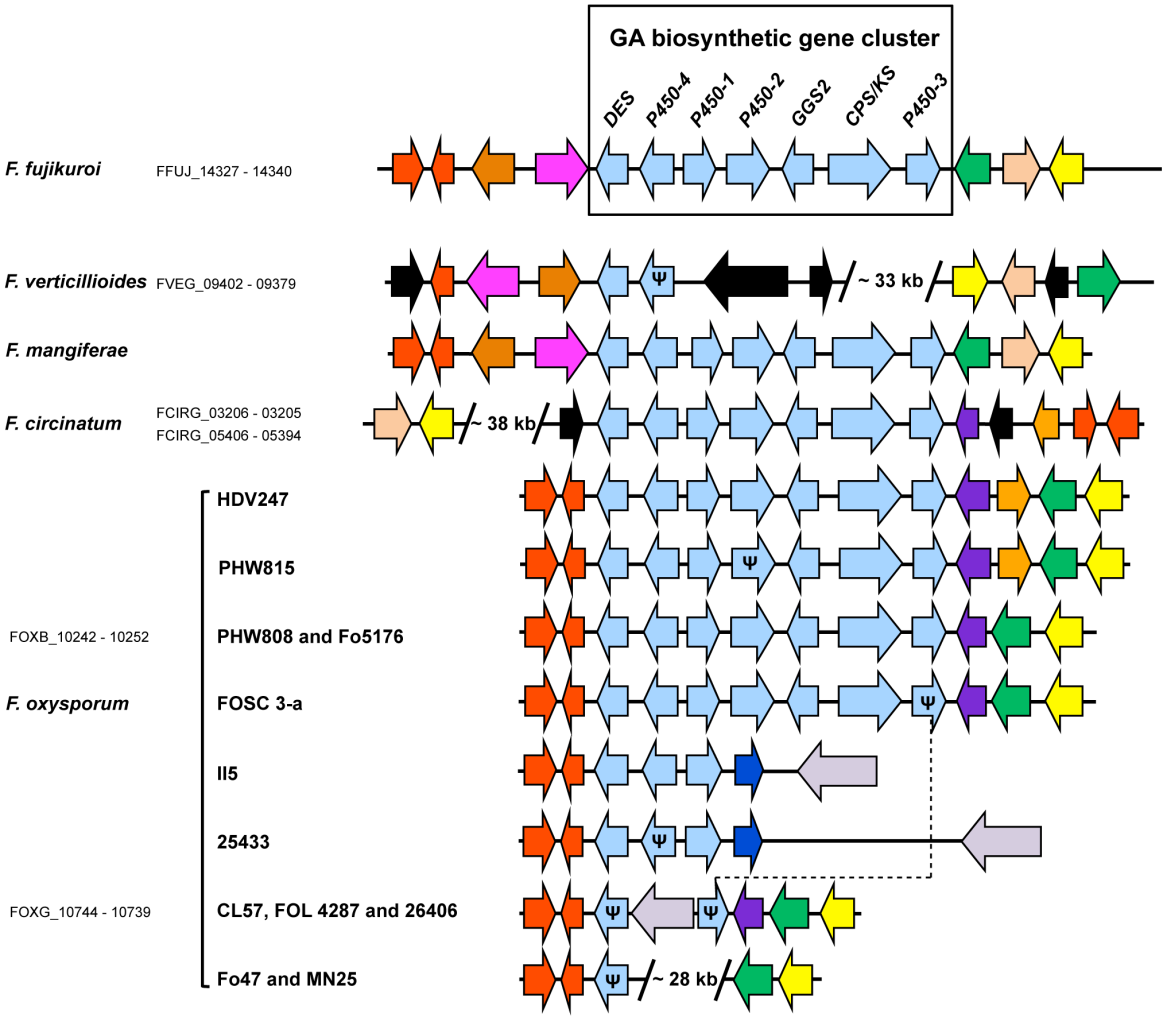
Comparison of GA biosynthetic gene clusters in *Fusarium* genome sequences. Arrows that are the same color represent genes, or gene sets, that have closely related homologues in two or more species/isolates. Light blue arrows represent GA biosynthetic genes. Black arrows represent genes that do not have closely related homologues in the GA cluster region of other species/isolates. Ψ indicates a pseudogene. For those that are available, gene/protein designations are indicated next to or below species names. FOXB and FOXG designations are for *F. oxysporum* isolates Fo5176 and Fol 4287, respectively.

SMs produced by fungi often play a role in triggering plant cell death and disease [61], [62]. Therefore, identification of the whole set of potential SM gene clusters can lead to the development of tools to investigate the role of toxins in disease development. The availability of genome sequences of *F. fujikuroi*, *F. circinatum* [35], *F. mangiferae* (S. Freeman and co-workers, this work), and eleven isolates of *F. oxysporum* (Broad Institute) provides an opportunity for a more comprehensive analysis of *Fusarium* SM biosynthetic genes than has been possible previously. Due to the association of GA production with “bakanae” disease of rice, GA biosynthetic genes are among the most extensively studied SM genes in *Fusarium fujikuroi*. Southern and PCR-based surveys indicate that the GA cluster, or parts of it, occurs in some species of the GFC (Figure 4) as well as some closely related species, e.g. *F. foetens*, *F. napiforme*, *F. miscanthi*, and *F. nisikadoi* [8], [63]. However, production of GAs has only been detected in isolates of *F. sacchari*, *F. konzum* and *F. proliferatum* [64], [65]. In addition, the cluster is absent in the more distantly related species *F. graminearum* and *F. solani* [66]. Here, analysis of genome sequences revealed that homologues of the entire *F. fujikuroi* GA cluster are present in *F. circinatum*, *F. mangiferae*, and five isolates of *F. oxysporum* (Figure 4). All intact clusters share the same gene order and orientation as the previously described clusters in *F. fujikuroi* and *F. proliferatum* [8], [63], [64]. In addition, all of the genes appear to encode functional proteins, with two exceptions: the coding regions of *P450-2* and *P450-3* in *F. oxysporum* isolates PHW815 and FOSC 3-a, respectively, are interrupted by premature stop codons (Figure 4). The seven other *F. oxysporum* genome sequences have partial GA clusters consisting of one to three intact and partial (i.e. *pseudo*) GA genes (Figure 4). While some GA cluster flanking regions share considerable synteny, there are marked differences in content and arrangement of flanking genes among other species (Figure 4). Although some of the flanking genes in *F. circinatum* are homologues of those in *F. fujikuroi*, the *F. circinatum* flanking region has a 38-kb insert and the GA cluster itself is inverted relative to *F. fujikuroi* (Figure 4).

Despite some differences in gene content and arrangement in the cluster-flanking regions, the presence of four conserved genes (FFUJ_14327, 14328, 14338, and 14340) in all species/isolates with an intact cluster suggests that significant synteny is conserved in the GFC and *F. oxysporum*. Furthermore, an ancient GA cluster was present in the ancestral *Fusarium* genome before divergence of GFC and *F. oxysporum*. This conclusion is supported by phylogenetic analysis that resolved *Fusarium* GA genes into a single clade that is distinct from GA genes in other fungi. This result indicates that the GA cluster in all *Fusarium* species examined likely evolved by vertical inheritance from a common ancestor (Figure S4). In addition, previously reported PCR and Southern data as well as genome sequence data analyzed here indicate that partial GA clusters are derived via similar patterns of gene loss in three phylogenetically distinct lineages of *Fusarium*: GFC, *F. oxysporum* and *F. minscanthi/F. nisikadoi* [8], [63]. In all three lineages, partial GA clusters always lack *P450-2*, *GGS2*, *CPK/KS* and *P450-3* (Figure 4). The similar patterns of gene loss in different *Fusarium* lineages indicate that degeneration of the cluster is not random. It is not clear what selective pressure(s) would drive non-random degeneration of the GA cluster, however, one possibility is that populations of each *Fusarium* lineage were introduced into an environment(s) or specified to a new host where GA production was disadvantageous.

Interestingly, functional GA clusters are also present in *Sphaceloma* (Dothideomycetes) and *Phaeospheria* (Dothideomycetes) [67], [68], both being only distantly related to *Fusarium* (Sordariomycetes). However, the evolutionary mechanisms by which these fungi acquired GA biosynthetic gene clusters are not yet clear. There is an increasing number of indications that the presence of homologous SM biosynthetic gene clusters in distantly related fungi can result from horizontal gene transfer (HGT) [69]. One example of this is evidence for HGT of the bikaverin biosynthetic gene cluster from *Fusarium* to the distantly related fungus *Botrytis cinerea* [70], [71].

### Gibberellins and their role in plant infection

Phylogenetic studies showed a broad distribution of the GA gene cluster among the genus *Fusarium*. However, nothing is known about the ability of *F. circinatum*, *F. mangiferae*, and *F. oxysporum* to produce GAs. Therefore, we studied the production of GAs by the different *Fusarium* strains under four culture conditions that varied in nitrogen availability and pH: 1) nitrogen deficient and acidic (6 mM glutamine); 2) nitrogen deficient and alkaline (6 mM nitrate); 3) nitrogen sufficient and acidic (60 mM glutamine); and 4) nitrogen sufficient and alkaline (60 mM nitrate). We also examined five recently sequenced strains of *F. oxysporum* which have an intact GA biosynthetic gene cluster in contrast to *F. oxysporum* 4287 (Figure 4). Despite the presence of the cluster, GA production was detected only in *F. fujikuroi*. Lack of GA production in other species could be caused by a number of factors, including mutations that leave ORFs intact but render enzymes nonfunctional, reduced transcription of GA genes, improper GA transcript processing and/or altered translation (Tables 3; S5A and S5B). Although GA production was not detected in *F. circinatum*, production of *ent*-kaurene, the first committed intermediate in the GA pathway, was detected (Table 3). The presence of this metabolite is consistent with the detection of transcripts for *CPS/KS*, which encodes *ent*-copalyl diphosphate/*ent*-kaurene synthase in *F. circinatum* (Figure S5).

**Table 3:** Presence of SM gene clusters and production of the concomitant chemical products under standard laboratory conditions.

Metabolite	*F. fujikuroi*	*F. verticillioides*	*F. mangiferae*	*F. circinatum*	*F. oxysporum*
**Gibberellins**	product	**no cluster**	**no product**	**no product**	**no product** *
**Bikaverin**	product	product	product	product	product
**Fusarubins**	product	product	product	**no product**	**no product**
**Fusarins**	product	product	**no cluster**	**no product**	**no cluster**
**Fumonisins**	product (low)	product	**no cluster**	**no cluster**	**no cluster**
**Beauvericin**	product	**no cluster**	product	product	product
**Fusaric Acid**	product	product	product	product	product

* GA production only in transformants carrying the *F. fujikuroi* GA gene cluster.

To determine whether fusaria with a remnant of the GA gene cluster have retained the regulatory mechanisms required for GA production, we transformed *F. oxysporum* 4287 with a cosmid clone carrying a wild-type copy of the *F. fujikuroi* GA gene cluster. As in previous experiments with *F. verticillioides* [66], transformants of *F. oxysporum* 4287 with the *F. fujikuroi* GA cluster produced GAs at levels similar to those produced by *F. fujikuroi* IMI58289 (Table S5B).

**Figure 5:**
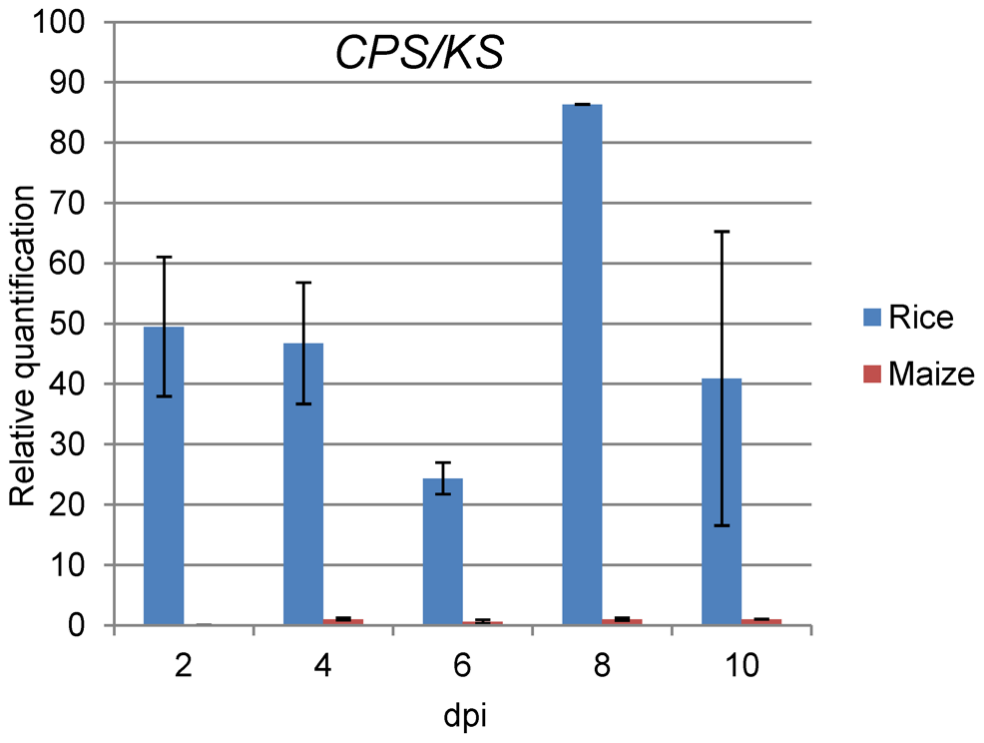
Relative expression of *CPS/KS* gene in rice and maize roots. Rice and maize roots were infected with *Fusarium fujikuroi* spores and every 2 days RNA was isolated from three or five plants and used in real time PCR analysis. The expression levels were obtained using the delta-delta Ct and were normalized against three reference genes encoding a related actin (FFUJ_05652), a GDP-mannose transporter (FFUJ_07710) and ubiquitin (FFUJ_08398). The expression levels of *CPS/KS* at 4 days in maize was arbitrarily set as 1, and all other expression levels were reported relative to it.

To explore if plant signals can induce GA gene transcription, we examined the expression of GA genes of *Fusarium* with an intact GA gene cluster (e.g. *F. mangiferae*, *F. circinatum* and some *F. oxysporum*) during infection of maize by qPCR. No GA gene expression was observed in these fusaria, except for low *CPS*/*KS* expression levels in *F. mangiferae* (data not shown). We also analyzed the expression of *CPS*/*KS* by *F. fujikuroi* during growth on the preferred host plant rice as compared to the non-preferred host maize. As expected, significantly higher expression for *CPS*/*KS* was observed in rice than in maize (Figure 5). These differences in GA gene expression suggest a dependency on specific rice signals as expected for the bakanae fungus.

**Figure 6:**
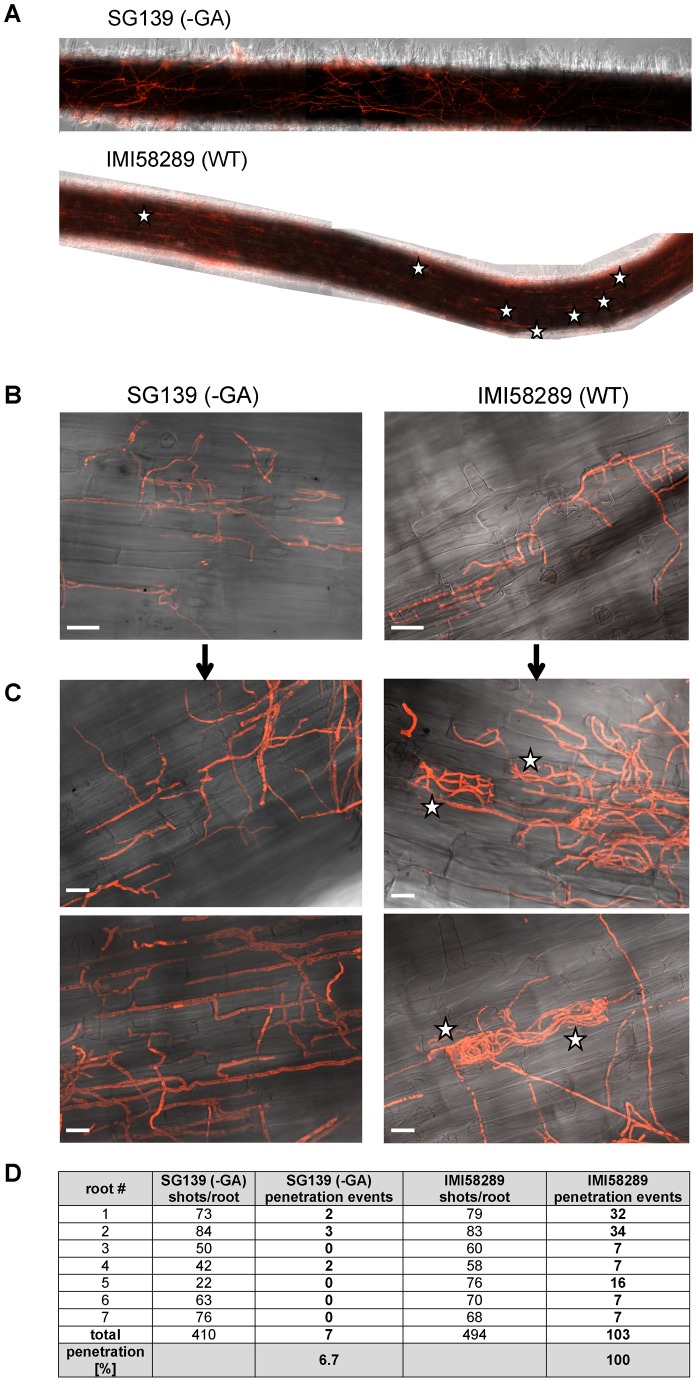
Infection of roots of rice plants by GA-producing wild-type (IMI58289) and GA-deficient (SG139) strains of *F. fujikuroi*. Rice plants were co-cultivated with either strain of *F. fujikuroi* for 7 days at 28°C and 80% humidity. Images of the corresponding whole plants following the incubation period are shown in **Figure 1Bf**. Both the wild-type strain and GA-deficient mutant were engineered to express the dsRed fluorescent protein. Stars indicate the position of cells that have been penetrated by and are filled with hyphae of *F. fujikuroi*. **A**: Microscopic overviews (10-fold magnification) of a root infected with the mutant SG139 (above) or wild-type. Note the absence of invaded cells in the root infected with the GA-deficient mutant. Images are an overlay of images from brightfield and Texas Red filter, which captures fluorescence emitted by the DsRed protein. **B/C**: 40-fold (B) and 63-fold (C) magnified images of roots infected with GA-deficient mutant (left) or wild-type strain (right). In most cases, hyphae of the mutant strain were observed between cells, in ‘intercellular’ spaces, whereas wild-type hyphae were often observed inside the cells (indicated by stars; see also Figure 1Bd) as well as in intercellular spaces. White lines in the images are scale bars corresponding to 200 µm (B) or 50 µm (C). **D**: Quantification of penetration events per rice root infected with wild-type or SG139, respectively. In addition, the total number of shots per root taken for analysis as well as penetration events of the mutant compared to the wild-type are shown.

Although “bakanae” disease was described more than 100 years ago, the role of GAs in pathogenesis of *F. fujikuroi* on rice is not well understood. To determine whether GA production is essential for pathogenesis, we compared the ability to infect and invade rice roots between the GA-producing wild type strain (*F. fujikuroi* IMI58289) and the nonproducing mutant SG139 that is missing the entire GA gene cluster. Microscopic analysis of infected rice roots revealed that the two strains can equally penetrate the rice root epidermis. Both strains also show the same apoplastic growth behavior within the parenchyma cells of the epidermis and the cortex (Figure 6B). However, the total number of successfully invaded symplasts per rice root differed significantly (Figure 6A,C). While we found 103 events of invasive fungal growth of the wild type inside the symplasts of rice root cells, only seven comparable events by the GA-deficient strain in seven independently analyzed roots were observed. Based on these results, we conclude that GAs contribute to the ability of *F. fujikuroi* to grow invasively in symplasts of parenchyma cells of rice epidermis and cortex rather than play a role in initial root colonization (Figure 6A,B,C).

In conclusion, our studies on GA production and GA gene expression clearly show that despite the presence of intact gene clusters in multiple *Fusarium* species/isolates, the ability to produce GAs and express GA biosynthetic genes is limited to the species *F. fujikuroi* and may provide a selective advantage during infection of the preferred host plant, rice. In addition, presence of a functional GA gene cluster is not essential for *F. fujikuroi* to colonize rice root cells, but appears to contribute to further invasion in rice tissue.

### Comparative analysis of PKS and NRPS biosynthetic gene clusters

To study the relatedness of *F. fujikuroi* PKS genes to those from other available *Fusarium* genomes, we performed a phylogenetic analysis of the predicted KS and MAT domains present in the predicted PKS proteins. The analysis also included KS and MAT domains from other fungal PKSs for which the SM products are known (Figure S6). *Fusarium* genomes contain 13–17 PKS genes, but only three are present in all genomes examined: *PKS3*/*FSR1*/*PGL1* (FFUJ_06826) which is responsible for production of the fusarubin-derived perithecial pigments [15], [31], [72], [73], and *PKS2* (FFUJ_00118) and *PKS7* (FFUJ_06260) for which the SM products are not known.

Four other PKS genes are only present in GFC species and *F. oxysporum*: *PKS1* (FFUJ_02219), *PKS4/BIK1* (FFUJ_06742), *PKS6/FUB1* (FFUJ_02105), and *PKS9* (FFUJ_14695). *BIK1* is responsible for bikaverin production in *F. fujikuroi* [14], [74], and *FUB1* is required for fusaric acid production in *F. verticillioides* and *F. fujikuroi* [75] (Niehaus et al., unpublished). The organization of the fusarubin, fusaric acid, and bikaverin gene clusters is almost perfectly maintained within the GFC species, and genes flanking at least one side are collinear in all species [76] (Figures S7, S8, S9).

*PKS10*/*FUS1* (FFUJ_10058), is required for fusarin production in *F. graminearum*, *F. verticillioides*, *F. fujikuroi* and the distantly related entomopathogenic fungus *Metarhizium anisopliae* [16], [18], [31], [75], [77], [78] (Niehaus et al., unpublished). *FUS1* is part of a nine-gene fusarin biosynthetic gene (FUS) cluster that is located in distinct genomic locations in all analyzed genomes (Figure S10). *F. mangiferae* lacks this cluster but has a partial, non-functional *FUS1* that is present in a similar syntenic region as the fusarin cluster in *F. verticillioides* rather than the syntenic region of the cluster in the more closely related *F. fujikuroi*. Comparison of gene organization of the *FUS* cluster among genomes indicates that the cluster has undergone at least one major rearrangement during its evolutionary history. *F. fujikuroi*, *F. verticillioides* and *F. graminearum* have one arrangement of cluster genes, while *F. circinatum*, *F. solani* and *M. anisopliae* have a different arrangement of cluster genes (Figure S9). Phylogenetic analyses of *Fusarium* PKSs, including FUS1, (Figure S6) suggest that the second arrangement, present in both *M. anisopliae* and some fusaria, is ancestral and that the rearrangement occurred in *Fusarium* after it diverged from *Metarhizium*. Possible reasons for the different cluster arrangement in the monophyletic species *F. fujikuroi* and *F. circinatum* could either be that the cluster was obtained by a progenitor of either species by HGT or the cluster rearranged in the same manner on multiple independent occasions. Although the SM product of the *FUS1* homologue in *Trichoderma reesei* is unknown, the presence of two additional *FUS* gene homologues (*FUS2* and *FUS3*) in this fungus (Figure S10) suggests that it is structurally related to fusarin.

**Figure 7:**
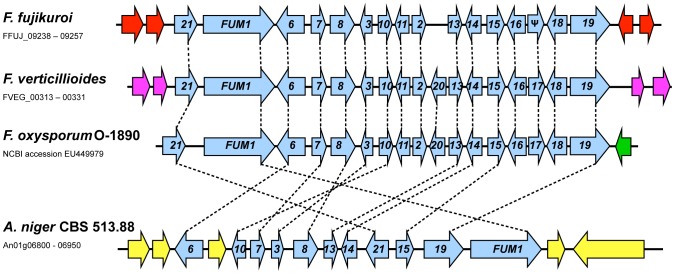
Comparison of the fumonisin biosynthetic gene (*FUM*) cluster in *F. fujikuroi*, *F. verticillioides*, *F. oxysporum* and *Aspergillus niger*. Horizontal arrows that are the same color represent genes, or gene sets, that have closely related homologues in two or more fungi. Blue horizontal arrows represent *FUM* genes, and the numbers in them correspond to *FUM* gene designations rather than designations from genome databases. Ψ indicates a pseudogene. Gene content and order is well conserved in the *Fusarium* species but less conserved in *A. niger*.

Similar to the fusarin cluster, the gene cluster responsible for fumonisin production is present at distinct genomic positions in *F. verticillioides*, *F. fujikuroi*, *F. oxysporum* (O-1819) and *A. niger* [79]–[82] (this work) (Figure 7). In contrast to the fusarin cluster, the synteny of the fumonisin cluster is perfectly conserved in the three fusaria, with the exception that *FUM20* is absent and *FUM17* is present as a pseudogene in *F. fujikuroi*. In *A. niger* the FUM gene cluster consists of eleven genes arranged markedly different than in *Fusarium* (Figure 7). The presence of the FUM cluster in *Aspergillus* has been attributed to horizontal gene transfer from a distantly related Sordariomycete [83].

Two *F. fujikuroi* PKS genes of unknown function are located 15 kb apart: *PKS17* (FFUJ_12066) and *PKS18* (FFUJ_12074) (Table S4). Homologues of *PKS17* and *PKS18*, as well as their flanking genes, are present in *F. mangiferae*, and the arrangement of the homologous genes is highly conserved in the two species (Figure S11). PKS17, a reducing PKS, and PKS18, a non-reducing PKS, share homology to the two PKSs, AfoG and AfoE respectively, involved in asperfuranone biosynthesis in *Aspergillus nidulans* [84] (Figure S6). In the proposed biosynthetic pathway for asperfuranone, the C-8 polyketide product of AfoG binds to the starter unit: ACP transacylase (SAT) domain of AfoE which then completes the synthesis of the asperfuranone precursor [84]. The lack of a SAT domain in PKS18 suggests that either the protein is non-functional or that the malonly:ACP transacylase (MAT) domain serves to bind both starter unit, potentially synthesized by PKS17, and extender units during polyketide synthesis.

**Figure 8:**
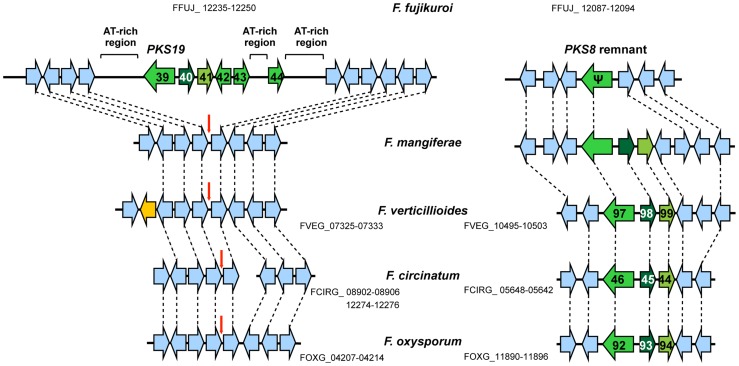
Comparison of the PKS19 (left) and PKS8 (right) clusters in genome sequences of *Fusarium*. Horizontal arrows that are the same color represent genes, or gene sets, that have closely related homologues in two or more fungi. The putative *PKS19* cluster is embedded within an AT-rich region, consists of six genes (FFUJ_12235–12250; horizontal green arrows), and among the species examined is unique to *F. fujikuroi*. However, synteny of genes corresponding to *PKS19* flanking genes in *F. fujikuroi* is highly conserved in *F. mangiferae*, *F. verticillioides*, *F. circinatum* and *F. oxysporum*. The vertical red arrows indicate the genomic location corresponding to the location of the *PKS19* cluster in *F. fujikuroi*. The putative *PKS8* cluster consists of three genes (green horizontal arrows). Only a remnant of *PKS8* is present in *F. fujikuroi*, whereas the intact *PKS8* cluster is present in the other species examined. The synteny of the *PKS8* cluster-flanking genes is partially conserved among the fusaria examined. For those that are available, gene designations are indicated below species names.

We also identified a PKS gene (FFUJ_12239 = *PKS19*) in *F. fujikuroi* that lacks a closely related homologue in the other *Fusarium* genomes (Figure S6; Figure 8; Table S4). *PKS19* is part of a putative six-gene SM cluster (PKS19 cluster) that is embedded within an AT-rich region of chromosome VIII (Table S4; Figure 3A; Figure 8). Like *PKS19*, the five other genes in the putative PKS19 cluster do not have closely related homologues in other *Fusarium* genome sequences examined. However, genes flanking the AT-rich region have closely related homologues, arranged in a highly syntentic manner, in the other fusaria (Figure 8). Together, the presence of the cluster in *F. fujikuroi*, its absence in closely related species, and the high level of synteny of the cluster flanking genes suggest that the PKS19 cluster was introduced into the *F. fujikuroi* genome relatively recently, possibly by HGT.

Although there are no closely related homologues of *F. fujikuroi* PKS19 in the *F. circinatum*, *F. mangiferae*, *F. verticillioides* or *F. oxysporum* genome sequences, the genomes have *PKS8* homologues, which have 55–58% nucleotide identity to *PKS19* (Figure S6). In addition, the two genes adjacent to *PKS8* are predicted to encode the same types of proteins as the two genes adjacent to *PKS19* (DltD-domain protein and a ToxD-like protein; Figure 8), even though the nucleotide identities for the genes between species are low, 52–60%. These observations suggest that *PKS8* and *PKS19* and the two adjacent genes constitute homologous, but relatively distantly related, biosynthetic gene clusters. Given the similar gene content of the two clusters, their SM product(s) could be similar in structure and perhaps even biological activity. The distribution of the PKS8 and PKS19 clusters is mutually exclusive among the species examined. However, *F. fujikuroi* has a remnant of *PKS8* in a genomic region that is syntenic to the regions flanking the PKS8 cluster in the other fusaria (Figure 8). This suggests that an ancestral *F. fujikuroi* genome had an intact PKS8 cluster that subsequently degenerated. It is not clear whether intact PKS8 and PKS19 clusters existed simultaneously in the ancestral genome.

Homologues of the *PKS12* gene (FFUJ_10347) have a pattern of distribution similar to that of *PKS8* and *PKS19*. Phylogenetic analysis resolved two types of *PKS12* homologues, *PKS12* and *PKS12a* (Figure S6), but the nucleotide identity between the types is low (58–66%). The presence of a methyltransferase-encoding gene upstream of *PKS12* and *PKS12a* suggests a two-gene SM biosynthetic cluster (Figure S12). Distribution of the PKS12 and PKS12a clusters is mutually exclusive in the fusaria examined. However, *F. oxysporum* strain Fol4287 has both an intact PKS12a cluster and remnants of the PKS12 cluster (Figure S12) in a region that is syntenic to the flanking regions of the PKS12 cluster in other fusaria. This indicates that a PKS12 cluster was present in an ancestral *F. oxysporum* genome and subsequently degenerated similarly to the PKS8 cluster. Complexity of PKS12/PKS12a-cluster evolution is further evident by the presence of two paralogues of the PKS12a cluster in *F. oxysporum* strain Fol4287 (Figure S12). Apart from these paralogues, the similarities in distribution of the PKS12/PKS12a and PKS8/PKS19 clusters among GFC and *F. oxysporum* raise the question if distribution of distantly related homologous PKS clusters is mutually exclusive, and this is exclusivity related to similarities in structures and or biological activities of the cluster SM products. Elucidation of the SM products of the PKS12/PKS12a and PKS8/PKS19 clusters and analysis of the distribution of these clusters in additional fusaria should aid in answering this question.

In order to assign some *Fusarium* NRPSs to a putative function, a BLAST analysis of the predicted NRPSs identified in the *Fusarium* genomes was conducted. A homologue of NRPS1 (Table S4), responsible for production of the siderophore malonichrome associated with iron uptake and present in *F. roseum*, *F. graminearum*, *F. culmorum* and *F. pseudograminearum* [85]–[87] is absent in *F. fujikuroi* and *F. mangiferae*. The homologous gene in *F. verticillioides* is located on the portion of chromosome IV that is missing in both *F. fujikuroi* (Figure 2) and *F. mangiferae*. The ability of *F. fujikuroi* to thrive despite missing an NRPS1 homologue likely reflects the underlying redundancy in iron uptake systems recently described [88].

*F. fujikuroi* NRPS22 (FFUJ_09296) provides an example of some challenges associated with predicting NRPS function. *F. fujikuroi*, *F. mangiferae*, *F. circinatum* and *F. oxysporum* possess an NRPS (NRPS22) homologue with two adenylation (A)-domains (Table S4; Figure S13). The first is most similar to the first A-domain in the enniatin NRPS, EsyN, previously described in *F. lateritium*, *F. sambucinium* and *F. scirpi* [89], [90] while the second NRPS22 A-domain is more similar to the second A-domain in the beauvericin NRPS, BeaS, from *Beauveria bassiana* [91]. Recent functional characterization of NRPS22 in *F. oxysporum* revealed that it is required for beauvericin production [92] and which immediate suggests that the *F. fujikuroi*, *F. mangiferae* and *F. circinatum* NRPS22 homologues also confer the ability to produce beauvericin rather than enniatin. It is interesting to note that the first A-domain in BeaS and EsyN is responsible for activation of the same amino acid and that this domain in the *F. fujikuroi*, *F. mangiferae*, *F. circinatum* and *F. oxysporum* homologues is more similar to EsyN than to BeaS (Figure S13).

**Figure 9:**
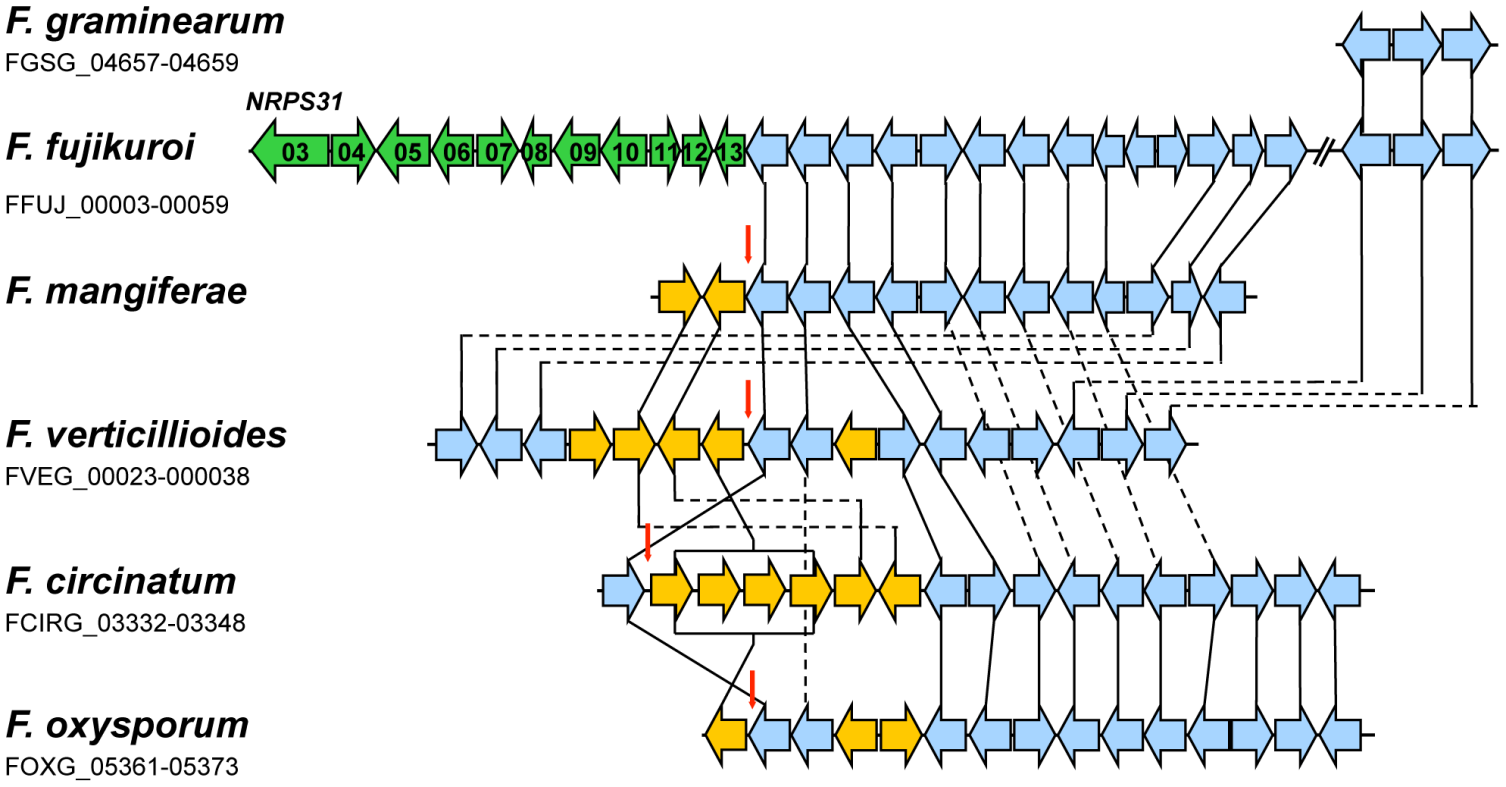
Comparison of genes flanking the putative 11-gene NRPS31 cluster in *F. fujikuroi* with homologous regions in genome sequences of other *Fusarium* species. Horizontal arrows that are the same color represent genes, or gene sets, that have closely related homologues in two or more fungi. Exceptions are indicated by green arrows, which represent NRPS31-cluster genes. Blue arrows represent NRPS31 cluster-flanking genes, and yellow arrows represent genes that do not have closely related homologues in the NRPS31 cluster-flanking region of *F. fujikuroi*. The vertical red arrows indicate the genomic location corresponding to the location of the NRPS31 cluster in *F. fujikuroi*. For those that are available, gene designations are indicated below species names.

Like PKS19, NRPS31 (FFUJ_00003) was present in the *F. fujikuroi* genome sequence but not in the sequences of the other *Fusarium* species examined (Figure 9). However, there is syntenic conservation of the regions flanking the NRPS31 cluster in the genome sequence of all the other *Fusarium* species except for *F. solani* (Figure 9). Functional characterization of this gene cluster will be shown below.

The portion of chromosome IV in *F. verticillioides* that contains NRPS1 and that is missing in *F. fujikuroi*, also contains a DMATS (FVEG_12218 = DMATS4 in Table S4). As noted above for NRPS1, the lack of this DMATS in *F. fujikuroi* implies that it is either redundant or that it is not required for growth.

In summary, the presence of highly similar PKS and NRPS gene clusters or remnants of PKS and NRPS-encoding genes in *Fusarium* species is an indication for their presence in ancestral *Fusarium*, and the absence of these clusters in other fusaria indicates a subsequent complete or partial loss during the evolution of the genus. On the other hand, the presence of highly similar gene clusters in distantly related fungi, probably distributed by HGT, can facilitate identification of their products.

### Does the presence of PKS and NRPS gene clusters correlate with product formation?

In addition to GAs, culture filtrates of the five *Fusarium* strains (*F. fujikuroi* IMI58289, *F. circinatum* Fsp34, *F. mangiferae* MRC7560, *F. verticillioides* M-3125 and *F. oxysporum* 4287) were also analyzed for the PKS-derived metabolites bikaverin, various fusarins, *O*-methylfusarubin, fumonisin B_1_ and fusaric acid, as well as for the NRPS-derived metabolite beauvericin (Table 3; Table S6). The optimal conditions for the production of the different metabolites varied considerably. For example, fusarins were only produced under high nitrogen conditions (60 mM glutamine; 120 mM NaNO_3_), while bikaverin and fusarubins were only or mainly formed under acidic low nitrogen (6 mM glutamine) or alkaline low nitrogen (6 mM NaNO_3_) conditions, respectively (Table S6). Fumonisin B_1_ was produced under acidic low nitrogen (6 mM glutamine) by *F. fujikuroi* IMI58289 and *F. verticillioides*M-3125, both of which harbor the entire cluster. Surprisingly, *F. fujikuroi* produced only very low levels of fumonisin B_1_ compared to *F. verticillioides* despite the high (>90%) nucleotide sequence identity of *FUM* cluster genes in the two species (Table S6).

To determine whether the *F. fujikuroi* fumonisin cluster is induced *in planta* and to compare any expression observed with the expression of the *F. verticillioides* fumonisin cluster *in planta*, we examined the expression of the PKS-encoding gene *FUM1* on rice and maize in both fungi by qPCR. Although significant differences in the level of transcription were observed after growth on the two plant species, the pattern of expression were similar. Specifically, *FUM1* was more highly expressed on maize than on rice in both strains and more highly expressed by *F. verticillioides* than *F. fujikuroi* (Figure S14). The higher and specific expression on maize by *F. verticillioides* could reflect the requirement of fumonisin for late stages of infection [93], [94]. Interesting, although *F. fujikuroi* is not a major pathogen of maize, we detected higher expression of *FUM1 in planta* than *in vitro*, perhaps reflecting a similar *Fusarium* response to maize signals (Figure S14).

Overall, our data suggest that the presence of orthologous SM gene clusters in closely related *Fusarium* species does not always correlate with production of the corresponding SM(s), at least under laboratory conditions. For example, although *F. circinatum* Fsp34 has the FUS cluster, it did not produce fusarins, and while *F. oxysporum* 4287 has the *FSR* cluster, it did not produce *O*-methylfusarubin in our hands (Table S6) (Figures S10 and S7). Genes in some clusters are expressed only or at higher levels *in planta* than in culture, and in some cases, levels of expression *in planta* were dependent on the host plant, as shown for fumonisin and GA biosynthetic genes. Similar observations have been made in other fungi, e.g. in *A. nidulans* where non-standard growth conditions activated otherwise silent gene clusters [95], [96].

### Nitrogen availability affects the transcriptome, proteome and histone modification patterns

Since biosynthesis of multiple *F. fujikuroi* SMs is regulated by nitrogen availability and pH, we analyzed the transcriptome of the fungus grown under the same conditions used to assess SM production, namely acid low nitrogen, acid high nitrogen, alkaline low nitrogen, and alkaline high nitrogen using Roche-NimbleGen DNA microarrays. We used high quality 12×135 K Nimblegen microarrays that were manufactured based on the present genome annotation of *F. fujikuroi* IMI58289 and performed a genome-wide search for all nitrogen and/or pH-affected genes, but specifically focused on expression patterns of all annotated SM gene clusters (Table S4).

Based on the selection criteria 2-fold change in expression at the 95% confidence interval, 3,117 and 3,242 genes were up-regulated under acidic and alkaline low-nitrogen conditions, respectively. Up-regulation of 2,494 of these genes occurred under both low-nitrogen conditions; whereas up-regulation of 560 of the genes occurred only in the acidic condition, and up-regulation of 717 occurred only in the alkaline condition. 63 genes were up-regulated in the acidic condition but down-regulated in the alkaline low nitrogen condition; and 31 genes were up-regulated in the alkaline condition but down-regulated in the acidic condition (Figure S15, Table S7). ‘FunCat’ analysis indicated an overrepresentation of genes likely involved in transport, carbon metabolism, and detoxification among the low nitrogen-induced genes (Table S7). This included genes known to be nitrogen-repressed, e.g. the nitrite reductase *NIIA* (FFUJ_06099), the ammonium permeases *MEPA, MEPB and MEPC* (FFUJ_01144; FFUJ_11805; FFUJ_13836), and a nitrate transporter gene *CRNA* (FFUJ_00934) [11], [97], [98].

Under high-nitrogen conditions, there was up-regulation of many genes involved in primary metabolism, e.g. amino acid metabolism, DNA processing, transcription, transport, protein synthesis, and protein folding and modification (Table S7). In total, 3,860 and 4,192 genes were up-regulated under acidic and alkaline high-nitrogen conditions, respectively. Of those genes, 3,021 were up-regulated in both acidic and alkaline high-nitrogen conditions, while 808 were up-regulated only in the acidic and 1,108 were up-regulated only in the alkaline high nitrogen condition (Figure S15, Table S7).

We were able to identify at least one condition that induced relatively high levels of expression of genes within 30 of the 45 *F. fujikuroi* SM clusters: 13 clusters with a PKS gene, the two with a diterpene cyclase (DTC) gene, two with a sesquiterpene cyclase (STC) gene, 11 with an NRPS gene, one with a DMATS gene, and the one with the type III-PKS gene. These data are based on differences in expression of putative SM cluster genes in response to low and high nitrogen (Table S7). Among the SM gene clusters for which the corresponding SM product(s) has not yet been identified, genes in the PKS16, STC4, NRPS20, and NRPS21 clusters were expressed at higher levels under both acidic and alkaline low-nitrogen conditions (Tables 4 and S7). Genes in the NRPS3 cluster were expressed under the acidic low-nitrogen (glutamine) condition, whereas genes in the NRPS13 were expressed under the alkaline low-nitrogen (nitrate) condition.

**Table 4:** Expression pattern^a^ of the secondary metabolite biosynthetic gene clusters under four growth conditions.

Synthase enzyme encoded by cluster	6 mM Gln	60 mM Gln	12 mM NaNO_3_	120 mM NaNO_3_	Secondary metabolite	Reference
PKS1	+	−	+	−	n/k	
PKS2	−	++	−	+++	n/k	
**PKS3**	++	−	+++	−	**fusarubins**	[15]
**PKS4**	+++	−	−	+	**bikaverin**	[14]
**PKS6**	−	+++	−	+++	**fusaric acid**	[75]
PKS7	−	−	−	−	n/k	
**PKS10**	−	+++	−	+++	**fusarins**	[75], [78]
**PKS11**	+++	−	++	−	**fumonisins**	[79], [82]
PKS12	−	−	+	−	n/k	
PKS13	−	−	−	−	n/k	
PKS14	++	−	++	−	n/k	
PKS16	+++	−	+++	−	n/k	
PKS17/18	−	−	+	−	n/k	
**PKS19**	−	+	−	+	**new metabolite**	**this study**
PKS20	+	−	+	−	n/k	
PKS Type III	−	−	−	++	n/k	
NRPS2	−	++	−	+++	n/k	
NRPS3	+++	−	−	−	n/k	
NRPS4	−	−	−	−	n/k	
NRPS6	−	++	−	+++	n/k	
NRPS10	−	−	−	+++	n/k	
NRPS11	−	−	−	+++	n/k	
NRPS13	−	−	+++	−	n/k	
NRPS17	−	−	−	−	n/k	
NRPS20	+++	−	+++	−	n/k	
NRPS21	+++	−	+++	−	n/k	
**NRPS22**	+++	−	++	−	**beauvericin**	[92], this study
NRPS23	−	−	−	−	n/k	
NRPS25	+	−	−	−	n/k	
**NRPS31**	−	+++	−	−	**apicidin-like**	**this study**
**DTC1** b	+++	−	++	−	**gibberellins**	[8], [9], [39], [63]
**DTC2**	++	−	+	−	**neurosporaxanthine**	[13]
STC1	−	−	−	+++	n/k	
STC2	−	−	−	−	n/k	
STC3	−	−	−	−	n/k	
**STC4**	+++	−	+++	−	**α-acorenol**	[163]
STC5	−	−	−	−	n/k	
**STC6**	−	−	−	−	**caryophylene**	[163]
STC7	−	−	−	−	n/k	
STC8	−	−	−	−	n/k	
DMATS1	++	−	++	−	n/k	

a +++, >90% of the genes belonging to the cluster are expressed under the condition indicated. ++, 50–90% of the genes belonging to the cluster are expressed under the condition indicated. +, 25–50% of the genes belonging to the cluster are expressed under the condition indicated. −, 0–25% of the genes belonging to the cluster are expressed under the condition indicated.

b DTC and STC indicate diterpene synthase and sesquiterpene synthase, respectively.

Key enzymes of which the respective product is known are indicated in bold letters and the respective metabolites are listed; n/k indicates that the corresponding metabolite is not yet known. Red labeled key enzymes and corresponding metabolites are *Fusarium fujikuroi*-specific.

To determine whether transcription and protein levels are correlated in *F. fujikuroi*, we quantified whole cell protein extracts from the fungus grown under acidic low- and high-nitrogen conditions. To do this, we employed metabolic labeling and quantitative proteomics. The fungus was grown for three days under acidic low (6 mM glutamine) or high nitrogen (60 mM glutamine) conditions. High-nitrogen cultures were grown with ^14^N glutamine, whereas low-nitrogen cultures were labeled with ^15^N glutamine. ^14^N glutamine and ^15^N glutamine-grown mycelia were harvested, mixed on equal protein basis, and then fractionated by SDS-PAGE. Protein bands were excised, in-gel digested with trypsin, and peptides were then subjected to liquid chromatography coupled with high resolution mass spectrometry (LC-MS/MS). In total, two independent biological replicates (A and B) were analyzed resulting in the identification of 2,808 distinct proteins (18.9% of all predicted *F. fujikuroi* ORFs). 2,060 proteins were present in both biological replicates, and 1,644 of these could be quantified in both replicates. Of the 1,644 proteins quantified, 418 were down regulated (0.5-fold and lower), 618 exhibited weak or no change in abundance, and 347 were up-regulated (2-fold and higher). 261 proteins have a too high divergence between replicate A and B, resulting in missing mean ratios.

‘FunCat’ analysis indicated that proteins likely to be involved in ‘nitrogen, sulphur and selenium metabolism’ and ‘secondary metabolism’ were significantly overrepresented in the high-nitrogen condition (FDR<0.05), whereas proteins likely to be involved in ‘virulence, disease and defense’ and ‘secondary metabolism’ were significantly overrepresented in the low-nitrogen condition, considering differential expressed genes according to transcriptomics (2-fold higher or lower, FDR<0.05) and proteomics data (4-fold higher or lower). It is notable that the overrepresentation of ‘secondary metabolism’ proteins under both low and high-nitrogen conditions differs when allowing for transcriptomics data alone, where ‚secondary metabolism’ genes were not overrepresented in either condition. The detection of a moderate correlation between fold changes of transcript and protein ratios (Pearson = 0.45, Spearman = 0.36, p<0.01) indicate that distinctive transcriptional and post-translational control mechanisms exist in *F. fujikuroi* (Table S7). In *A. fumigatus*, a similar comparison of microarray and RNA-sequencing data with 2D-gel protein quantification data was recently presented [99]. These data underscore the power of combining transcriptome and proteomic analyses and indicate that LC-MS/MS protein quantification provides a greater set of quantifiable proteins than conventional 2D-gel approaches. For full proteome coverage, extensive subcellular and protein fractionation techniques will be required as described for the *S. cerevisiae* proteome project [100].

**Figure 10:**
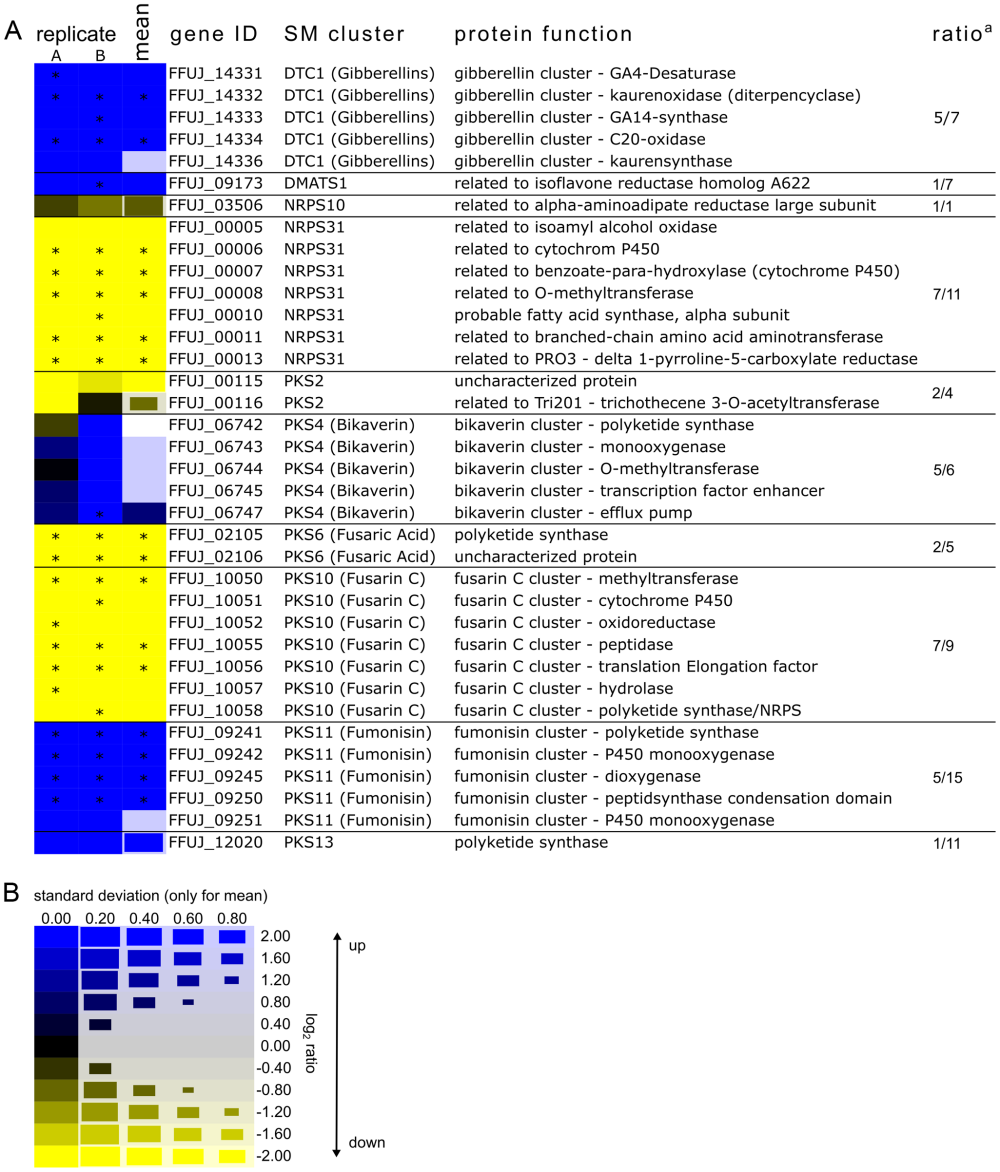
Changes in levels of selected proteins encoded by SM biosynthetic genes in *F. fujikuroi* as determined by comparative (−N/+N) quantitative proteomics. **A**: Increased (blue) and decreased (yellow) protein levels in response to nitrogen availability. Protein levels shown in columns A and B are data from independent experiments. Values are shown for only proteins quantified in both experiments. A log_2_ ratio>0 (−N/+N) indicates an increase in abundance in the low-nitrogen condition; a log_2_ ratio<0 indicates a decrease in abundance in the low-nitrogen condition; and a log_2_ ratio of zero indicates no changes in protein levels. Boxes with an asterisk indicate, that this protein could only be quantified in one nitrogen condition. The numbers in the far right column indicate how many proteins could be quantified within a cluster; value to the left of the slash is from replicate A, and value after the slash is from replicate B. **B**: Key to heat map showing Log_2_ values that correspond to different shades of blue and yellow. Standard deviation of a ratio is reflected in the size of the blue and yellow boxes.

Notably, we found proteins from ten of the putative SM clusters that were significantly enriched under acidic conditions with low or high nitrogen (Figure 10). There were six STC, three NRPS and two PKS clusters for which expression of genes and proteins was not detected under any of the four growth conditions examined suggesting that these clusters are not or not solely regulated by nitrogen availability or pH (Table 4; Figure 10). No proteins encoded by SM cluster genes were identified that are exclusively expressed under alkaline conditions (PKS2, STC1, NRPS2, NRPS6, NRPS10 and NRPS11) (Table 4) as the proteomic approach was performed only under acidic low and high nitrogen conditions.

As expected from our previous studies [10], GA cluster genes were expressed under low nitrogen conditions (Table 4 and S9), and this expression paralleled abundance of GA proteins (Figure 10). The fumonisin cluster was expressed under both acidic and alkaline low-nitrogen conditions, and accordingly five fumonisin proteins were identified under low-nitrogen conditions (Tables 4 and S7; Figure 10). Also, the recently reported expression of the fusarin C (*PKS10*/*FUS*) cluster under high nitrogen conditions [78] (E.-M. Niehaus et al., unpublished) was confirmed here by genome-wide microarray analysis and enrichment of the corresponding proteins (Table 4; Figure 10). Furthermore, gene expression of the NRPS31 cluster, which was observed only in the *F. fujikuroi* genome, was correlated with the presence of the corresponding proteins under acidic high-nitrogen condition. For the remaining SM clusters (PKS1, PKS12, PKS14, PKS17/18, PKS19, PKS20, NPS25, DMATS1 and PKS type III) only slight tendencies for regulation by nitrogen under one of the four growth conditions were observed (Table 4). Likewise, there was no significant enrichment of the corresponding proteins under any of the conditions examined (Figure 10).

Summarizing, we performed microarray analyses under our four standard conditions: low-nitrogen and high-nitrogen at both acidic and alkaline pH. We were able to identify at least one condition that induced expression of 30 out of the 45 SM gene clusters. Comparison of our microarray and proteomic analyses revealed a correlation between gene expression and presence of the corresponding proteins for nine of the SM gene clusters that were expressed under acidic low- or high-nitrogen conditions. Both expression of NRPS31 cluster genes and abundancy of the respective proteins were elevated under the acidic high-nitrogen condition. The tendencies of expression of SM cluster genes to correlate with the level of the corresponding proteins indicates that genome-wide expression analyses are a powerful approach for identifying conditions that induce production of unknown SMs.

### Does gene expression correlate with histone modifications?

As mentioned above, we determined whether there is a correlation between histone modifications and gene expression across the genome. This analysis also allowed us to examine whether such a correlation exists for genes throughout the *F. fujikuroi* genome as well as genes located in the 45 SM clusters (Figure 3; Figure S3). For this analysis, we performed ChIP-seq with two of the standard conditions: low- and high-nitrogen acidic conditions (6 mM and 60 mM glutamine, respectively) with antibodies specific to two activating (H3K9ac and H3K4me2) and one silencing (H3K9me3) modifications.

First, we examined whether gene expression was correlated with the presence of the histone activation marks (H3K9ac and H3K4me2), 1) expressed under acidic low-nitrogen conditions and the presence of activation mark H3K4me2, but repressed under acidic high-nitrogen and absence of activation mark H3K4me2, 2) repressed under acidic low-nitrogen and the absence of activation mark H3K9ac, but expressed under acidic high-nitrogen condition and presence of activation mark H3K9ac (Table S7), 3) repressed under acidic low-nitrogen and the absence of activation mark H3K4me2, but expressed under acidic high-nitrogen and presence of activation mark H3K4me2. There was a relatively low positive correlation between gene expression and the presence of the activating histone marks H3K9ac and H3K4me2 for all genes in the genome. Overall, the correlation with H3K9ac and expressed genes was better than that of H3K4me2 with expressed genes, which had been observed also by genome-wide analyses in *Trichoderma reesei* [101].

**Figure 11:**
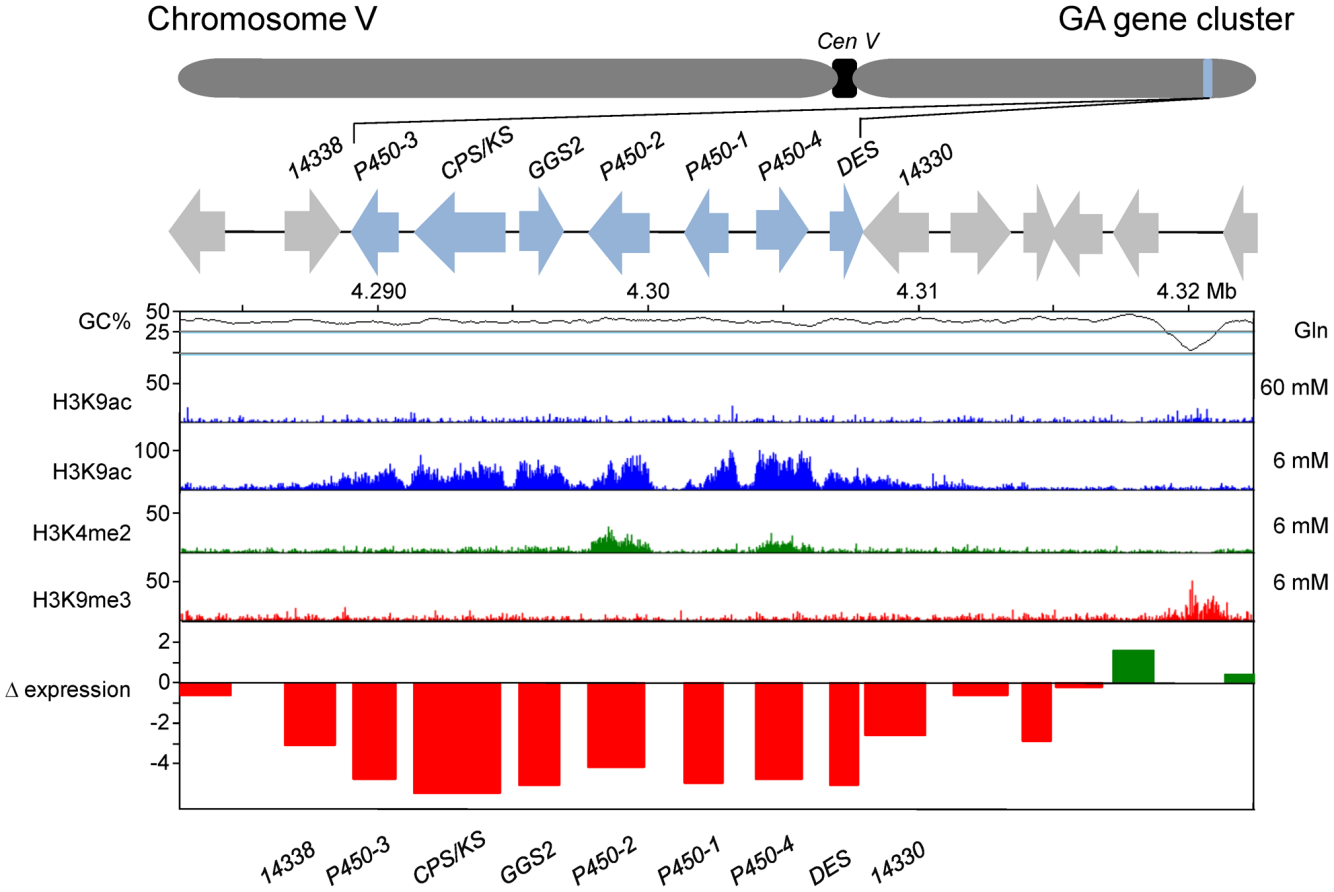
Location of the GA biosynthetic gene cluster on *F. fujikuroi* chromosome V as well as levels of histone modifications and gene expression within and flanking the cluster. Histone marks are described in the legend to Figure 3. Expression data were derived from microarray experiments in low (6 mM glutamine) and high (60 mM glutamine) nitrogen and are plotted as the changes in log_2_ expression values in high-nitrogen medium compared to low nitrogen medium. H3K9ac and gene expression are correlated, as both are decreased under high nitrogen conditions.

**Figure 12:**
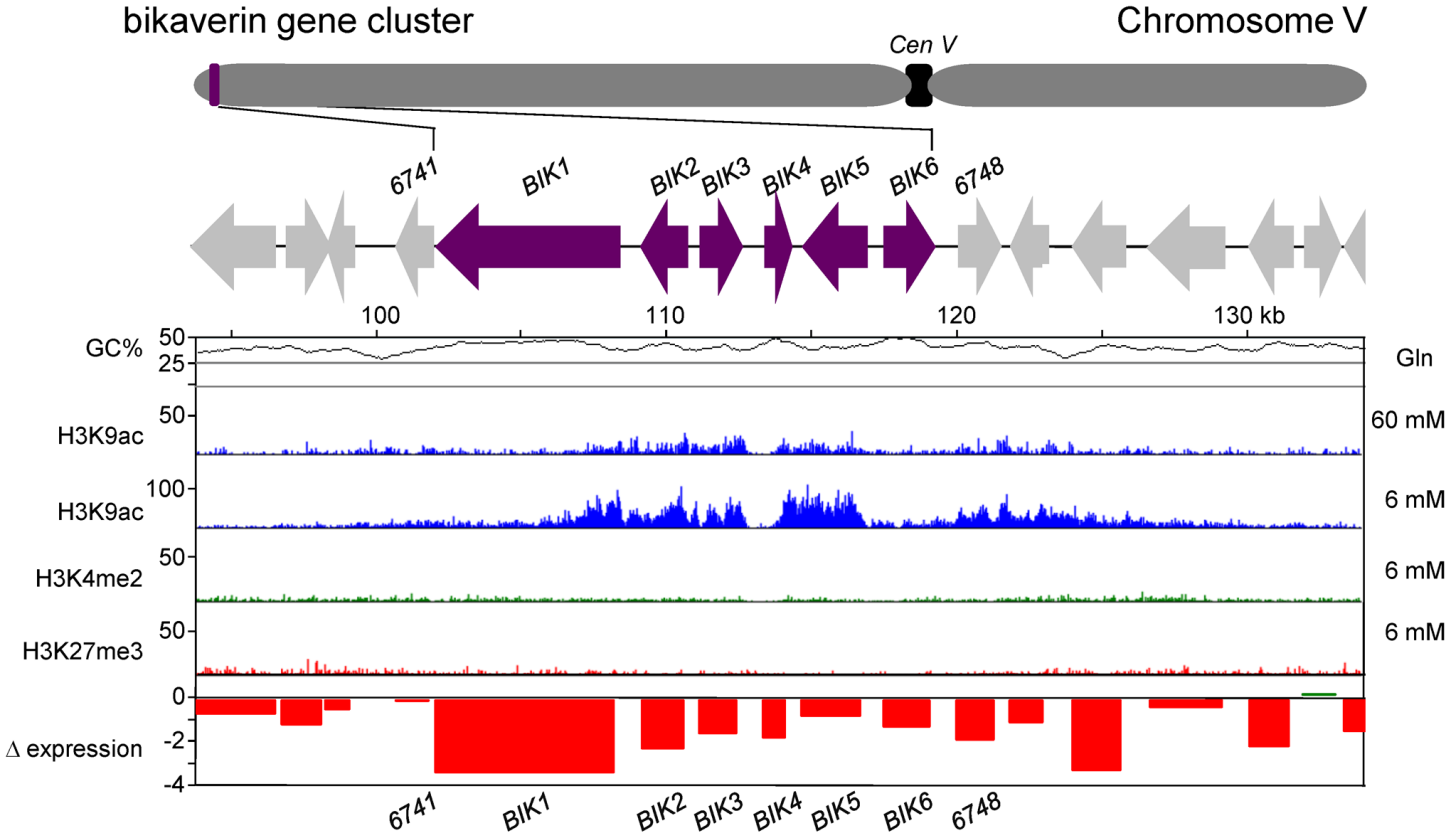
Location of the bikaverin biosynthetic gene cluster on *F. fujikuroi* chromosome V as well as levels of histone modifications and gene expression within and flanking the cluster. Histone marks are described in the legend to Figure 3. Expression data were derived from microarray experiments in low and high nitrogen and are plotted as the changes in log_2_ expression values in high-nitrogen medium compared to low-nitrogen medium. H3K9ac and gene expression are correlated, as both are decreased under high nitrogen conditions.

However, when comparing the presence of histone marks and transcriptome data specifically for SM gene clusters, the correlation was much better. Thus, the presence of the histone mark H3K9ac was correlated with gene expression across the GA gene cluster at the low-nitrogen condition, while H3K9ac was almost completely absent under the repressing high-nitrogen conditions (60 mM glutamine) (Table S7; Figure 11). The same histone pattern was observed for both the bikaverin (Figure 12) and fumonisin (Figure S16) genes clusters: enrichment of H3K9ac under inducing conditions (low nitrogen) and low levels of H3K9ac under repressing conditions (high nitrogen).

Taken together, these findings strengthen the hypothesis that histone acetylation is associated with gene transcription also in *F. fujikuroi*. A similar correlation between acetylation of histones associated with SM gene clusters and expression was also observed for other fungi, e.g. *A. parasiticus* and *A. nidulans* [51], [57], [102], [103]. However, not all gene clusters that exhibit histone acetylation are transcribed, and not all transcribed clusters exhibit histone acetylation. For example, the fusaric acid (PKS6) cluster exhibited little enrichment for H3K9ac under the inducing condition (Figure S16A).

In contrast to H3K9ac, H3K4me2 enrichment was observed only for the GA gene cluster. Here, two of the seven GA biosynthetic genes, *P450-4* and *P450-2*, were enriched for H3K4me2 under inducing low-nitrogen conditions (Figure 11). These results imply that the state of H3K9 acetylation, and also H3K4 methylation, alone is not sufficient for transcriptional activation but that other factors, including basal or specific transcription factors, additional histone modifications, or plant signals can also regulate expression of cluster genes. Furthermore, up-regulation of a gene cluster does not always result in high levels of production of the corresponding SM(s). For example, low-nitrogen conditions induce high levels of expression of fumonisin biosynthetic genes in both *F. fujikuroi* and *F. verticillioides*. Although this gene expression is accompanied by relatively high levels of fumonisin production in *F. verticillioides* it is accompanied by only low levels of production in *F. fujikuroi* (Table S6) [104].

In conclusion, analysis of the histone marks H3K4me2 and H3K9ac that are typically associated with sites of active transcription, combined with genome-wide expression analysis can serve as a powerful tool to identify new SM gene clusters and culture conditions that induce their expression.

### Discovery of two novel gene clusters in *F. fujikuroi*

**Figure 13:**
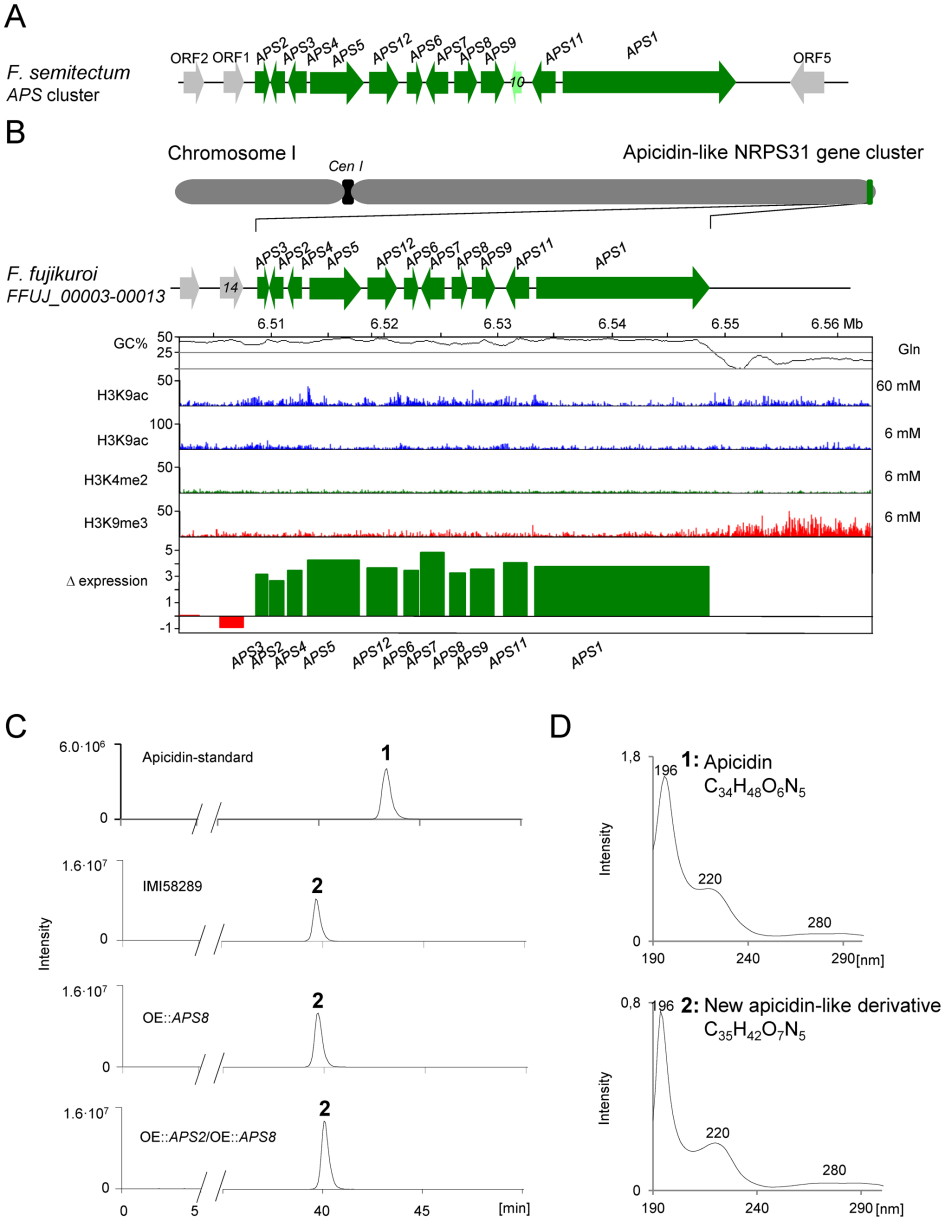
Location of the NRPS/APS biosynthetic gene cluster on *F. fujikuroi* chromosome I, levels of histone modifications and gene expression within and flanking the cluster, and production of metabolites following overexpression of cluster genes *APS2* and *APS8*. **A**: Synteny between the apicidin gene cluster in *F. semitectum* [105] and the apicidin-like gene cluster in *F. fujikuroi*. **B**: Histone modifications and gene expression in and flanking the cluster. Histone marks are described in the legend to Figure 3. Expression data were derived from microarray experiments in low and high nitrogen and are plotted as the changes in log_2_ expression values in high-nitrogen medium compared to low-nitrogen medium. H3K9ac and gene expression are overall correlated, as both are increased under high nitrogen conditions. In some genes increased H3K4me2 was observed, also suggesting transcription. **C**: Chemical analysis of the product of the unique PKS19 gene cluster. The traces show the extracted ion chromatograms for [C_34_H_48_O_6_N_5_+H]^+^ (first line) and [C_35_H_42_O_7_N_5_+H]^+^ (second to fourth line) determined by HPLC-FTMS of an apicidin standard (first line) and of culture fluids from *F. fujikuroi* IMI58289, OE::APS8 and the OE::APS2/OE::APS8 mutant. **D**: UV spectra of apicidin and the apicidin-like compound. The similar spectra suggest a structural similarity.

Genome sequence comparisons revealed that the *F. fujikuroi* PKS19 and NRPS31 clusters are not present in the other *Fusarium* species examined (Figures 8 and 9). Thus, we conducted additional analyses to further define these clusters and to obtain evidence for the corresponding SM metabolites. BLAST analysis revealed that NRPS31 is closely related to the NRPS APS1 (66–71% identical) in *F. semitectum* (Table S4). In *F. semitectum*, the *APS1* gene is part of a 12-gene cluster responsible for synthesis of apicidin, a histone deacetylase inhibitor with antiparasitic activity [105], [106]. In *F. fujikuroi*, the organization of genes adjacent to *NRPS31* (Table S4) is largely syntenic to the organization of genes in the APS cluster in *F. semitectum* and will be referred to hereafter as *APS1* to *APS9*, *APS11* and *APS12*, respectively (Figure 13A). Exceptions to this are absence of an *APS10* homologue and inversion of the order and orientation of *APS2* and *APS3* in *F. fujikuroi* (Figure 13A). To our knowledge, apicidin production has not been reported in *F. fujikuroi*. In addition, chemical analyses performed during the course of the current study did not provide evidence for production of apicidin by *F. fujikuroi*.

Microarray analysis revealed expression of the *F. fujikuroi* APS cluster homologue under the acidic high-nitrogen condition (Figure 13B; Table 4; Table S7). In accordance with the microarray data, APS cluster genes were slightly enriched for H3K9ac in the high-nitrogen condition, while almost no H3K9ac was present in the acidic low-nitrogen condition. Only the *APS5* homologue was enriched for H3K4me2 in the high-nitrogen condition, and no H3K4me2 enrichment occurred in the low-nitrogen condition (Figure 13B). The APS cluster did not exhibit enrichment of H3K9me3, which can be indicative of gene silencing, under the low-nitrogen (non-inducing) condition. However, there was enrichment of the AT-rich region immediately downstream of *APS1* in low nitrogen. Nevertheless, microarray data and H3K9ac enrichment of the cluster region provide evidence that the acidic high-nitrogen condition can activate expression of the APS cluster homologue in *F. fujikuroi*.

### Functional analysis of *F. fujikuroi* novel gene clusters

In order to determine the metabolites synthesized by the two novel clusters, we employed our understanding of SM synthesis and a variety of targeted gene expression and natural product characterization approaches. First, a more detailed analysis of sequences of the *F. fujikuroi APS* homologues indicated that the *APS8* gene might be defective because it encodes a protein (Aps8) that is 125 amino acids shorter at the N-terminus compared to the Aps8 protein of *F. semitectum*. To determine whether the potentially defective *APS8* gene was responsible for lack of apicidin production in *F. fujikuroi*, we generated a strain of *F. fujikuroi* that overexpressed the *F. semitectum APS8* homologue. However, overexpression of the *F. semitectum APS8* did not induce apicidin production when the strain was grown in the acidic high-nitrogen condition (Figure 13C). In *F. semitectum*, overexpression of the *APS2* gene, which encodes a positive-acting Zn(2)C6 transcription factor, enhanced apicidin production [106]. Therefore, we generated an additional strain of *F. fujikuroi* that overexpressed both *F. fujikuroi APS2* and *F. semitectum APS8*. HPLC-FTMS analysis of this mutant revealed that it was significantly enhanced, compared to the wild type, in production of a metabolite with the molecular formula C_35_H_42_O_7_N_5_, which is similar to C_34_H_48_O_6_N_5_, the molecular formula of apicidin (Figure 13C). The possibility that the C_35_H_42_O_7_N_5_ metabolite (compound 2 in Figure 13C and 13D) is structurally similar to apicidin is also supported by the similar UV spectra of the two metabolites (Figure 13D). Hydrolysis of compound **2** was carried out to determine its amino acid composition. Apicidin consists of the four amino acids (S)-*N*-methoxy-tryptophan, (R)-pipecolic acid, (S)-2-amino-8-oxodecanoic acid and (S)-isoleucine. In contrast, compound **2** is composed of three amino acids, (S)-*N*-methoxy-tryptophan, (R)-pipecolic acid, phenylalanine and an fourth amino acid-like material with a molecular formula of C_8_H_15_NO_4_. Thus, two of the four amino acids that make up compound **2** differ from those that make up apicidin. These differences in amino acid composition could result from differences in substrate specificities of the *F. fujikuroi* and *F. semitectum* APS1 homologues that catalyze condensation of different amino acids during biosynthesis. The absence of an *APS10* homologue in *F. fujikuroi* could also contribute to the structural difference if it is responsible for modification of an amino acid.

**Figure 14:**
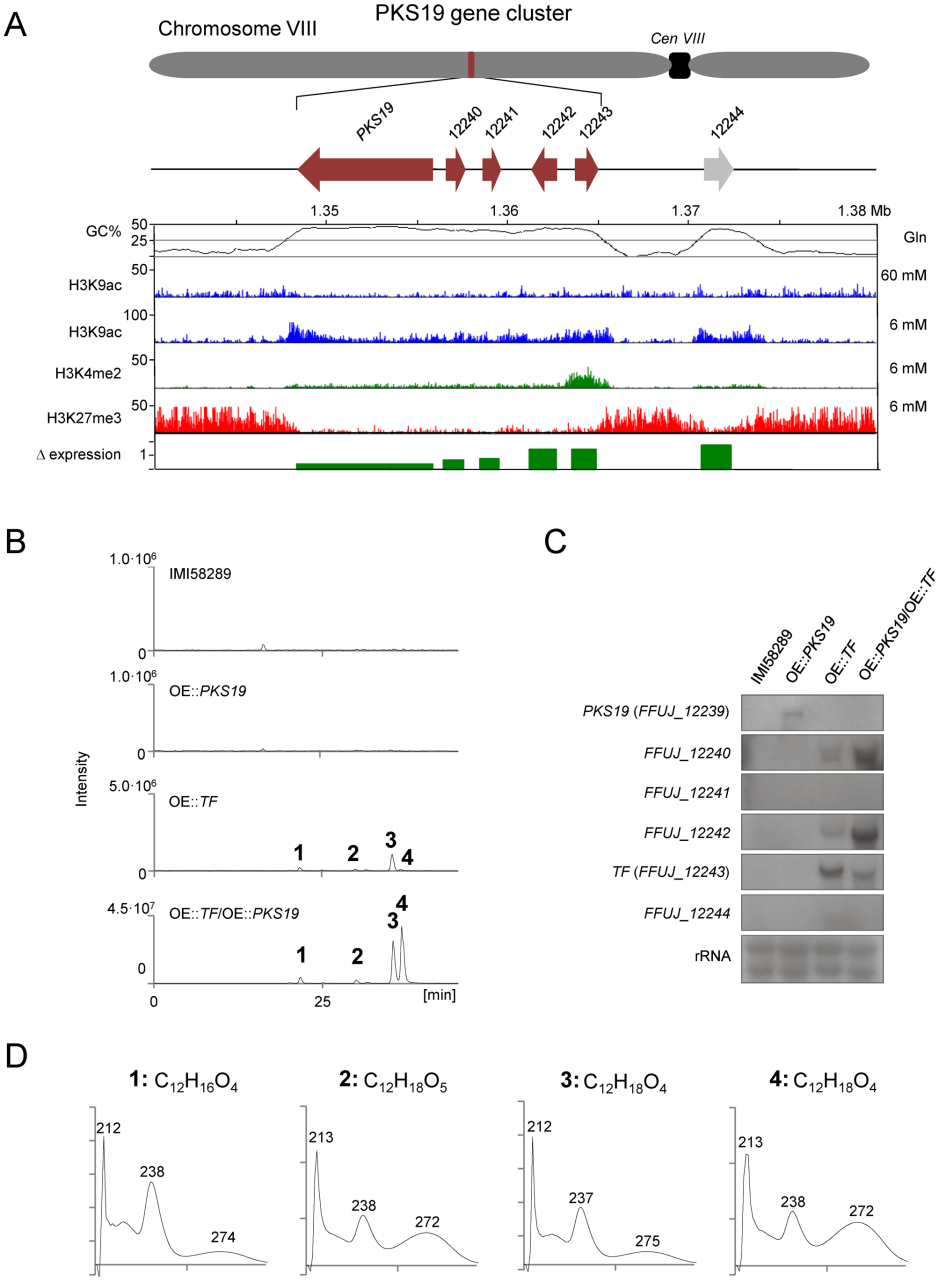
Functional characterization of the PKS19 cluster, a putative polyketide biosynthetic gene cluster that is unique to *F. fujikuroi*. (A) Position and organization of the PKS19 gene cluster on *F. fujikuroi* chromosome VIII, GC content, distribution of active histone marks, and gene expression in the PKS19 cluster. Histone marks are described in the legend for Figure 3. Expression data are from microarray analysis of wild-type *F. fujikuroi* (strain IMI58289). Values are log_2_ change in expression in a high versus low-nitrogen medium. H3K9ac and gene expression are correlated, as both are increased in the high-nitrogen medium. Some genes exhibited increased levels of H3K4me2 in the high-nitrogen medium, which also suggests transcription. (B) Chemical analysis of the SM product(s) of the PKS19 cluster. The traces show the combined extracted ion chromatograms for metabolites with molecular formulas [C_12_H_16_O_4_+H]^+^, [C_12_H_18_O_5_+H]^+^ and [C_12_H_18_O_4_+H]^+^ determined by HPLC-FTMS of culture fluids from *F. fujikuroi* strains: IMI58289, wild-type strain; OE::TF, a strain over-expressing the transcription factor encoded by FFUJ_12242; OE::PKS19, a strain over-expressing the PKS19 gene FFUJ_12239; and OE::TF/OE::PKS19, a strain over-expressing both FFUJ-12242 and FFUJ_12239. (C) Northern blot analysis of PKS19 cluster genes in strains IMI58289 (WT), OE::TF, OE::PKS19 and OE::TF/OE::PKS19. (D) UV spectra of metabolites corresponding to peaks 1 through 4 from chromatograms shown in C. The similar spectra of the metabolites suggest structural similarity.

As noted above, the minimal PKS19 cluster consists of genes encoding a PKS, a DltD-domain protein, and a ToxD-like protein. However, based on proximity, the cluster may include three additional genes; one that encodes a putative membrane protein (FFUJ_12242), a second that encodes a putative Zn(2)C6 transcription factor (FFUJ_12243), and the third that encodes a putative P450 monooxygenase (FFUJ_12244) (Figures 8, 14). PKS19 is a reducing PKS, a subclass of PKSs that typically catalyze synthesis of polyketides with a fully reduced carbon skeleton. Thus, it is unlikely that the SM product of the PKS19 cluster is a polycyclic aromatic compound.

Microarray analysis indicated that two and three of the six PKS19 cluster genes were expressed in alkaline and acidic high-nitrogen conditions, respectively, albeit at relatively low levels (Table 4; Table S7). The PKS19 cluster exhibited minimal H3K9ac under high nitrogen and only slightly more under low-nitrogen conditions, which is consistent with the overall low expression levels observed for the PKS19 cluster by microarray analysis (Figure 14A). Only the transcription factor gene FFUJ_12243 was enriched for H3K4me2. The AT-rich regions flanking the ends of the cluster and between FFUJ_12243 and FFUJ_12244 were enriched in H3K9me3, which is associated with heterochromatic/gene silencing.

To enhance expression of the PKS19 cluster genes and potentially induce production of the corresponding SM product(s), we generated strains of *F. fujikuroi* that overexpressed *PKS19* (OE::PKS19) and/or the putative Zn(2)C6 transcription factor gene (FFUJ_12243) Overexpression of *PKS19* had no effect on expression of the cluster nor did it noticeably alter the metabolic profile of *F. fujikuroi*. In contrast, overexpression of the putative transcription factor gene (FFUJ_12243) enhanced expression of *PKS19* (FFUJ_12239), FFUJ_12240, FFUJ_12242, and FFUJ_12243, but not FFUJ_12241 or FFUJ_12244 (Figure 14C). Analysis of culture extracts of the double overexpression strain (OE::PKS19/OE::FFUJ_12243) led to identification of four metabolites (Figure 14, compounds **1**, **2**, **3** and **4**) that were not produced by the *F. fujikuroi* progenitor strain. Compound **1** and **2** have the molecular formulas C_12_H_16_O_4_ and C_12_H_18_O_5_ respectively, while compounds **3** and **4** have the molecular formula C_12_H_18_O_4_ (Figure 14B, D). The similar UV spectra of compounds **1**–**4** observed combined with their similar molecular formula suggest that the chemical structures of the compounds are similar. For example, C_12_H_18_O_5_ and C_12_H_16_O_4_ differ by 2 hydrogen atoms and 1 oxygen atom which could be due to the loss of H_2_O from the former. Although overexpression of *PKS19* alone did not result in production of compounds **1**–**4**, simultaneous overexpression of both *PKS19* and FFUJ_12243 resulted in production of ∼10-fold more of the compounds than overexpression of the transcription factor-encoding gene (FFUJ_12243) alone. Elucidation of the chemical structures of compounds **1–4** is in progress.

This is the first example in fungi where simultaneous overexpression of transcription factor- and PKS-encoding genes of the same SM cluster were performed and resulted in the production of a metabolite(s). The significantly enhanced production of compounds **1**–**4** via this simultaneous overexpression indicates that this strategy might be an effective tool to induce production of other fungal SMs from otherwise silent SM gene clusters (e.g. the PKS17/PKS18 cluster).

**Figure 15:**
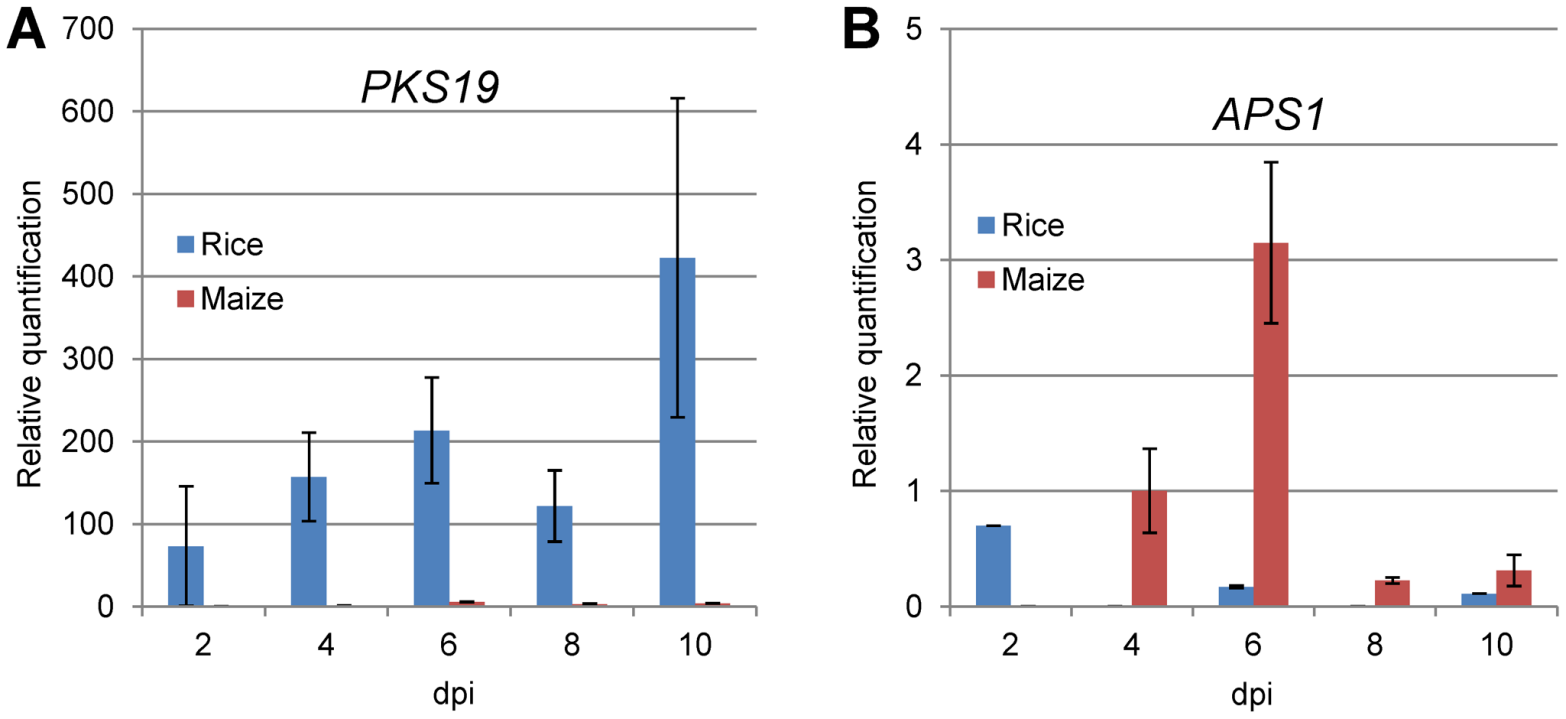
Relative expression of *PKS19* and *APS1* genes in maize and rice roots. Maize and rice roots were infected with *Fusarium fujikuroi* spores, and every 2 days RNA was isolated from three or five plants and used in real time PCR analysis. The expression levels were obtained using the delta-delta Ct and were normalized against three reference genes encoding a related actin (FFUJ_05652), a GDP-mannose transporter (FFUJ_07710) and ubiquitin (FFUJ_08398). The expression levels of *PKS19* (A) at 4 days in maize was arbitrarily set as 1, and all other expression levels were reported relative to it. In the case of the *APS1* gene (B), the expression levels of this gene at 4 days in maize was arbitrarily set as 1, and all other expression levels were reported relative to it.

To determine whether the NRPS31 and PKS19 clusters are expressed *in planta*, we assessed levels of *APS1* and *PKS19* transcripts in *F. fujikuroi*-infected maize and rice roots by qPCR (Figure 15). Surprisingly, expression of both genes was clearly plant-specific. The transcript levels of *APS1* were generally significantly higher on maize than on rice while transcript levels for *PKS19* were always higher on rice and almost not detectable on maize (Figure 15). The high levels for *PKS19* transcripts detected were in sharp contrast to the failure to detect transcription after growth in liquid culture. These differences in expression *in vitro* and *in planta* on one hand, and in the two plants on the other hand, suggest that the SM products of the PKS19 gene cluster may play a specific role in the rice-*F. fujikuroi* interaction. Thus, together with GAs the new PKS19-derived products may contribute to the evolutionary success of *F. fujikuroi* as a rice pathogen.

In conclusion, our study provides a high-quality genome sequence of *F. fujikuroi* that was assembled into only 12 scaffolds corresponding to the 12 chromosomes of the fungus. The availability of the genome sequences of six other *Fusarium* species (among them are two other newly sequenced *Fusarium* species) facilitated the most comprehensive genome-wide analysis to date of SM biosynthetic gene clusters in fungi. This, in combination with a broad spectrum of experimental approaches on the level of chromatin, transcription, proteome and chemical product analyses under different nitrogen and pH conditions provided new insights into the complex network of gene regulation. We show that most of the gene clusters are differentially regulated by nitrogen availability and pH and that there is a correlation between activating histone marks (H3K9ac and H3K4me2), high levels of mRNA and protein production for some (e.g. the GA cluster), but not all gene clusters.

As a highlight, we present the identification of two new gene clusters (PKS19, NRPS31) that are thus far unique for *F. fujikuroi*. Transcriptional engineering of these gene clusters by overexpression of the pathway-specific transcription factors and/or the key enzymes enabled the identification of the respective products for both novel gene clusters by HPLC-FTMS analyses. Furthermore, *in planta* expression studies revealed expression of the otherwise silent PKS19 gene cluster specifically in rice suggesting a specific role for the *PKS19*-derived product during rice infection beside GAs. Furthermore, we set out to find *bona fide* biological functions for gibberellins during rice root infection, which has been unclear so far even though production of the compounds by the fungus was discovered a long time ago. We also discovered that the GA gene cluster is present in some related *Fusarium* species but that GA biosynthesis is restricted to *F. fujikuroi*.

The results from our study highlight the importance of genome sequencing in combination with multiple comprehensive (“omics”) analyses to gain insights into the potential for SM production, the various levels of regulation that govern their biosynthesis, and the impact on plant-fungus interaction.

## Material and Methods

### Fungal and bacterial strains

Strain IMI58289 (Commonwealth Mycological Institute, Kew, United Kingdom) is a GA-producing wild-type (WT) strain of *F. fujikuroi* used unless otherwise indicated. Strains m567 and m570, provided by the Fungal Stock Center at the University Jena, Germany, C-1993 and C-1995, provided by J.F. Leslie, Kansas State University, U.S.A., E289, E292 and E325 (isolated from infected rice), provided by Stefano Tonti, University Bologna, Italy, MRC2276 and MRC2388 (isolated from infected rice), provided by W.C.A. Gelderblom, South Africa, and NCIM1100, provided by the National Collection of Industrial Microorganisms in Pune, India, were used for comparative PCR approaches to confirm the presence or absence of chromosome XII and portions of chromosome IV. The following strains were used to compare the expression of SM biosynthetic genes and SM production: *F. circinatum* Fsp34, provided by B. Wingfield, University of Pretoria, South Africa, *F. mangiferae* MRC7560, provided by S. Freeman, ARO, The Volcani Center, Bet Dagan, Israel, *F. proliferatum* ET1, provided by Elena Tsavkelova, Moscow State University [65], *F. verticillioides* M-3125, provided by D. Brown, U. S. Department of Agriculture, U.S.A., *F. oxysporum* 4815, provided by A. Di Pietro, Universidad de Córdoba, Spain. *F. oxysporum* HDV247, PHW815, PHW808 and FOSC 3-a, provided by D. Geiser, Pennsylvania State University, U.S.A. and *F. oxysporum* 5176a, provided by M. Rep, University of Amsterdam, The Netherlands. GA-defective mutant strain *F. fujikuroi* SG139 was provided by J. Avalos, University of Sevilla. *Escherichia coli* strain Top10 F′ (Invitrogen, Groningen, The Netherlands) was used for plasmid propagation.

### Cultivation methods

For RNA isolation, microarray analyses, SM production and chromatin immunoprecipitation experiments (ChIP), strains were first cultivated for 3 days in 300-ml Erlenmeyer flasks with 100 ml Darken medium [107] on a rotary shaker at 180 rpm at 28°C. 500 µl of this culture were then used to inoculate 100 ml of ICI (Imperial Chemical Industries, UK) media [108] containing either 6 mM glutamine, 60 mM glutamine, 6 mM sodium nitrate or 120 mM sodium nitrate. Growth proceeded for 3 days on a rotary shaker at 28°C in the dark. The mycelia were harvested, washed with deionized water, and flash frozen with liquid nitrogen prior to lyophilization. For SM production, growth proceeded for 6 days on a rotary shaker at 28°C in the dark.

### Nucleic acid isolation

Fungal DNA or RNA was prepared by first grinding lyophilized mycelium or maize or rice plant roots into a fine powder with a mortar and pestle followed by suspension in corresponding extraction buffers. Genomic DNA was isolated as described [109]. For isolation of highly pure DNA for genome sequencing, DNA was re-precipitated with 1/10 volume of 3 M sodium acetate (NaOAc) (pH 5.2) plus 3 volumes of cold 96% ethanol in glass tubes. The precipitating DNA was then wound around a glass pipette, washed in 70% ethanol and dissolved in TE buffer. Plasmid DNA from *E. coli* was extracted using the Gene-JETTM Plasmid Miniprep Kit (Fermentas GmbH, St Leon-Rot, Germany) and sequenced using the BigDye Terminator v3.1 Cycle Sequencing Kit and the ABI PRISM 3730 Genetic Analyzer (Applied Biosystems, Foster City, CA, USA) according the manufacturer's instructions. Total RNA was isolated from mycelium from liquid cultures or from infected maize or rice roots using the RNAgents total RNA isolation kit (Promega GmbH, Mannheim, Germany).

### Plasmid construction

The pOE-HPH and pOE-NAT vectors were generated by first PCR amplification of the *gpd*-promoter region from the pIG1783 vector [110] using the primer pair gpd-yeast-for and gpd-yeast-rev. Fragments of DNA including the hygromycin resistance cassette were generated by PCR from plasmid pCSN44 [111] using the primer pair hph-OE-Prom and hph-OE-Term while DNA including the nourseothricin resistance cassette was generated by PCR from plasmid pNR1 [111], [112] using the primer pair nat-OE-Prom and nat-OE-Term. For pOE-HPH, the *gpd* and the hygromycin resistance cassette fragments were cloned into the *Eco*RI and *Xho*I restricted pRS426 [113]. A similar approach was used for the creation of the pOE-NAT vector, using the nourseothricin resistance cassette instead of the hygromycin resistance cassette. Fragments of DNA containing genes of interest were then cloned into the pOE vectors using the unique *Hin*dIII restriction site introduced in the primer sequences (underlined). Specifically, *APS8*, amplified from genomic DNA of *Fusarium semitectum* using the primer pair aps8-OE-for and aps8-OE-rev, was cloned downstream of the *gpd* promoter in the restricted pOE-NAT vector via yeast recombination cloning [114], [115]. The resulting pOE::*APS8* vector was transformed into wild type *F. fujikuroi*. A fragment of DNA containing *APS2* from wild type *F. fujikuroi* was generated with the primer pair aps2-OE-for and aps2-OE-rev and cloned into the pOE-HPH vector via yeast recombination cloning. The resulting pOE::*APS2* vector was then transformed into the OE:*APS8* strain to create the double *APS8*/*APS2* overexpression strain. A similar strategy was used to create the PKS19 cluster transcription factor (FFUJ_12243) and the PKS19 (FFUJ_12239) over expression vectors. The amplicon containing FFUJ_12243 was generated from wild type *F. fujikuroi* DNA with the primer pair PKS19TF-OE-for and PKS19TF-OE-rev and the amplicon containing FFUJ_12239 was generated with the primer pair PKS19-OE-for and PKS19-OE-rev.

### Northern analyses

Northern blot hybridizations were accomplished essentially as described by [116]. Total RNA (15 mg) were separated by electrophoresis on a 1% (w/v) agarose gel containing formaldehyde, transferred to Hybond-N+ membranes and then hybridized to radioactively labeled DNA. The agarose gel was illuminated with UV light (256 nm), against the background of a silica thin-layer chromatography plate (Schleicher und Schuell, Dassel, Germany) to visualize 18S and 28S rRNA bands. Labeled bands were visualized by exposure to photography film.

### PCR and quantitative Real Time PCR

PCR mixtures contained 25 ng of template DNA, 50 ng of each primer (Table S8), 0.2 mM deoxynucleoside triphosphates, and 1 U of Biotherm Taq polymerase (Genecraft, Lüdinghausen, Germany). Reverse transcription-PCR (RT-PCR) was performed using the Superscript II (Invitrogen, Groningen, The Netherlands) and 1.5 µg of total RNA as the template, according to the manufacturer's instructions. Quantitative PCR (qPCR) was performed using iTaq Universal SYBR Green Supermix (BioRad) and Superscript II cDNA as template, in a Biorad thermocycler iTaq. In all cases the qPCR efficiency was between 90–110% and the annealing temperature was 56–60°C. Every sample was run twice and the results were calculated according to the delta-delta-Ct [117]. As reference genes, related actin gene (primers FRACRTPCRFW and FRACRTPCRRV), GDP-mannose transporter (primers FGMTRTPCRFW and FGMTRTPCRRV) and ubiquitin gene (primers FUBRTPCRFW and FUBRTPCRRV) were used. The following primers were used for amplification of the indicated genes: FFUM1RTPCRFW2 and FFUM1RTPCRRV2 for *FUM1*, FCPSKSRTPCRFW2 and FCPSCSRTPCRRV2 for *CPS/KS*, FAPS1RTPCRFW and FAPS1RTPCRRV for *APS1*, and FPKS19RTPCRFW and PKS19RTPCRRV for *PKS19*.

### Transformations

Transformation of *F. fujikuroi* protoplasts was carried out according to [11]. Regeneration of transformed protoplasts was performed for 4–5 days at 28°C in a regeneration medium (RM) (0.7 M sucrose, 0.05% yeast extract) containing either 100 µg/ml nourseothricin (Werner-Bioagents, Jena, Germany), 100 µg/ml hygromycin (Calbiochem, Darmstadt, Germany) or 100 µg/ml geneticin (Invitrogen Life Technologies, Darmstadt, Germany). Subsequently, purification to homokaryons of putative transformant by single spore isolation was carried out. Integration events were confirmed by diagnostic PCR using specific primers as indicated (Table S8).

Strains IMI58289-DsRed and SG139-DsRed were generated by transformation of the corresponding protoplasts with pNDN-ODT coding for DsRed and a nourseothricin resistance cassette [118]. Integration of the plasmid was verified by diagnostic PCR using primers POliC_seq_F1 and DsRed_seq_R1.

### Virulence assay

Infection of rice plants was performed according to [14]. Germinated rice seeds were co-cultivated with *F. fujikuroi* IMI58289-DsRed, SG139-DsRed or *F. verticillioides* M-3125 respectively. The cultures were incubated for 10 d at 28°C with a humidity of 80% in 12 h light and 12 h dark conditions. For microscopic analyses the rice roots were cleaned with sterile water and cut from the plant. Quantification of penetration events in seven infected roots for each strain was performed by visual inspection using fluorescence microscopy. For gene expression assays, spores (10^4^/ml) of *F. fujikuroi* or *F. verticillioides* were used instead of agar plugs.

Infection of maize plants was performed according to [119]. Germinated maize seeds were cultivated with the same three fungal strains but in cycles of 30°C with light for 14 hours, and 20°C in darkness for 10 hours. Roots were collected from three plants at two day intervals, cleaned with sterile water, flash frozen and lyophilized.

### Genome sequencing and mapping

High throughput next generation sequencing using the Roche 454 GS-FLX system and the Titanium series chemistry was performed by Eurofins MWG Operon, Germany. The ultra high throughput sequencing process was carried out by shot gun library sequencing with 350–450 bp read length, reaching in average of 20-fold coverage (altogether approximately 900 million sequenced bases). The genome was assembled with the help of Long-Tag paired end sequences (3 kb and 8 kb libraries), into 18 scaffolds (size : >8.3 kb; N50: 4.2 Mb) containing 73 contigs with an average read coverage of 18.96 and a genome size of 43.9 Mb. The 13 largest scaffolds were mapped to 11 orthologous *F. verticillioides* chromosomes [22] using Mauve [120] and MUMmer [121]. Two gaps between four scaffolds were closed by PCR sequencing and reassessment of low coverage reads. The final 12 largest scaffolds match well to the predicted 12 chromosomes for *F. fujikuroi* [39].

#### Gene models

Gene models for *F. fujikuroi* were generated by three different prediction programs: 1) Fgenesh [122] with different matrices (trained on *Aspergillus nidulans*, *Neurospora crassa* and a mixed matrix based on different species); 2) GeneMark-ES [123] and 3) Augustus [124] with *Fusarium* ESTs as training sets. Annotation was aided by Blastx hits between the *F. fujikuroi* genome and protein sequences from *F. verticillioides*, *F. oxysporum* and *F. graminearum*, respectively. In addition, Swiss-Prot was also blasted against the scaffolds to uncover gene annotation gaps. The different gene structures and evidences were displayed in GBrowse [125], allowing manual validation of all coding sequences (CDSs). The best fitting model per locus was selected manually and gene structures were adjusted by manually splitting them or redefining exon-intron boundaries based on EST data where necessary. The final call set comprises 14,813 protein coding genes. In addition, 232 tRNA-encoding genes are predicted for *F. fujikuroi* using tRNAscan-SE [126]. The predicted protein set was searched for highly conserved single (low) copy genes to assess the completeness of the sequence dataset. Ortholog genes to all 246 single copy genes were identified by blastp comparisons (eVal: 10^−3^) against the single-copy families from all 21 species available from the FunyBASE [127]. Additionally, all of the 248 core-genes commonly present in higher eukaryotes (CEGs) could be identified by Blastp comparisons (eVal: 10^−3^) [40].

### Identification of repetitive DNA elements

Interspersed repeat elements were identified using RepeatScout with default parameters [128]. Repeats with length less than 50 bp were removed. Low complexity and simple sequence repeats were determined by NSEG [129] and Tandem-Repeat-Finder [130], which are part of the RepeatScout procedure. We considered the RepBase database to scan for known repetitive and transposable elements [50]. New putative transposable elements were identified and characterized in selecting interspersed repeats that contain predicted ORFs with transposase, reverse transcriptases or integrase InterPro domains. Additionally we used LTR_FINDER [131] for the identification of long terminal repeats. For repeat mapping and masking RepeatMasker was used [132].

### Annotation of predicted open reading frames and comparative analysis

The 14,813 protein coding genes of *F. fujikuroi* were analyzed and functionally annotated using the Pedant system [133], accessible at http://pedant.helmholtz-muenchen.de/genomes.jsp?category=fungal. The genome and annotation was submitted to the European Nucleotide Archive (ENA, http://www.ebi.ac.uk/ena/data/view/HF679023-HF679034).

### Genome comparison

The genomes of *F. verticillioides* and *F. oxysporum* were retrieved from http://www.broadinstitute.org/annotation/genome/fusarium_group/MultiHome.html [22]. The genome of *F. graminearum* and annotation version 3.2 was retrieved from http://mips.helmholtz-muenchen.de/genre/proj/FGDB/ [134]. Brenda Wingfield, University of Pretoria, South Africa provided access to genome sequence of *F. circinatum* [35]. Stanley Freeman, The Hebrew University of Jerusalem, Israel provided access to genome sequence of *F. mangiferae*. All genomes were analyzed using the Pedant system [133] to allow comparative feature analysis which includes computation of the Similarity Matrix of Proteins (SIMAP) [42]. The SIMAP database provides a comprehensive calculation of protein sequence similarities/identities, sequence-based features and protein function predictions. Amino acid identities of homologous stretches are multiplied by the length of the homologous region and divided by the length of the whole protein. Proteins with a bidirectional best hit between two species were used to determine collinear regions using the tool Orthocluster [134]. A collinear block was defined as three consecutive, orthologous genes, allowing one missing or additional gene in between. Whole genome nucleotide alignments were performed using MUMmer [121] with a cluster length of exact matches of at least 100 nt and at most 500 nt mismatches between two exact matches.

### *In silico* identification of secondary metabolite clusters

To scan the genome for SM clusters the InterPro scan results of the Pedant analysis as well as SMURF analysis [83] were used. Proteins containing characteristic domains of signature enzymes were taken as a seed for a putative local gene cluster. Each cluster seed was then extended by determining if neighboring genes encoding typical SM enzymes like P450 monooxygenases, methyltransferases, monooxygenases as well as transporter proteins or transcription factors. Each cluster of genes was manually validated by adjusting to previously published data and comparative analysis to homologous clusters in other *Fusarium*.

### Prediction of secreted proteins

We computed secreted proteins in a pipeline approach using a combination of five methods. Therefore we selected a set of putative secreted proteins using SecretomeP [130] and a cutoff score of 0.6. Out of this set we excluded proteins with mitochondrial target using TargetP [135]. We also filtered on proteins with a TargetP RC-score less than four. A prediction of extracellular target compartments has been done with Wolfpsort [136]. To exclude extracellular, membrane bound proteins we utilized TMHMM [137] for transmembrane domain prediction. The SignalP program [138] was used to differentiate between classically and non-classically secreted proteins. Here we assume that proteins having an s-score greater than 0.5 contain a signal peptide and therefore are secreted classically.

### Phylogeny

Calculations of phylogenetic relationships between eight *Fusarium* species are based on the nucleotide sequences of 28 genes. All genes are involved in energetic processes according to FunCat database [43] and show at least 90% identity on protein level to their respective orthologs of the other species. Alignments of the orthologs have been calculated with Mafft [139] using default parameters. Afterwards all alignments were concatenated and columns containing gaps were removed. For phylogenetic tree calculation we used PhyML, an implementation of the maximum likelihood algorithm [140]. We chose the HKY85 substitution model and determined tree support in performing the bootstrap test with 1000 replicates. The gene codes of the 28 genes used for the calculation are: FFUJ_01340, FFUJ_01475, FFUJ_02452, FFUJ_02823, FFUJ_02928, FFUJ_02998, FFUJ_04170, FFUJ_04413, FFUJ_04421, FFUJ_04860, FFUJ_04893, FFUJ_05032, FFUJ_05937, FFUJ_06185, FFUJ_07582, FFUJ_08292, FFUJ_08315, FFUJ_08319, FFUJ_08541, FFUJ_08584, FFUJ_09575, FFUJ_09776, FFUJ_09852, FFUJ_13056, FFUJ_13472, FFUJ_13565, FFUJ_13760, FFUJ_13774. For phylogenetic analysis of SM genes, predicted amino acid sequences were aligned with the ClustalW+ alignment tool using the BLOSUM scoring matrix. Aligned sequences were then subjected to maximum parsimony analysis as implemented in the program PAUP version 4.0b10-Unix. GA biosynthetic gene sequences were subjected to maximum likelihood analysis as implemented in MEGA5 [141]. Statistical support for branches within phylogenetic trees was determined by bootstrap analysis with 1000 (maximum parsimony) or 500 (maximum likelihood) pseudoreplications.

### Microarray design

Based on the de novo sequence of *F. fujikuroi* and the gene annotation, in consultation with Roche-NimbleGen Systems, Inc. (Madison, WI), we designed a 12-plex microarray with 16522 probe sets (array design 110407_UG_Ffujikuroi_expr_HX12). Each probe set included nine perfectly matching oligonucleotide probes. The microarray data are available from the NCBI Gene Expression Omnibus (GEO) under the series accession numbers GSM1070182 - GSM1070196, listed at http://www.ncbi.nlm.nih.gov/geo/query/acc.cgi?acc=GSE43745. Hybridization of microarrays was done at Arrows Biomedical (Münster, Germany) essentially according to manufactures protocols.

### Analysis of expression data

Microarray tif images were processed to numeric raw data using NimbleScan v2.6 software. The preprocessing steps including background correction, normalization and summarization of probe intensities, were performed employing the oligo R package [142] and the RMA algorithm [143]. We used the limma R package [144] to identify differentially expressed genes by fitting linear models for each gene and computing moderated t-statistics using the empirical Bayes method. The resulting p-values were corrected for multiple testing by using the Benjamini-Hochberg procedure [145]. We regard genes having a log2 fold-change greater than one and corrected p-value (FDR) less than 0.05 between two conditions as differentially expressed. The MIPS Functional Catalogue (FunCat, http://mips.helmholtz-muenchen.de/proj/funcatDB/) was used to assign specific genes to specific biological processes and the MIPS single enrichment tool was used to identify categories whose members were over-represented in the expression analysis compared to the genes present on the microarray [43].

### ChIP sequencing

For chromatin immunoprecipitation (ChIP) experiments, the mycelium of wild type was pre-cultivated for 72 hours in Darken medium. 500 µl of the pre-culture was then used for the inoculation of 100 ml ICI medium with either 6 mM glutamine (for nitrogen starvation conditions) or 60 mM glutamine (for nitrogen excessive condition). After three days the mycelium was crosslinked with 1% formaldehyde and incubated for 30 minutes. Then 5 ml glycine (5 M) was added to the culture for quenching and the mycelium was harvested by filtration. About 150 mg of fresh mycelium was used for subsequent ChIP [47], [146]. The antibodies used were H3K4me2 (Millipore, 07-030), H3K9me3 (Active Motif, 39161) and H3K9Ac (Active Motif, 39137). Sequencing libraries were prepared as described [147]. Single-end 36–50 nt sequencing was carried out on an Illumina GAII genome analyzer. Reads with mapped to the *F. fujikuroi* genome with BWA using default conditions [148] and visualized in a gbrowse2 genome browser [149]. Quantification of ChIP-seq reads present in each gene was performed with EpiChIP [47], [146], [150].

The ChIP seq data are available from the NCBI Gene Expression Omnibus (GEO) under the series accession numbers GSM 1122108 - GSM 1122111, listed at http://www.ncbi.nlm.nih.gov/geo/query/acc.cgi?acc=GSE46033.

### Chemical analysis

Chemicals and solvents were purchased from Sigma-Aldrich (Deisenhofen, Germany) or Grüssing GmbH (Filsum, Germany). Gibberellins were obtained from Serva (Heidelberg, Germany), fusaric acid and beauvericin were obtained from Sigma (Deisenhofen, Germany) and apicidin was obtained from AppliChem (Darmstadt, Germany). Fumonisin B_1_, fusarin C, *O*-methylfusarubin and bikaverin were isolated from fungal cultures as previously described [14], [15], [151], [152]. All standards for HPLC analysis were dissolved in methanol/water (1/9, v/v) except for beauvericin which was dissolved in methanol/water 7/3, v/v. The concentration for each chemical ranged between 10 and 100 µg/ml. The volatile *ent*-kaurene released by agar plate cultures were collected by use of a closed loop stripping apparatus (CLSA) and analyzed by GC-MS as previously described [88].

### Sample preparation for SM analysis

The fungal strains were grown in a submerged culture as described. The mycelica were removed from the culture by filtration through Miracloth (Calbiochem, Merck KGaA, Darmstadt, Germany) under vacuum. The culture filtrates were filtrated through syringe filters (RC Membrane, 0.45 µm, 4 mm Syringe Filters non-sterile, PP Housing, Luer/Slip, Phenomenex, Aschaffenburg, Germany) and directly used for analysis. The cultivation of each fungus under each culture condition was performed in duplicate. For beauvericin, 0.1 g of the mycelium were extracted with 2 ml of ethyl acetate, methanol and methylene chloride (3/2/1). After shaking for 2 h, 500 µL of the extract were evaporated at 25°C under a stream of nitrogen and suspended in the same volume methanol/water (7/3, v/v). The sample was sonicated (Bandelin Sonorex RK 100 h, Bandelin Electronicm Berlin, Germany) for 30 min and insoluble materials were removed by centrifugation for 3 min at 1400 g prior to HPLC-FTMS analysis.

### HPLC-FTMS

The HPLC-FTMS system used an HPLC system (Accela LC with Accela Pump 60057-60010 and Accela Autosampler 60057-60020, Thermo Scientific, Dreieich, Germany) coupled to a Fourier transform mass spectrometer with a heated ESI source (LTQ Orbitrap XL, Thermo Scientific, Dreieich, Germany). Ionization was carried out in the positive ionization mode. The parameters were as follows: Capillary temperature 275°C, vaporizer temperature 350°C, sheath gas flow 40 units, auxiliary gas flow 20 units, source voltage 3.5 kV, tube lens 119 V. The software Xcalibur 2.07 SP1 (Thermo Scientific, Dreieich, Germany) was used for data acquisition and analysis. The HPLC column used was a 150 mm×2.00 mm i.d., 5 µm, Gemini C18 with a 4 mm×2 mm Gemini NX C18 guard column (Phenomenex, Aschaffenburg, Germany). The gradient was from 10% A to 100% A in 60 minutes followed by column flushing for 5 minutes at 100% A and equilibration at the starting conditions of 10% A for 10 minutes. The flow rate was 250 µl/min and the injection volume set to 10 µl. For the comparative analysis of different *Fusarium* species, the metabolites were identified by the exact mass of the most intense ion, their isotope pattern and the retention time compared to standard substances (Table S9).

### HPLC-DAD

UV-spectra were generated by HPLC-DAD (Shimadzu LC with DGU-20A3 degasser, LC-10AT VP pumps, SIL-10AF autosampler, CTO-10AS VP column oven, SPD-M20A diode array detector and CBM-20A communication bus module, Shimadzu, Duisburg, Germany). The software LCSolution (Shimadzu, Duisburg, Germany) was used for data acquisition and analysis. The HPLC column used was a 250 mm×4.60 mm i.d., 5 µm, Gemini C18 with a 4 mm×3 mm Gemini C18 guard column (Phenomenex, Aschaffenburg, Germany).

For apicidin analysis, solvent A was methanol (v/v) and solvent B was water (v/v). The gradient was from 10% A to 100% A in 30 minutes followed by column flushing for 5 minutes at 100% A and equilibration of the starting conditions of 10% A for 10 minutes. The flow rate was 1000 µl/min, the injection volume was 30 µl. The retention time of the apicidin-standard was 29.9 min, the retention time of the apicidin-derivative formed by *F. fujikuroi* was 29.8 min.

For analysis of putative PKS19 metabolites, the gradient was from 10% A to 100% A in 60 minutes followed by column flushing for 5 minutes at 100% A and equilibration of the starting conditions of 10% A for 10 minutes. The flow rate was 1000 µl/min, the injection volume was 10 µl. The retention times of the newly identified metabolites were as follows: 1st peak 23.4 min, 2nd peak 29.1 min, 3rd and 4th peak 32.1 and 33.1 min.

### Isolation of putative NRPS31 metabolites

The mutant strain OE::*APS2*/OE::*APS8* was grown in 1 l ICI medium with 60 mM glutamine. The mycelium was removed from the culture by filtration through Miracloth (Calbiochem, Merck KGaA, Darmstadt) under vacuum. The culture filtrate was fractionated on a Strata C18-E (55 µm, 70 Å) 10 g/60 ml SPE column (Phenomenex, Aschaffenburg, Germany) essentially as described by [18]. Briefly, the column was first activated by flushing with 50 ml methanol and 50 ml water under vacuum. The aqueous culture filtrate was applied under vacuum and washed with 100 ml of water. Metabolites were eluted from the column in five fractions of 20%, 40%, 60%, 80% and 100% methanol/water (v/v). Only the 80% fraction contained a novel peak of interest. The solvent was removed on a rotary evaporator (Rotavapor-R, Büchi Labortechnik GmbH, Essen, Germany) and the residue dissolved in about 3 ml of 50% 5% tetrahydrofuran in methanol (v/v) (solvent A) and 50% water (v/v) (solvent B).

### Preparative HPLC-UV

Purification of the new metabolite for further analysis was carried out on a preparative HPLC-UV system (Varian Polaris pumps with Rheodyne manual injection port and Varian ProStar UV-detector, Varian, Europe) using Galaxie 1.9.302.530 (Varian, Europe) software for data acquisition. The column used was a 250×10.0 mm Varian Microsorb 100-5 C18 column with a 10.0 ×10.0 mm Gemini C6-Phenyl guard column. Solvent A was 5% tetrahydrofuran in methanol (v/v), solvent B was water. The flow rate was 3.5 ml/min. The first preparative run was from 50% A to 80% A in 30 minutes followed by equilibration at 50% A for 5 minutes. The UV-detector was set to 254 nm. The collected fractions were tested by HPLC-MS loop analysis (see below) to identify the fractions containing the new metabolite. Due to co-elution of the target peak with fusarins by comparison to standard materials, 3 fractions were collected. The fraction from 18 to about 21 minutes containing only fusarins, the fraction from 21 to 22 minutes containing low amount of the target peak and a high amount of fusarins while the fraction from 22 to 26 minutes contained a high amount of the target peak and a low amount of fusarins. Fractions 2 and 3 were evaporated separately to dryness at the rotary evaporator and dissolved in 70% A. A second preparative HPLC-UV run was carried out on each fraction on the same system with the following change. The samples were loaded and peaks were eluted isocratically at 70% A. The two collected fractions eluting between 8 to 10 minutes contained high amounts of the new metabolite and only minor impurities with fusarins and were combined. The verification of the different fractions was carried out on an API 3200 LC-MS/MS system (Applied Biosystems, Darmstadt, Germany) with a 1200 series HPLC system (Agilent Technologies, Böblingen, Germany). The software Analyst 1.4.2 (Applied Biosystems, Darmstadt, Germany) was used for data acquisition. The HPLC run was at 50% methanol/water (v/v) with a flow rate of 200 µl/min for 2 minutes. 10 µL of the sample were injected and without separation on a column directly infused into the mass spectrometer and analysed after electrospray ionization in the positive ionization mode in a Q1 scan. The ion spray voltage was set to 5500 V, the declustering potential was 50 V and the entrance potential was 3.5 V. Zero grade air was used as nebulizer gas (35 psi), and, heated at 350°C, as turbo gas for solvent drying (45 psi). Nitrogen served as curtain gas (30 psi). The scan range was set to *m/z* 400–700 and the presence of the ions 646 (new metabolite) and 432 (fusarins) was monitored. The quadrupol was set to unit resolution. The fractions containing the metabolite were combined, dried under a stream of nitrogen and the residue was dissolved in 600 µl methanol and stored at −20°C for about 15 minutes until a white precipitate of the metabolite was observed. The precipitate was collected by centrifugation at 20°C, 1100 g for 2 minutes.

### Acid hydrolysis of apicidin and unknown compound

Apicidin and the unknown compound were transferred into separate vials, evaporated to dryness, dissolved in 500 µl 6 M hydrochloric acid and heated on 110°C for 8 h. The acid was then removed at 110°C under a stream of nitrogen and the residue dissolved in 100 µl water and used for HPLC-FTMS analysis. The peptide hydrolyzate was applied to the HPLC-FTMS system with the same column and solvents as described above with the following changes. The gradient was 5% A for 10 minutes followed by column flushing for 5 minutes at 100% A and equilibration at the starting conditions of 5% A for 7 minutes. The flow rate was 250 µl/min and the injection volume set to 10 µl. The composite amino acids for apicidin and the unknown compound were identified based on a comparison of their exact masses to standard amino acids (see Table S10). It should be noted that *N*-methoxy-tryptophan is degraded by acid hydrolysis.

### Proteomics

Protein analyses by LC-MS/MS were conducted as described [153] using samples from 2 independent cell harvests. 15N labeled nitrogen-deficient (6 mM Gln) and ^14^N labeled nitrogen-sufficient (60 mM Gln) cells were mixed on an equal protein basis. Proteins were separated by SDS-PAGE, protein bands were excised and digested tryptically.

Chromatographic separation of peptides was performed using an Ultimate 3000 Nanoflow HPLC system (Dionex). The mobile phases consisted of 5% (v/v) acetonitrile/0.1% (v/v) formic acid in ultrapure water (A) and 80% acetonitrile/0.1% formic acid in ultrapure water (B). The sample (1 µl) was loaded on a trapping column (C18 PepMap 100, 300 µM×5 mm, 5 µm particle size, 100 Å pore size; Thermo Scientific) and desalted for 4 min using eluent A at a flow rate of 25 µl/min. Then the trap column was switched online with the separation column (Acclaim PepMap100 C18, 75 µm×15 cm, 3 µM particle size, 100 Å pore size, Thermo Scientific) and peptides were eluted at a flow rate of 300 nl/min and employing the following gradient profile: 0–50% B over 45 min, 50% B for 5 min, 50–100% B over 1 min, 100% B for 5 min. Afterwards the column was re-equilibrated with 100% A for 10 min. The LC system was coupled via a nanospray source to an LTQ Orbitrap XL mass spectrometer (Thermo Finnigan). MS full scans (*m/z* 375–1800) were acquired in positive ion mode by FT-MS in the Orbitrap at a resolution of 60,000 (FWHM) with internal lock mass calibration on *m/z* 445.12003. The 12 most intense ions were fragmented in the linear ion trap by CID (35% normalized collision energy). Automatic gain control (AGC) was enabled with target values of 5×105 and 5×103 for MS full scans and MS/MS, respectively. One microscan was acquired per MS/MS spectrum and maximum ion trap fill time was 50 ms [154]. Dynamic exclusion was enabled with an exclusion duration of 60 s, repeat count of 1, list size of 500 and exclusion mass width of +/−5 ppm. Unassigned charge states and charged state 1 were rejected. Acquired Thermo Xcalibur raw files were converted to the open mzML format [155] via msconvert (part of Proteowizard, [156].

### Identification of proteins

Proteomics data analyses were performed individually for each biological replicate using Proteomatic [157] as a data processing pipeline tool providing access to the later mentioned tools and pymzML [158] for direct access to mzML files. OMSSA (version 2.1.9, [159]) and X! Tandem (version 2012.10.01.1, [160]) programs were used for protein identification. The parent mass error was set to 20 ppm, the fragment mass error was set to 0.5 Da. Oxidation of M was used a variable modification and a maximum number of 2 missed cleavages was allowed. For OMSSA, a linear precursor charge dependency was set. For X! Tandem, noise suppression was enabled. Separate searches were conducted for ^14^N and ^15^N evaluation, respectively (precursor and product ion search types set to ^14^N or ^15^N).

For the statistical assessment of the quality of PSMs, a target/decoy approach was used. Decoy sequences were built from the *F. fujikuroi* protein database by randomly shuffling tryptic peptides. PSM hits from OMSSA and X! Tandem were filtered (1) using a hit distinctiveness filter as described [161], (2) removing all hits from peptides that occur as a target and as a decoy sequence and (3) protein groups are built for peptides that occur in more than one protein. These steps result in a maximum of one peptide hit per spectrum. Quality (version 2.02) [162] was used for statistical validation of protein identifications with only allowing target hits with a posterior error probability (PEP) less than 0.01. As an additional filtering step, all PSMs with a precursor mass deviation of more than 5 ppm were discarded.

### Quantification of proteins

Significantly identified peptides were used for protein quantification with qTrace separately for both replicates as described [153], except that the 15N labeling approach was used. After protein information was added to the qTrace output, the following filtering steps were conducted as described [153] (1) require MS2 identifications and (2) pick most abundant band. The resulting relative peptide amounts were normalized to the median of all peptide amounts from each replicate. After protein ratios (^15^N/^14^N) were computed by summing up all amounts of proteotypic peptides, results were filtered for at least two peptide/band/charge (PBC) combinations. Hits that did not meet the PBC criteria were required to have a scan count of at least 20. Mean ratios are calculated as the arithmetic mean of the ratios of replicate A and B, SD is the standard deviation of the ratios. If one of the ratios is “0” or “inf”, the resulting ratio mean is the real ratio (not “0” or “inf”), no SD is given. If both ratio are “inf” (or “0”), the resulting ratio mean is “inf” (or “0”), no SD is given. If the ratios are in opposite directions (“>1” and “<1”), no ratio mean and SD are calculated. Heatmaps were created with a custom script in Proteomatic. All Protein ratios were log2-transformed. Correlation between protein ratios and transcriptomics fold changes have been calculated using Hmisc R package (Harrell EF et al., Hmisc: Harrell Miscellaneous, http://cran.r-project.org/web/packages/Hmisc/index.html, 2012). Ratios of proteins that occur in only one experimental condition were set to minimum or maximum ratio to avoid infinity values.

### Fluorescence microscopy

Microscopy was performed using the Axio Imager.M2 (Carl Zeiss MicroImaging GmbH, Jena, Germany). For bright field images differential interference contrast (DIC) was used. Fluorescence of DsRed was detected using filterset 43 HE Cy shift free (excitation BP 550/25, beam splitter FT 570, emission band pass 605/70). Images were captured using equal exposure times and applying the AxioCam MRm (Carl Zeiss MicroImaging GmbH, Jena, Germany). All pictures were processed identically using AxioVision Rel. 4.8 (Carl Zeiss MicroImaging GmbH, Jena, Germany).

### Immunocytology

For immunocytological analysis, 10–14 day old hyphae of *F. fujikuroi* IMI58289 were scraped from CM-plates, fixed for 15 min in freshly prepared 4% formaldehyde solution in phosphate-buffered saline (PBS pH 7.3), transferred on glass slides, covered with coverslips and squashed. After freezing in liquid nitrogen the cover slips were removed and slides were transferred immediately into PBS. After pre-incubation for 30 min in 3% BSA in PBS at 37°C the slides were incubated with the primary antibodies in a humidified chamber. The primary antibodies were used in the following dilutions: 1∶50 anti-H3K9ac (ab10812, Abcam, Cambidge, UK) and 1∶100 anti-H3K9me3 (MAb-146-050, Diagenode, Liège, Belgium). After 1 h incubation at 37°C and washing for 15 min in PBS, the slides were incubated either in Alexa-488 anti mouse IgG or Alexa-555 conjugated anti rabbit IgG secondary antibodies for 1 h at 37°C (1∶100; Molecular Probes, Invitrogen, Karlsruhe, Germany). All preparations were washed in PBS for 15 min, stained with DAPI in mounting solution (antifade) and examined with Zeiss Axioscop fluorescence microscope.

## Supporting Information

Figure S1 **CHEF gel analysis of** ***F. fujikuroi*** **IMI58289.** Previously we performed Southern blot analyses [39] of the CHEF gel with the following gene probes: *CREA* (FFUJ_04790), *hmgR* (FFUJ_04000), *NMR* (FFUJ_02636), *GGS1*(FFUJ_07352), *CPS/KS* (FFUJ_1433), *AREA* (FFUJ_06143), *NIAD* (FFUJ_12277), *CARRA* (FFUJ_11802). Arrows show the position of the hybridization signals, while the red letters for chromosome numbers show the location of these genes on defined chromosomes according to the genome sequence. Chromosome numbers on the right postulate the order of chromosomes I–XII according to the estimated chromosome sizes (kb). * Chromosome I is not shown: it was not separated under the used conditions and stayed in the slots of the gel. ** Chomosome IV is numbered according to the orthologous chromosome IV in *F. verticillioides*. However, this chromosome is significantly smaller in *F. fujikuroi*. Black lines indicate chromosomes that where confirmed by Southern blot hybridization. Dashed lines show postulated positions of the remaining chromosomes according to their size.
(TIFF)

Figure S2 **Variability of chromosomes XII and IV.** **A**: PCR analysis reveals that chromosome XII is not present in all *F. fujikuroi* strains. 1, 2 and 3: PCR products derived from primer pairs designed from the sequence of genes FFUJ_14099, FFUJ_ 14193, and FFUJ_ 14245, respectively. The genes FFUJ_14099, FFUJ_14193, and FFUJ_14245 are located 0.7, 0.4 and 0.08 Mb from the left telomere of chromosome XII. The positions of these genes on chromosome XII are shown in the scheme below. **B**: Chromosome IV is shorter than in *F. verticillioides*. Primers were designed from the sequence of gene FVEG_11841 (4) present on chromosome IV in both *F. fujikuroi* and *F. verticillioides* (PCR bands were amplified in all strains tested) and FVEG_12503 (5), missing in *F. fujikuroi* but present in *F. verticillioides* (PCR bands amplified only in *F. verticillioides*, right lanes). The homolog of FVEG_11841 in *F. fujikuroi*, FFUJ_14790, is located 3.1 Mb from the left telomer. For FVEG_12503 no homolog is present in the *F. fujikuroi* genome. The positions of these genes on chromosome IV are shown in the scheme below.
(TIFF)

Figure S3 **Characterization of** ***F. fujikuroi*** **chromosomes I–XII: variation in GC-content, acetylation and methylation statues of histone H3 and expression under low nitrogen conditions.** For each chromosome, a diagram showing the position of the centromere is shown at the top; below this in descending order are: GC content, location of SM biosynthetic gene clusters, acetylation and methylation states of histone H3 protein, and changes in gene expression. Variation in histone H3 modification status serves as marker for chromosomal regions in which genes are expressed (H3K9ac and H3K4me2) or silent (H3K9me3). “Δ expression up” indicates a more than twofold increase in gene expression during growth of *F. fujikuroi* in a nitrogen-rich medium, whereas “Δ expression down” indicates an at least twofold decrease in gene expression. SM biosynthetic gene cluster locations are indicated by arrows labeled with the PKS, NRPS or TC (DTC means diterpene cyclase; STC means sesquiterpene cyclase) gene in each cluster (see Figure 3, Table 4 and Table S4). *F. fujikuroi* genes (FFUJ locus ID) to the left and right of the predicted centromere on each chromosome was identified and compared to the location of predicted orthologs in *F. verticillioides* (FVEG locus ID). “+” and “−”.
(TIF)

Figure S4 **Maximum likelihood trees of selected GA biosynthetic genes.** **A**: tree generated from Cps/Ks and related diterpene synthases from multiple genera of fungi; **B**: tree generated from concatenated sequences of GA enzymes/genes common to *Fusarium* species, *Claviceps purpurea* and *Sphaceloma manihoticola*. **C**: trees of four GA biosynthetic enzymes/genes (P450-1, P450-2, P450-4 and Cps/Ks) that are common to *Fusarium*, *Claviceps* and *Sphaceloma*; All trees were inferred from alignments generated by Muscle of deduced amino acid sequences, and all gaps in the alignment were removed prior to maximum likelihood analysis. Numbers near branches are bootstrap values based on 500 pseudoreplicates. Only bootstrap values >70 are shown. Muscle and maximum likelihood analyses were conducted using *MEGA* version 5 [163]. Sequences for *F. proliferatum*, and non-*Fusarium* genera other than *Claviceps* were obtained from the NCBI database via BLASTp analysis with *F. fujikuroi* homologues: NCBI accessions for terpene synthases for which accession or strain numbers are not shown in Figure S4A: *A. benhamiae* XP_003013365, *Phaeospheria* sp. L487, O13284; *P. betae*, BAD29971; *S. manihoticola*, CAP07655; *T. equinum*, EGE08989. The *Claviceps* sequence was supplied by Prof. Paul Tudzynski, University of Münster (unpublished data). *F. oxysporum* sequences were obtained from NCBI (strain Fo5176) or the Broad Institute's *Fusarium* Comparative Database.
(TIF)

Figure S5 **Gibberellin biosynthesis in species of the GFC.** **A**: Expression of gibberellins biosynthetic genes *cps/ks* and *des* after three days of cultivation under nitrogen-limiting conditions (6 mM glutamine). All strains except for *F. verticillioides* contain the entire GA gene cluster. **B**: GC-MS analysis of *ent*-kaurene, the first specific intermediate of the GA pathway.
(TIF)

Figure S6 **Phylogenetic tree of PKSs encoded by** ***Fusarium*** **genome sequences.** The tree was generated by maximum parsimony analysis of alignments of deduced amino acid sequences of the KS and AT/MAT domains. The tree also includes PKSs from other fungi and for which the corresponding secondary metabolites is known. The names of metabolites are indicated to the right. R-PKS and NR-PKS indicate reducing and non-reducing PKS respectively. “MSAS” indicates 6-methylsalicylic acid synthase. The protein/gene designations FCIRG, FFUJ, FM, FOXG, FVEG and JGI correspond to PKSs deduced from genome sequences of *F. circinatum*, *F. mangiferae*, *F. oxysporum* f. sp. *lycopercisi* strain Fol 4287, *F. verticillioides*, and *F. solani* f. sp. *pisi* (*Nectria haematococca*) respectively. The designations FOXB, FOSC3A and PHW815 are for PKSs from *F. oxysporum* strains Fo5176, FOSC 3-a and PHW815 respectively. The tree is rooted with the KS and AT domains of the *Gallus gallus* fatty acid synthase.
(TIF)

Figure S7 **Comparison of the fusarubin biosynthetic gene (** ***FSR/PGL*** **) cluster in genomes sequences of** ***Fusarium*** **.** Horizontal arrows that are the same color represent genes, or gene sets, that have closely related homologues in two or more species. Blue arrows represent *FSR/PGL* genes, and the numbers within these arrows correspond to *FSR/PGL* gene numbers rather than gene/protein model designations from genome databases. For those that are available, gene designations are indicated below species names. In *F. solani*, all *FSR/PGL* genes are not located within a contiguous cluster of genes.
(TIF)

Figure S8 **Comparison of the putative fusaric acid biosynthetic gene (** ***FUB*** **) cluster in genomes sequences of** ***Fusarium*** **.** Horizontal arrows that are the same color represent genes, or gene sets, that have closely related homologues in two or more species. Blue arrows represent *FUB* genes, and the numbers within these arrows correspond to *FUB* gene numbers rather than gene/protein model designations from genome sequence databases. For those that are available, gene designations are indicated below species names. In *F. oxysporum*, the *FUB* cluster is interrupted by two genes. The asterisks mark genes without annotation.
(TIF)

Figure S9 **Comparison of the bikaverin biosynthetic (** ***BIK*** **) cluster in genomes sequences of** ***Fusarium*** **.** *BIK* genes are represented by blue horizontal arrows, and the numbers within these arrows correspond to *BIK* gene numbers rather than gene/protein model designations from genome sequence databases. For those that are available, gene designations are indicated below species names. The asterisks marks FFUJ_14916.
(TIF)

Figure S10 **Comparison of the fusarin biosynthetic gene (** ***FUS*** **) cluster in genome sequences of** ***Fusarium*** **and the related fungus** ***Metarhizium anisopliae*** **as well as remnants of the** ***FUS*** **cluster in** ***F. mangiferae*** **and** ***Trichoderma reesei***. FUS genes are represented by blue horizontal arrows, and the numbers in the arrows correspond to *FUS* gene numbers. For those that are available, gene designations are indicated below species names. Ψ indicates a pseudogene.
(TIF)

Figure S11 **Comparison of the putative** ***PKS17*** **–** ***PKS18*** **gene cluster in the genome sequences of** ***F. fujikuroi*** **and** ***F. mangiferae*** **.** The cluster genes are represented by green horizontal arrows, and cluster flanking genes are represented by blue or yellow arrows; yellow arrows represent genes that were not detected in the flanking regions of *F. fujikuroi* or *F. mangiferae*. Synteny of some cluster flanking regions is partially conserved in *F. verticillioides*, *F. circinatum*, and *F. oxysporum*, which lack the cluster. For those that are available, gene designations are indicated below species names.
(TIF)

Figure S12 **Comparison of the putative, two-gene** ***PKS12*** **clusters (green arrows) in genome sequences of** ***Fusarium*** **.** The second gene in the cluster is predicted to encode a methyltransferase. Blue arrows represent cluster flanking genes, which exhibit partial synteny conservation among the species examined. Based on phylogenetic analysis (Figure 5), PKS12 homologues have been arbitrarily designated as PKS12 and PKS12a until additional information becomes available. PKS12 is absent in the *F. solani* genome sequence, and only a remnant of it (indicated by Ψ) is present in the genome sequences of *F. oxysporum*. However, *F. solani* has a PKS12a homologue, and *F. oxysporum* strain Fol 4287 has two PKS12a paralogues (FOXG_14850 and FOXG_15586), which are part of a larger region of duplicated DNA that includes two putative methyltransferase genes, one on either side of each PKS12a paralogue. Other *F. oxysporum* genome sequences examined have only one PKS12a homologue.
(TIF)

Figure S13 **Phylogenetic tree of** ***Fusarium*** **NRPS22 and** ***Beauveria bassiana*** **BeaS and BslS as well as** ***F. scirpi*** **EsyN.** The tree was generated by maximum parsimony analysis of alignements of deduced amino acid sequences of the A domains. The protein/gene designations FCIRG, FFUJ, FOXG and FVEG correspond to NRPSs deduced from genome sequences of *F. circinatum*, *F. fujikuroi, F. oxysporum* f. sp. *lycopercisi* strain Fol 4287, and *F. verticillioides* respectively. The tree is rooted with the A1 domain of the *F. graminearum* NRPS19.
(TIF)

Figure S14 **Relative expression of the fumonisin biosynthetic gene** ***FUM1*** **from** ***F. fujikuroi*** **and** ***F. verticillioides*** **in rice and maize roots.** Rice (A) and maize (B) roots were infected with *Fusarium fujikuroi* and *F. verticillioides* spores and every 2 days RNA was isolated from three or five plants and used in real time PCR analysis. The expression levels were obtained using the delta-delta Ct and were normalized against three reference genes encoding a related actin, the GDP-mannose transporter and ubiquitin. The expression levels of the *F. verticillioides FUM1* at 2 days in rice was arbitrarily set as 1, and all other expression levels were reported relative to it.
(TIF)

Figure S15 **Venn diagram representing the distribution of nitrogen-and pH-regulated genes.** Number of differentially regulated genes in wild-type *F. fujikuroi* cultivated under conditions that vary in nitrogen content and pH. The data were obtained by microarray analyses. The conditions were as follows: 6 mM gln (glutamine) constitutes, acidic low nitrogen; 60 mM gln, acid high nitrogen; 6 mM NaNO_3_ alkaline low nitrogen; and 120 mM NaNO_3_ alkaline high nitrogen.
(TIF)

Figure S16 **Expression pattern and distribution of active histone marks at the A:** Fusaric acid (FUB) and **B:** at the fumonisin gene cluster.
(TIF)

Table S1 **Putative** ***F. fujikuroi*** **centromeric regions.** Coordinates on chromosomes (Chr) I to XII include 0.5–1 kb of euchromatic regions on either side of pericentric and centromeric DNA and no attempt has been made to separate pericentric and centromeric regions. As none of the 14 scaffolds contain telomere repeats, all chromosome ends are unfinished. “Incompl R” indicates that the right arm of the chromosome does not have heterochromatin as indicated by presence of H3K9me3.
(DOCX)

Table S2 **Nearest genes to** ***F. fujikuroi*** **centromeres and synteny with** ***F. verticillioides*** **.** Nearest *F. fujikuroi* genes (FFUJ locus ID) to the left and right of the predicted centromere on each chromosome was identified and compared to the location of predicted orthologs in *F. verticillioides* (FVEG locus ID). “+” and “−” indicate strands, “t” indicates last gene on a specific *F. verticillioides* contig, “i”, indicates internal, non-terminal gene.
(DOCX)

Table S3 **Gene family features, comparative to related species.**
(XLSX)

Table S4 **Identification of secondary metabolite gene clusters based on SMURF and sequence comparisons with related fungal genomes.**
(XLSX)

Table S5 **Gibberellin production by** ***Fusarium*** **spp.** **A**: Gibberellin production by *Fusarium* species of the GFC and *F. oxysporum* 4287 (outgroup). *F. oxysporum* 4287 does not contain a GA gene cluster, but produces GAs after transforming it with the entire GA gene cluster from *F. fujikuroi* (cos1). **B**: Gibberellin production by *F. oxysporum* isolates with an entire GA gene.
(DOCX)

Table S6 **Secondary metabolite production in** ***F. fujikuroi*** **IMI58289,** ***F. circinatum*** **Fsp34,** ***F. mangiferae*** **MRC7560,** ***F. verticillioides*** **3125 and** ***F. oxysporum*** **4287.** The strains were cultivated under four different culture conditions. Bikaverin, *O*-methylfusarubin, fusarins, fumonisin B_1_, fusaric acid and beauvericin were analyzed by HPLC-FTMS.
(DOCX)

Table S7 **Expression pattern of all genes under four standard growth conditions.** acidic and alkaline low-nitrogen (6 mM glutamine or 6 mM sodium nitrate) and acidic and alkaline high-nitrogen (60 mM glutamine or 120 mM sodium nitrate), as well as proteomic data and the presence of activation histone marks under two different growth conditions (acidic low and high-nitrogen).
(XLSX)

Table S8 **Primers used for all experiments.**
(DOCX)

Table S9 **Overview of the metabolites that were analyzed by HPLC-FTMS.** The analytes were identified by their retention time and isotope pattern compared to standard substances. For the estimation of the produced amount, the *m/z* of their most intense ions were observed.
(DOCX)

Table S10 **Overview of the analysed amino acids.** The amino acids that are obtained after acid hydrolysis of apicidin and the apicidin metabolite were identified based on the accurate mass of their [M+H]^+^ ions.
(DOCX)

## References

[1] Geiser DM, Ivey ML, Hakiza G, Juba JH, Miller SA (2005) *Gibberella xylarioides* (anamorph: *Fusarium xylarioides*), a causative agent of coffee wilt disease in Africa, is a previously unrecognized member of the *G. fujikuroi* species complex. Mycologia 97: 191–201. 16389971 10.3852/mycologia.97.1.191

[2] Leslie JF, Summerell BA. (2006) The *Fusarium* laboratory manual. Oxford, UK. 259–269

[3] Kvas M, Marasas WFO, Wingfield BD, Wingfield MJ, Steenkamp ET (2009) Diversity and evolution of *Fusarium* species in the *Gibberella fujikuroi* complex. Fungal Diversity 34: 1–21.

[4] Bearder JR. (1983) In vivo diterpenoid biosynthesis in G*ibberella fujikuroi*: The pathway after *ent*-kaurene. In: Crozier A, Ed. The biochemistry and physiology of gibberellins. New York: Praeger, pp. 251–387.

[5] Yabuta T, Sumiki T (1938) On the crystal of gibberellin, a substance to promote plant growth. J Agric Chem Soc Japan 14: 1526–1526.

[6] Yabuta T (1935) Biochemistry of the bakanae fungus of rice. Agr Hort 10: 17–22.

[7] Rademacher W. (1997) Gibberellins. In: Anke T, Ed. Fungal Biotechnology. London: Chapman & Hall, pp. 193–205.

[8] Bömke C, Tudzynski B (2009) Diversity, regulation, and evolution of the gibberellin biosynthetic pathway in fungi compared to plants and bacteria. Phytochemistry 70: 1876–1893. 19560174 10.1016/j.phytochem.2009.05.020

[9] Tudzynski B, Hölter K (1998) Gibberellin biosynthetic pathway in *Gibberella fujikuroi*: Evidence for a gene cluster. Fungal Genetics and Biology 25: 157–170. 9917370 10.1006/fgbi.1998.1095

[10] Mihlan M, Homann V, Liu T-D, Tudzynski B (2003) AREA directly mediates nitrogen regulation of gibberellin biosynthesis in *Gibberella fujikuroi*, but its activity is not affected by NMR. Mol Microbiol 47: 975–991. 12581353 10.1046/j.1365-2958.2003.03326.x

[11] Tudzynski B, Homann V, Feng B, Marzluf GA (1999) Isolation, characterization and disruption of the *areA* nitrogen regulatory gene of *Gibberella fujikuroi* . Molecular and General Genetics 261: 106–114. 10071216 10.1007/s004380050947

[12] Wiemann, P Tudzynski, B. (2013) The nitrogen regulation network and its impact on secondary metabolism and pathogenicity. In: Brown DW & Proctor R H (Eds), *Fusarium*: Genomics, Molecular and Cellular Biology. Norwich, UK: Caister Academic Press.

[13] Rodríguez-Ortiz R, Limón MC, Avalos J (2009) Regulation of carotenogenesis and secondary metabolism by nitrogen in wild-type *Fusarium fujikuroi* and carotenoid-overproducing mutants. Appl Environ Microbiol 75: 405–413. 19047398 10.1128/AEM.01089-08 PMC2620702

[14] Wiemann P, Willmann A, Straeten M, Kleigrewe K, Beyer M, et al (2009) Biosynthesis of the red pigment bikaverin in *Fusarium fujikuroi*: Genes, their function and regulation. Mol Microbiol 72: 931–946. 19400779 10.1111/j.1365-2958.2009.06695.x

[15] Studt L, Wiemann P, Kleigrewe K, Humpf H-, Tudzynski B (2012) Biosynthesis of fusarubins accounts for pigmentation of *Fusarium fujikuroi* perithecia. Appl Environ Microbiol 78: 4468–4480. 22492438 10.1128/AEM.00823-12 PMC3370568

[16] Studt L, Troncoso C, Gong F, Hedden P, Toomajian C, et al (2012) Segregation of secondary metabolite biosynthesis in hybrids of *Fusarium fujikuroi* and *Fusarium proliferatum* . Fungal Genet Biol49: 567–577. 10.1016/j.fgb.2012.05.005 22626844

[17] Kleigrewe K, Aydin F, Hogrefe K, Piecuch P, Bergander K, et al (2012) Structure elucidation of new fusarins revealing insights in the rearrangement mechanisms of the *Fusarium* mycotoxin fusarin C. J Agric Food Chem 60: 5497–5505. 22540270 10.1021/jf3009469

[18] Kleigrewe K, Niehaus E-, Wiemann P, Tudzynski B, Humpf H-U (2012) New approach via gene knockout and single-step chemical reaction for the synthesis of isotopically labeled fusarin C as an internal standard for the analysis of this *Fusarium* mycotoxin in food and feed samples. J Agric Food Chem 60: 8350–8355. 22877497 10.1021/jf302534x

[19] Galagan JE, Calvo SE, Cuomo C, Ma L-, Wortman JR, et al (2005) Sequencing of *Aspergillus nidulans* and comparative analysis with *A. fumigatus* and *A. oryzae* . Nature 438: 1105–1115. 16372000 10.1038/nature04341

[20] Pel HJ, De Winde JH, Archer DB, Dyer PS, Hofmann G, et al (2007) Genome sequencing and analysis of the versatile cell factory *Aspergillus niger* CBS 513.88. Nat Biotechnol 25: 221–231. 17259976 10.1038/nbt1282

[21] Van Den Berg MA, Albang R, Albermann K, Badger JH, Daran J-M, et al (2008) Genome sequencing and analysis of the filamentous fungus *Penicillium chrysogenum* . Nat Biotechnol 26: 1161–1168. 18820685 10.1038/nbt.1498

[22] Ma L-, Van Der Does HC, Borkovich KA, Coleman JJ, Daboussi M-, et al (2010) Comparative genomics reveals mobile pathogenicity chromosomes in *Fusarium* . Nature 464: 367–373. 20237561 10.1038/nature08850 PMC3048781

[23] Nowrousian M, Stajich JE, Chu M, Engh I, Espagne E, et al (2010) De novo assembly of a 40 mb eukaryotic genome from short sequence reads: *Sordaria macrospora*, a model organism for fungal morphogenesis. PLoS Genet 6 4: e1000891. 20386741 10.1371/journal.pgen.1000891 PMC2851567

[24] Amselem J, Cuomo CA, van Kan JAL, Viaud M, Benito EP, et al (2011) Genomic analysis of the necrotrophic fungal pathogens *Sclerotinia sclerotiorum* and *Botrytis cinerea* . PLoS Genetics 7 8: e1002230. 21876677 10.1371/journal.pgen.1002230 PMC3158057

[25] Gao Q, Jin K, Ying S-, Zhang Y, Xiao G, et al (2011) Genome sequencing and comparative transcriptomics of the model entomopathogenic fungi *Metarhizium anisopliae* and *M. acridum* . 7 1: e1001264. 10.1371/journal.pgen.1001264 PMC3017113 21253567

[26] Kubicek CP, Herrera-Estrella A, Seidl-Seiboth V, Martinez DA, Druzhinina IS, et al (2011) Comparative genome sequence analysis underscores mycoparasitism as the ancestral life style of *Trichoderma* . Genome Biol 12 4: R40. 21501500 10.1186/gb-2011-12-4-r40 PMC3218866

[27] Martinez DA, Oliver BG, Gräser Y, Goldberg JM, Li W, et al (2012) Comparative genome analysis of *Trichophyton rubrum* and related dermatophytes reveals candidate genes involved in infection. Mbio 3 5: e00259–00212. 22951933 10.1128/mBio.00259-12 PMC3445971

[28] O'Connell RJ, Thon MR, Hacquard S, Amyotte SG, Kleemann J, et al (2012) Lifestyle transitions in plant pathogenic *Colletotrichum* fungi deciphered by genome and transcriptome analyses. Nat Genet 44: 1060–1065. 22885923 10.1038/ng.2372 PMC9754331

[29] Schardl CL, Young CA, Hesse U, Amyotte SG, Andreeva K, et al (2013) Plant-symbiotic fungi as chemical engineers: Multi-genome analysis of the *Clavicipitaceae* reveals dynamics of alkaloid loci. PLoS Genetics 9 2: e1003323. 23468653 10.1371/journal.pgen.1003323 PMC3585121

[30] Condon BJ, Leng Y, Wu D, Bushley KE, Ohm RA, et al (2013) Comparative genome structure, secondary metabolite, and effector coding capacity across *Cochliobolus* pathogens. PLoS Genetics 9 1: e1003233. 23357949 10.1371/journal.pgen.1003233 PMC3554632

[31] Gaffoor I, Brown DW, Plattner R, Proctor RH, Qi W, et al (2005) Functional analysis of the polyketide synthase genes in the filamentous fungus *Gibberella zeae* (anamorph *Fusarium graminearum*). Eukaryotic Cell 4: 1926–1933. 16278459 10.1128/EC.4.11.1926-1933.2005 PMC1287854

[32] Hansen FT, Sørensen JL, Giese H, Sondergaard TE, Frandsen RJN (2012) Quick guide to polyketide synthase and nonribosomal synthetase genes in *Fusarium* . Int J Food Microbiol 155: 128–136. 22377171 10.1016/j.ijfoodmicro.2012.01.018

[33] Kroken S, Glass NL, Taylor JW, Yoder OC, Turgeon BG (2003) Phylogenomic analysis of type I polyketide synthase genes in pathogenic and saprobic ascomycetes. Proc Natl Acad Sci U S A 100: 15670–15675. 14676319 10.1073/pnas.2532165100 PMC307626

[34] Brown DW, Butchko RAE, Baker SE, Proctor RH (2012) Phylogenomic and functional domain analysis of polyketide synthases in *Fusarium* . Fungal Biol 116: 318–331. 22289777 10.1016/j.funbio.2011.12.005

[35] Wingfield BD, Steenkamp ET, Santana Q, Coetzee MPA, Bam S, et al (2012) First fungal genome sequence from Africa: Consequences scientific and regional. South African Journal of Science 108: 1–9.

[36] Freeman S, Maimon M, Pinkas Y (1999) Use of GUS transformants of *Fusarium subglutinans* for determining etiology of mango malformation disease. Phytopathology 89: 456–461. 18944716 10.1094/PHYTO.1999.89.6.456

[37] Gamliel-Atinsky E, Sztejnberg A, Maymon M, Vintal H, Shtienberg D, Freeman S (2009) Infection dynamics of *Fusarium mangiferae*, causal agent of mango malformation disease. Phytopathology 99: 775–781. 19453238 10.1094/PHYTO-99-6-0775

[38] Xu J-, Leslie JF (1996) A genetic map of *Gibberella fujikuroi* mating population A (*Fusarium moniliforme*). Genetics 143: 175–189. 8722773 10.1017/s0016672300034066 PMC1207252

[39] Linnemannstöns P, Voß T, Hedden P, Gaskin P, Tudzynski B (1999) Deletions in the gibberellin biosynthesis gene cluster of *Gibberella fujikuroi* by restriction enzyme-mediated integration and conventional transformation-mediated mutagenesis. Appl Environ Microbiol 65: 2558–2564. 10347043 10.1128/aem.65.6.2558-2564.1999 PMC91378

[40] Parra G, Bradnam K, Ning Z, Keane T, Korf I (2009) Assessing the gene space in draft genomes. Nucleic Acids Res 37: 289–297. 19042974 10.1093/nar/gkn916 PMC2615622

[41] Aguileta G, Marthey S, Chiapello H, Lebrun MH, Rodolphe F, et al (2008) Assessing the performance of single-copy genes for recovering robust phylogenies. Syst Biol 57: 613–627. 18709599 10.1080/10635150802306527

[42] Rattei T, Tischler P, Götz S, Jehl M, Hoser J, et al (2009) SIMAP- A comprehensive database of pre-calculated protein sequence similarities, domains, annotations and clusters. Nucleic Acids Res 38 SUPPL.1: D223–D226. 19906725 10.1093/nar/gkp949 PMC2808863

[43] Ruepp A, Zollner A, Maier D, Albermann K, Hani J, et al (2004) The FunCat, a functional annotation scheme for systematic classification of proteins from whole genomes. Nucleic Acids Res 32: 5539–5545. 15486203 10.1093/nar/gkh894 PMC524302

[44] Han Y, Liu X, Benny U, Corby Kistler H, VanEtten HD (2001) Genes determining pathogenicity to pea are clustered on a supernumerary chromosome in the fungal plant pathogen *Nectria haematococca* . Plant Journal 25: 305–314. 11208022 10.1046/j.1365-313x.2001.00969.x

[45] Coleman JJ, Rounsley SD, Rodriguez-Carres M, Kuo A, Wasmann CC, et al (2009) The genome of *Nectria haematococca*: Contribution of supernumerary chromosomes to gene expansion. PLoS Genetics 5 8: e1000618. 19714214 10.1371/journal.pgen.1000618 PMC2725324

[46] Smith KM, Galazka JM, Phatale PA, Connolly LR, Freitag M (2012) Centromeres of filamentous fungi. Chromosome Research 20: 635–656. 22752455 10.1007/s10577-012-9290-3 PMC3409310

[47] Smith KM, Phatale PA, Sullivan CM, Pomraning KR, Freitag M (2011) Heterochromatin is required for normal distribution of *Neurospora crassa* CenH3. Mol Cell Biol 31: 2528–2542. 21505064 10.1128/MCB.01285-10 PMC3133421

[48] Ma J, Wing RA, Bennetzen JL, Jackson SA (2007) Evolutionary history and positional shift of a rice centromere. Genetics 177: 1217–1220. 17660534 10.1534/genetics.107.078709 PMC2034625

[49] Schueler MG, Sullivan BA (2006) Structural and functional dynamics of human centromeric chromatin. Annu Rev Genomics Hum Genet. 2006 7: 301–313. 10.1146/annurev.genom.7.080505.115613 16756479

[50] Jurka J, Kapitonov VV, Pavlicek A, Klonowski P, Kohany O, et al (2005) Repbase update, a database of eukaryotic repetitive elements. Cytogenetic and Genome Research 110: 462–467. 16093699 10.1159/000084979

[51] Katsuyama Y, Ohnishi Y (2012) Type III polyketide synthases in microorganisms. Methods in Enzymology 515: 359–377. 22999182 10.1016/B978-0-12-394290-6.00017-3

[52] Smith, KM Phatale, PA Bredeweg, EL Pomraning, KR Freitag, M. (2012) Epigenetics of filamentous fungi. In: Meyers RA, Ed. Epigenetic Regulation and Epigenomics (Current Topics from the Encyclopedia of Molecular Cell Biolo). Wiley-VCH Verlag GmbH & Co. pp. 1063–1107.

[53] Strauss J, Reyes-Dominguez Y (2011) Regulation of secondary metabolism by chromatin structure and epigenetic codes. Fungal Genetics and Biology 48: 62–69. 20659575 10.1016/j.fgb.2010.07.009 PMC3935439

[54] Bannister AJ, Kouzarides T (2011) Regulation of chromatin by histone modifications. Cell Res 21: 381–395. 21321607 10.1038/cr.2011.22 PMC3193420

[55] Williams RB, Henrikson JC, Hoover AR, Lee AE, Cichewicz RH (2008) Epigenetic remodeling of the fungal secondary metabolome. Org Biomol Chem 6: 1895–1897. 18480899 10.1039/b804701d

[56] Bok JW, Chiang YM, Szewczyk E, Reyes-Dominguez Y, Davidson AD, et al (2009) Chromatin-level regulation of biosynthetic gene clusters. Nat Chem Biol 5: 462–464. 19448638 10.1038/nchembio.177 PMC2891026

[57] Soukup AA, Chiang Y-, Bok JW, Reyes-Dominguez Y, Oakley BR, et al (2012) Overexpression of the *Aspergillus nidulans* histone 4 acetyltransferase EsaA increases activation of secondary metabolite production. Mol Microbiol 86: 314–330. 22882998 10.1111/j.1365-2958.2012.08195.x PMC3514908

[58] Freitag M, Hickey PC, Raju NB, Selker EU, Read ND (2004) GFP as a tool to analyze the organization, dynamics and function of nuclei and microtubules in *Neurospora* . Fungal Genet Biol 41: 897–910. 15341912 10.1016/j.fgb.2004.06.008

[59] Cichewicz RH (2010) Epigenome manipulation as a pathway to new natural product scaffolds and their congeners. Nat Prod Rep 27: 11–22. 20024091 10.1039/b920860g PMC2958777

[60] Reyes-Dominguez Y, Bok JW, Berger H, Shwab EK, Basheer A, et al (2010) Heterochromatic marks are associated with the repression of secondary metabolism clusters in *Aspergillus nidulans* . Mol Microbiol 76: 1376–1386. 20132440 10.1111/j.1365-2958.2010.07051.x PMC2904488

[61] Markham JE, Hille J (2001) Host-selective toxins as agents of cell death in plant–fungus interactions. Mol Plant Pathol 2: 229–239. 20573011 10.1046/j.1464-6722.2001.00066.x

[62] Howlett BJ (2006) Secondary metabolite toxins and nutrition of plant pathogenic fungi. Curr Opin Plant Biol 9: 371–375. 16713733 10.1016/j.pbi.2006.05.004

[63] Malonek S, Bömke C, Bornberg-Bauer E, Rojas MC, Hedden P, et al (2005) Distribution of gibberellin biosynthetic genes and gibberellin production in the *Gibberella fujikuroi* species complex. Phytochemistry 66: 1296–311. 15925394 10.1016/j.phytochem.2005.04.012

[64] Troncoso C, González X, Bömke C, Tudzynski B, Gong F, et al (2010) Gibberellin biosynthesis and gibberellin oxidase activities in *Fusarium sacchari*, *Fusarium konzum* and *Fusarium subglutinans* strains. Phytochemistry 71: 1322–1331. 20570295 10.1016/j.phytochem.2010.05.006

[65] Tsavkelova EA, Bömke C, Netrusov AI, Weiner J, Tudzynski B (2008) Production of gibberellic acids by an orchid-associated *Fusarium proliferatum* strain. Fungal Genetics and Biology 45: 1393–1403. 18694840 10.1016/j.fgb.2008.07.011

[66] Bömke C, Rojas MC, Hedden P, Tudzynski B (2008) Loss of gibberellin production in *Fusarium verticillioides* (*Gibberella fujikuroi* MP-A) is due to a deletion in the gibberellic acid gene cluster. Appl Environ Microbiol 74: 7790–7801. 18952870 10.1128/AEM.01819-08 PMC2607190

[67] Bömke C, Rojas MC, Gong F, Hedden P, Tudzynski B (2008) Isolation and characterization of the gibberellin biosynthetic gene cluster in *Sphaceloma manihoticola* . Appl Environ Microbiol 74: 5325–5339. 18567680 10.1128/AEM.00694-08 PMC2546651

[68] Kawaide H (2006) Biochemical and molecular analyses of gibberellin biosynthesis in fungi. Bioscience, Biotechnology and Biochemistry 70: 583–590. 10.1271/bbb.70.583 16556972

[69] Slot JC, Rokas A (2011) Horizontal transfer of a large and highly toxic secondary metabolic gene cluster between fungi. Current Biology 21: 134–139. 21194949 10.1016/j.cub.2010.12.020

[70] Campbell MA, Rokas A, Slot JC (2012) Horizontal transfer and death of a fungal secondary metabolic gene cluster. Genome Biol Evol 4: 289–293. 22294497 10.1093/gbe/evs011 PMC3318441

[71] Schumacher J, Gautier A, Morgant G, Studt L, Ducrot P-, et al (2013) A functional bikaverin biosynthesis gene cluster in rare strains of *Botrytis cinerea* is positively controlled by VELVET. Plos One 8 1: e53729. 23308280 10.1371/journal.pone.0053729 PMC3538735

[72] Awakawa T, Kaji T, Wakimoto T, Abe I (2012) A heptaketide naphthaldehyde produced by a polyketide synthase from *Nectria haematococca* . Bioorganic and Medicinal Chemistry Letters 22: 4338–4340. 22633689 10.1016/j.bmcl.2012.05.005

[73] Proctor RH, Butchko RAE, Brown DW, Moretti A (2007) Functional characterization, sequence comparisons and distribution of a polyketide synthase gene required for perithecial pigmentation in some *Fusarium* species. Food Addit Contam 24: 1076–1087. 17886180 10.1080/02652030701546495

[74] Linnemannstöns P, Schulte J, Del Mar Prado M, Proctor RH, Avalos J, et al (2002) The polyketide synthase gene *pks4* from *Gibberella fujikuroi* encodes a key enzyme in the biosynthesis of the red pigment bikaverin. Fungal Genetics and Biology 37: 134–148. 12409099 10.1016/s1087-1845(02)00501-7

[75] Brown DW, Butchko RAE, Busman M, Proctor RH (2012) Identification of gene clusters associated with fusaric acid, fusarin, and perithecial pigment production in *Fusarium verticillioides* . Fungal Genetics and Biology 49: 521–532. 22652150 10.1016/j.fgb.2012.05.010

[76] Brown DW, Butchko RAE, Proctor RH (2008) Genomic analysis of *Fusarium verticillioides* . Food Additives and Contaminants - Part A Chemistry, Analysis, Control, Exposure and Risk Assessment 25: 1158–1165. 19238625

[77] Krasnoff SB, Sommers CH, Moon Y-, Donzelli BGG, Vandenberg JD, et al (2006) Production of mutagenic metabolites by *Metarhizium anisopliae* . J Agric Food Chem 54: 7083–7088. 16968066 10.1021/jf061405r

[78] Díaz-Sánchez V, Avalos J, Limón MC (2012) Identification and regulation of *fusA*, the polyketide synthase gene responsible for fusarin production in *Fusarium fujikuroi* . Appl Environ Microbiol 78: 7258–7266. 22865073 10.1128/AEM.01552-12 PMC3457117

[79] Proctor RH, Busman M, Seo J-, Lee YW, Plattner RD (2008) A fumonisin biosynthetic gene cluster in *Fusarium oxysporum* strain O-1890 and the genetic basis for B versus C fumonisin production. Fungal Genetics and Biology 45: 1016–1026. 18375156 10.1016/j.fgb.2008.02.004

[80] Brown DW, Butchko RAE, Busman M, Proctor RH (2007) The *Fusarium verticillioides* FUM gene cluster encodes a Zn(II)2Cys6 protein that affects FUM gene expression and fumonisin production. Eukaryotic Cell 6: 1210–1218. 17483290 10.1128/EC.00400-06 PMC1951116

[81] Proctor RH, Plattner RD, Desjardins AE, Busman M, Butchko RAE (2006) Fumonisin production in the maize pathogen *Fusarium verticillioides*: Genetic basis of naturally occurring chemical variation. J Agric Food Chem 54: 2424–2430. 16536629 10.1021/jf0527706

[82] Proctor RH, Plattner RD, Brown DW, Seo J-, Lee Y- (2004) Discontinuous distribution of fumonisin biosynthetic genes in the *Gibberella fujikuroi* species complex. Mycol Res 108: 815–822. 15446715 10.1017/s0953756204000577

[83] Khaldi N, Wolfe KH (2011) Evolutionary origins of the fumonisin secondary metabolite gene cluster in *Fusarium verticillioides and Aspergillus niger* . Int J Evol Biol 2011: 423821. 21716743 10.4061/2011/423821 PMC3119522

[84] Chiang Y-, Szewczyk E, Davidson AD, Keller N, Oakley BR, et al (2009) A gene cluster containing two fungal polyketide synthases encodes the biosynthetic pathway for a polyketide, asperfuranone, in *Aspergillus nidulans* . J Am Chem Soc 131: 2965–2970. 19199437 10.1021/ja8088185 PMC2765542

[85] Emery T (1980) Malonichrome, a new iron chelate from *Fusarium roseum* . Biochim Biophys Acta 629: 382–390. 7388041 10.1016/0304-4165(80)90110-5

[86] Tobiasen C, Aahman J, Ravnholt KS, Bjerrum MJ, Grell MN, et al (2007) Nonribosomal peptide synthetase (NPS) genes in *Fusarium graminearum*, *F. culmorum* and *F. pseudograminearium* and identification of NPS2 as the producer of ferricrocin. Curr Genet 51: 43–58. 17043871 10.1007/s00294-006-0103-0

[87] Bushley KE, Ripoll DR, Turgeon BG (2008) Module evolution and substrate specificity of fungal nonribosomal peptide synthetases involved in siderophore biosynthesis. BMC Evolutionary Biology 8: 328. 19055762 10.1186/1471-2148-8-328 PMC2644324

[88] Wiemann P, Albermann S, Niehaus E-, Studt L, von Bargen KW, et al (2012) The Sfp-type 4′-phosphopantetheinyl transferase Ppt1 of *Fusarium fujikuroi* controls evelopment, secondary metabolism and pathogenicity. Plos One 7 5:e37519. 22662164 10.1371/journal.pone.0037519 PMC3360786

[89] Pieper R, Kleinkauf H, Zocher R (1992) Enniatin synthetases from different fusaria exhibiting distinct amino acid specificities. J Antibiot 45: 1273–1277. 1399848 10.7164/antibiotics.45.1273

[90] Haese A, Pieper R, Von Ostrowski T, Zocher R (1994) Bacterial expression of catalytically active fragments of the multifunctional enzyme enniatin synthetase. J Mol Biol 243: 116–122. 7932733 10.1006/jmbi.1994.1634

[91] Xu Y, Orozco R, Kithsiri Wijeratne EM, Espinosa-Artiles P, Leslie Gunatilaka AA, et al (2009) Biosynthesis of the cyclooligomer depsipeptide bassianolide, an insecticidal virulence factor of *Beauveria bassiana* . Fungal Genetics and Biology 46: 353–364. 19285149 10.1016/j.fgb.2009.03.001

[92] López-Berges MS, Hera C, Sulyok M, Schäfer K, Capilla J, et al (2013) The velvet complex governs mycotoxin production and virulence of *Fusarium oxysporum* on plant and mammalian hosts. Mol Microbiol 87: 49–65. 23106229 10.1111/mmi.12082

[93] Glenn AE, Zitomer NC, Zimeri AM, Williams LD, Riley RT, et al (2008) Transformation-mediated complementation of a FUM gene cluster deletion in *Fusarium verticillioides* restores both fumonisin production and pathogenicity on maize seedlings. Mol Plant-Microbe Interact 21: 87–97. 18052886 10.1094/MPMI-21-1-0087

[94] Gao X, Shim WB, Göbel C, Kunze S, Feussner I, et al (2007) Disruption of a maize 9-lipoxygenase results in increased resistance to fungal pathogens and reduced levels of contamination with mycotoxin fumonisin. Mol Plant Microbe Interact 20: 922–933. 17722696 10.1094/MPMI-20-8-0922

[95] Brakhage AA (2013) Regulation of fungal secondary metabolism. Nat Rev Microbiol. 11: 21–32. 10.1038/nrmicro2916 23178386

[96] Nützmann HW, Schroeckh V, Brakhage AA (2012) Regulatory cross talk and microbial induction of fungal secondary metabolite gene clusters. Methods Enzymol 517: 325–341. 23084946 10.1016/B978-0-12-404634-4.00016-4

[97] Burger G, Strauss J, Scazzocchio C, Lang BF (1991) *NirA*, the pathway-specific regulatory gene of nitrate assimilation in *Aspergillus nidulans*, encodes a putative GAL4-type zinc finger protein and contains four introns in highly conserved regions. Mol Cell Biol 11: 5746–5755. 1922075 10.1128/mcb.11.11.5746-5755.1991 PMC361946

[98] Schinko T, Berger H, Lee W, Gallmetzer A, Pirker K, et al (2010) Transcriptome analysis of nitrate assimilation in *Aspergillus nidulans* reveals connections to nitric oxide metabolism. Mol Microbiol 78: 720–738. 20969648 10.1111/j.1365-2958.2010.07363.x PMC3020322

[99] Müller S, Baldin C, Groth M, Guthke R, Kniemeyer O, et al (2012) Comparison of transcriptome technologies in the pathogenic fungus *Aspergillus fumigatus* reveals novel insights into the genome and MpkA dependent gene expression. BMC Genomics 13: 519. 23031507 10.1186/1471-2164-13-519 PMC3505472

[100] Picotti P, Bodenmiller B, Mueller LN, Domon B, Aebersold R (2009) Full dynamic range proteome analysis of *Saccharomyces cerevisiae* by targeted proteomics. Cell 138: 795–806. 19664813 10.1016/j.cell.2009.05.051 PMC2825542

[101] Karimi-Aghcheh R, Bok JW, Phatale PA, Smith KM, Baker SE, et al (2013) Functional analyses of *Trichoderma reesei* LAE1 reveal conserved and contrasting roles of this regulator. G3 (Bethesda) 3: 369–378. 23390613 10.1534/g3.112.005140 PMC3564997

[102] Roze LV, Arthur AE, Hong S-, Chanda A, Linz JE (2007) The initiation and pattern of spread of histone H4 acetylation parallel the order of transcriptional activation of genes in the aflatoxin cluster. Mol Microbiol 66: 713–726. 17919289 10.1111/j.1365-2958.2007.05952.x

[103] Nützmann H-, Reyes-Dominguez Y, Scherlach K, Schroeckh V, Horn F, et al (2011) Bacteria-induced natural product formation in the fungus *Aspergillus nidulans* requires Saga/Ada-mediated histone acetylation. Proc Natl Acad Sci U S A 108: 14282–14287. 21825172 10.1073/pnas.1103523108 PMC3161617

[104] Kim H, Woloshuk CP (2008) Role of AREA, a regulator of nitrogen metabolism, during colonization of maize kernels and fumonisin biosynthesis in *Fusarium verticillioides* . Fungal Genetics and Biology 45: 947–953. 18440841 10.1016/j.fgb.2008.03.007

[105] Han J-, Ahn Seong Hoon, Park Seung Hee, Wang So Young, Bae G-, et al (2000) Apicidin, a histone deacetylase inhibitor, inhibits proliferation of tumor cells via induction of p21(WAF1/Cip1) and gelsolin. Cancer Res 60: 6068–6074. 11085529

[106] Jin J-, Lee S, Lee J, Baek S-, Kim J-, et al (2010) Functional characterization and manipulation of the apicidin biosynthetic pathway in *Fusarium semitectum* . Mol Microbiol 76: 456–466. 20233305 10.1111/j.1365-2958.2010.07109.x

[107] Darken MA, Jensen AL, Shu P (1959) Production of gibberellic acid by fermentation. Appl Microbiol 7: 301–303. 13814121 10.1128/am.7.5.301-303.1959 PMC1057525

[108] Geissman TA, Verbiscar AJ, Phinney BO, Cragg G (1966) Studies on the biosynthesis of gibberellins from *ent*-kaurenoic acid in cultures of *Gibberella fujikuroi* . Phytochemistry 5: 933–947.

[109] Cenis JL (1992) Rapid extraction of fungal DNA for PCR amplification. Nucleic Acids Res 20: 2380. 1594460 10.1093/nar/20.9.2380 PMC312363

[110] Pöggler S, Masloff S, Hoff B, Mayrhofer S, Kück U (2003) Versatile EGFP reporter plasmids for cellular localization of recombinant gene products in filamentous fungi. Curr Genet 43: 54–61. 12684845 10.1007/s00294-003-0370-y

[111] Staben C, Jensen B, Singer M, Pollock J, Schechtman M, et al (1989) Use of a bacterial hygromycin B resistance gene as a dominant selectable marker in *Neurospora crassa* transformation. Fungal Genet Newsl 36: 79–81.

[112] Malonek S, Rojas MC, Hedden P, Gaskin P, Hopkins P, et al (2004) The NADPH-cytochrome P450 reductase gene from *Gibberella fujikuroi* is essential for gibberellin biosynthesis. J Biol Chem 279: 25075–25084. 15037621 10.1074/jbc.M308517200

[113] Christianson TW, Sikorski RS, Dante M, Shero JH, Hieter P (1992) Multifunctional yeast high-copy-number shuttle vectors. Gene 110: 119–122. 1544568 10.1016/0378-1119(92)90454-w

[114] Colot HV, Park G, Turner GE, Ringelberg C, Crew CM, et al (2006) Erratum: A high-throughput gene knockout procedure for *Neurospora* reveals functions for multiple transcription factors. Proc Natl Acad Sci U S A 103: 16614. 10.1073/pnas.0601456103 PMC1482798 16801547

[115] Park G, Colot HV, Collopy PD, Krystofova S, Crew C, et al (2011) High-throughput production of gene replacement mutants in *Neurospora crassa* . Methods Mol Biol 722: 179–189. 21590421 10.1007/978-1-61779-040-9_13 PMC3671941

[116] Sambrook J, Fritsch EF, Maniatis T. (1989) Molecular cloning: A laboratory manual. 2nd edn. Cold Spring Harbor, NY: Cold Spring Harbor Laboratory Press.

[117] Pfaffl MW (2001) A new mathematical model for relative quantification in real-time RT-PCR. Nucleic Acids Res 29 9:e45. 11328886 10.1093/nar/29.9.e45 PMC55695

[118] Schumacher J (2012) Tools for *Botrytis cinerea*: New expression vectors make the gray mold fungus more accessible to cell biology approaches. Fungal Genet Biol 49: 483–497. 22503771 10.1016/j.fgb.2012.03.005

[119] Larson TM, Kendra DF, Busman M, Brown DW (2011) *Fusarium verticillioides* chitin synthases CHS5 and CHS7 are required for normal growth and pathogenicity. Curr Genet 57: 177–189. 21246198 10.1007/s00294-011-0334-6

[120] Darling ACE, Mau B, Blattner FR, Perna NT (2004) Mauve: Multiple alignment of conserved genomic sequence with rearrangements. Genome Res 14: 1394–1403. 15231754 10.1101/gr.2289704 PMC442156

[121] Delcher AL, Phillippy A, Carlton J, Salzberg SL (2002) Fast algorithms for large-scale genome alignment and comparison. Nucleic Acids Res 30: 2478–2483. 12034836 10.1093/nar/30.11.2478 PMC117189

[122] Salamov AA, Solovyev VV (2000) Ab initio gene finding in *Drosophila* genomic DNA. Genome Res 10: 516–522. 10779491 10.1101/gr.10.4.516 PMC310882

[123] Ter-Hovhannisyan V, Lomsadze A, Chernoff YO, Borodovsky M (2008) Gene prediction in novel fungal genomes using an ab initio algorithm with unsupervised training. Genome Res 18: 1979–1990. 18757608 10.1101/gr.081612.108 PMC2593577

[124] Stanke M, Keller O, Gunduz I, Hayes A, Waack S, et al (2006) AUGUSTUS: Ab initio prediction of alternative transcripts. Nucleic Acids Res 34: W435–W439. 16845043 10.1093/nar/gkl200 PMC1538822

[125] Donlin MJ (2009) Using the generic genome browser (GBrowse). Current Protocols in Bioinformatics SUPPL. 28: 9.9.1–9.9.25. 10.1002/0471250953.bi0909s28 19957275

[126] Lowe TM, Eddy SR (1997) tRNAscan-SE: A program for improved detection of transfer RNA genes in genomic sequence. Nucleic Acids Res 25: 955–964. 9023104 10.1093/nar/25.5.955 PMC146525

[127] Marthey S, Aguileta G, Rodolphe F, Gendrault A, Giraud T, et al (2008) Funybase: A fungal phylogenomic database. BMC Bioinformatics 9: 456. 18954438 10.1186/1471-2105-9-456 PMC2600828

[128] Price AL, Jones NC, Pevzner PA (2005) De novo identification of repeat families in large genomes. Bioinformatics SUPPL. 1: i351–i358. 15961478 10.1093/bioinformatics/bti1018

[129] Wootton JC, Federhen S (1993) Statistics of local complexity in amino acid sequences and sequence databases. Computers and Chemistry 17 2: 149–163.

[130] Benson G (1999) Tandem repeats finder: A program to analyze DNA sequences. Nucleic Acids Res 27: 573–580. 9862982 10.1093/nar/27.2.573 PMC148217

[131] Xu Z, Wang H (2007) LTR-FINDER: An efficient tool for the prediction of full-length LTR retrotransposons. Nucleic Acids Res 35 SUPPL.2: W265–W268. 17485477 10.1093/nar/gkm286 PMC1933203

[132] Jiang Z, Hubley R, Smit A, Eichler EE (2008) DupMasker: A tool for annotating primate segmental duplications. Genome Res 18: 1362–1368. 18502942 10.1101/gr.078477.108 PMC2493431

[133] Walter MC, Rattei T, Arnold R, Güldener U, Münsterkötter M, et al (2009) PEDANT covers all complete RefSeq genomes. Nucleic Acids Res SUPPL. 1: D408–D411. 10.1093/nar/gkn749 PMC2686588 18940859

[134] Wong P, Walter M, Lee W, Mannhaupt G, Münsterkötter M, et al (2011) FGDB: Revisiting the genome annotation of the plant pathogen *Fusarium graminearum* . Nucleic Acids Res SUPPL. 1: D637–D639. 21051345 10.1093/nar/gkq1016 PMC3013644

[135] Emanuelsson O, Nielsen H, Brunak S, Von Heijne G (2000) Predicting subcellular localization of proteins based on their N-terminal amino acid sequence. J Mol Biol 300: 1005–1016. 10891285 10.1006/jmbi.2000.3903

[136] Tamura T, Akutsu T (2007) Subcellular location prediction of proteins using support vector machines with alignment of block sequences utilizing amino acid composition. BMC Bioinformatics 8: 466. 18047679 10.1186/1471-2105-8-466 PMC2220007

[137] Krogh A, Larsson B, Von Heijne G, Sonnhammer ELL (2001) Predicting transmembrane protein topology with a hidden markov model: Application to complete genomes. J Mol Biol 305: 567–580. 11152613 10.1006/jmbi.2000.4315

[138] Petersen TN, Brunak S, Von Heijne G, Nielsen H (2011) SignalP 4.0: Discriminating signal peptides from transmembrane regions. Nature Methods 8: 785–786. 21959131 10.1038/nmeth.1701

[139] Katoh K, Asimenos G, Toh H (2009) Multiple alignment of DNA sequences with MAFFT. Methods in Molecular Biology 537: 39–64. 19378139 10.1007/978-1-59745-251-9_3

[140] Guindon S, Dufayard J-, Lefort V, Anisimova M, Hordijk W, et al (2010) New algorithms and methods to estimate maximum-likelihood phylogenies: Assessing the performance of PhyML 3.0. Syst Biol 59: 307–321. 20525638 10.1093/sysbio/syq010

[141] Tamura K, Peterson D, Peterson N, Stecher G, Nei M, et al (2011) MEGA5: Molecular evolutionary genetics analysis using maximum likelihood, evolutionary distance, and maximum parsimony methods. Mol Biol Evol 28: 2731–2739. 21546353 10.1093/molbev/msr121 PMC3203626

[142] Carvalho BS, Irizarry RA (2010) A framework for oligonucleotide microarray preprocessing. Bioinformatics 26: 2363–2367. 20688976 10.1093/bioinformatics/btq431 PMC2944196

[143] Irizarry RA, Hobbs B, Collin F, Beazer-Barclay YD, Antonellis KJ, et al (2003) Exploration, normalization, and summaries of high density oligonucleotide array probe level data. Biostatistics (Oxford, England) 4: 249–264. 10.1093/biostatistics/4.2.249 12925520

[144] Smyth GK, Gentleman R, Carey V, Dudoit S, Irizarry R, et al.. (2005) Limma: Linear models for microarray data. In: Gentleman R, Carey, V Dudoit, S Irizarry, R Huber, W. (Eds), Bioinformatics and Computational Biology Solutions using R and Bioconductor. New York: Springer. pp. 397–420.

[145] Benjamini Y, Hochberg Y (1995) Controlling the false discovery rate: A practical and powerful approach to multiple testing. Journal of the Royal Statistical Society.Series B (Methodological) 57: 289–300.

[146] Smith KM, Dobosy JR, Reifsnyder JE, Rountree MR, Anderson DC, et al (2010) H2B- and H3-specific histone deacetylases are required for DNA methylation in *Neurospora crassa* . Genetics 186: 1207–1216. 20876559 10.1534/genetics.110.123315 PMC2998305

[147] Pomraning KR, Smith KM, Bredeweg EL, Connolly LR, Phatale PA, et al (2012) Library preparation and data analysis packages for rapid genome sequencing. Methods Mol Biol 944: 1–22. 23065605 10.1007/978-1-62703-122-6_1 PMC3686501

[148] Li H, Durbin R (2009) Fast and accurate short read alignment with Burrows-Wheeler transform. Bioinformatics 25: 1754–1760. 19451168 10.1093/bioinformatics/btp324 PMC2705234

[149] Stein LD, Mungall C, Shu S, Caudy M, Mangone M, et al (2002) The generic genome browser: a building block for a model organism system database. Genome Res 12: 1599–1610. 12368253 10.1101/gr.403602 PMC187535

[150] Hebenstreit D, Gu M, Haider S, Turner DJ, Liò P, et al (2011) EpiChIP: gene-by-gene quantification of epigenetic modification levels. Nucleic Acids Res 39: e27. 21131282 10.1093/nar/gkq1226 PMC3061070

[151] Kleigrewe K, Söhnel A-, Humpf H- (2011) A new high-performance liquid chromatography-tandem mass spectrometry method based on dispersive solid phase extraction for the determination of the mycotoxin fusarin C in corn ears and processed corn samples. J Agric Food Chem 59: 10470–10476. 21894895 10.1021/jf2026814

[152] Hübner F, Harrer H, Fraske A, Kneifel S, Humpf H- (2012) Large scale purification of B-type fumonisins using centrifugal partition chromatography (CPC). Mycotoxin Research 28: 37–43. 23605981 10.1007/s12550-011-0114-7

[153] Terashima M, Specht M, Naumann B, Hippler M (2010) Characterizing the anaerobic response of *Chlamydomonas reinhardtii* by quantitative proteomics. Molecular and Cellular Proteomics 9: 1514–1532. 20190198 10.1074/mcp.M900421-MCP200 PMC2938099

[154] Kalli A, Hess S (2012) Effect of mass spectrometric parameters on peptide and protein identification rates for shotgun proteomic experiments on an LTQ-orbitrap mass analyzer. Proteomics 12: 21–31. 22065615 10.1002/pmic.201100464

[155] Martens L, Chambers M, Sturm M, Kessner D, Levander F, et al (2011) mzML - A community standard for mass spectrometry data. Molecular and Cellular Proteomics 10 1: R110.000133. 10.1074/mcp.R110.000133 PMC3013463 20716697

[156] Chambers MC, MacLean B, Burke R, Amodei D, Ruderman DL, et al (2012) A cross-platform toolkit for mass spectrometry and proteomics. Nat Biotechnol 30: 918–920. 23051804 10.1038/nbt.2377 PMC3471674

[157] Specht M, Kuhlgert S, Fufezan C, Hippler M (2011) Proteomics to go: Proteomatic enables the user-friendly creation of versatile MS/MS data evaluation workflows. Bioinformatics 27: 1183–1184. 21325302 10.1093/bioinformatics/btr081

[158] Bald T, Barth J, Niehues A, Specht M, Hippler M, et al (2012) PymzML-python module for high-throughput bioinformatics on mass spectrometry data. Bioinformatics 28: 1052–1053. 22302572 10.1093/bioinformatics/bts066

[159] Geer LY, Markey SP, Kowalak JA, Wagner L, Xu M, et al (2004) Open mass spectrometry search algorithm. J Proteome Research 3: 958–964. 15473683 10.1021/pr0499491

[160] Craig R, Beavis RC (2004) TANDEM: Matching proteins with tandem mass spectra. Bioinformatics 20: 1466–1467. 14976030 10.1093/bioinformatics/bth092

[161] Specht M, Stanke M, Terashima M, Naumann-Busch B, Janßen I, et al (2011) Concerted action of the new Genomic Peptide Finder and AUGUSTUS allows for automated proteogenomic annotation of the *Chlamydomonas reinhardtii* genome. Proteomics 11: 1814–1823. 21432999 10.1002/pmic.201000621 PMC3145493

[162] Käll L, Storey JD, Noble WS (2009) Qvality: Non-parametric estimation of q-values and posterior error probabilities. Bioinformatics 25: 964–966. 19193729 10.1093/bioinformatics/btp021 PMC2660870

[163] Brock NL, Huss K, Tudzynski B, Dickschat JS (2013) Genetic dissection of sesquiterpene biosynthesis by *Fusarium fujikuroi* . Chembiochem 14: 311–315. 23335243 10.1002/cbic.201200695

