# Supplementary figures and images for: Deciphering the Cryptic Genome: Genome-wide Analyses of the Rice Pathogen Fusarium fujikuroi Reveal Complex Regulation of Secondary Metabolism and Novel Metabolites
Source: PLoS Pathog. 2013 Jun 27;9(6):e1003475. doi: 10.1371/journal.ppat.1003475 (PMC3694855; doi:10.1371/journal.ppat.1003475)

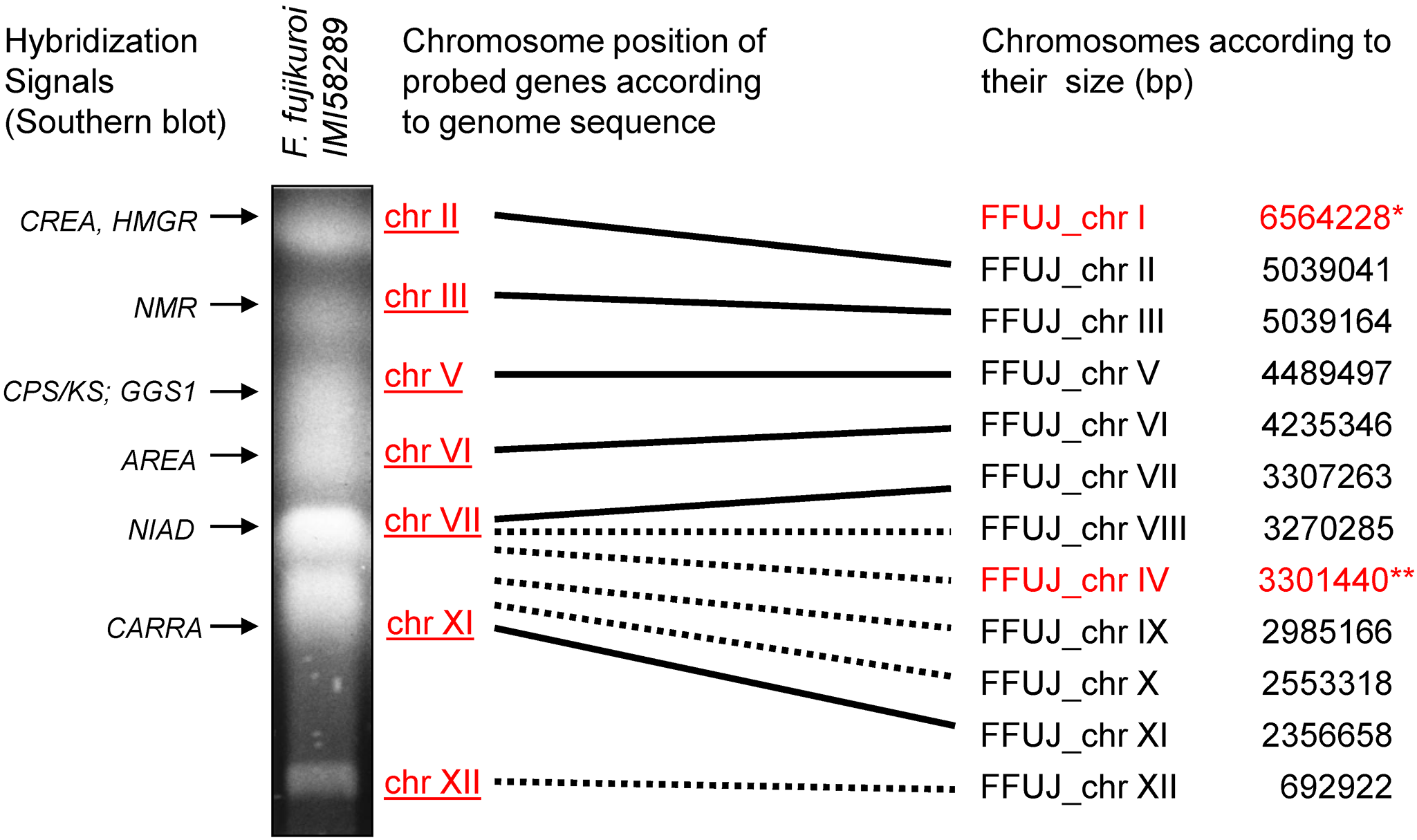

Supplement: Figure S1 — CHEF gel analysis of F. fujikuroi IMI58289. Previously we performed Southern blot analyses [39] of the CHEF gel with the following gene probes: CREA (FFUJ_04790), hmgR (FFUJ_04000), NMR (FFUJ_02636), GGS1(FFUJ_07352), CPS/KS (FFUJ_1433), AREA (FFUJ_06143), NIAD (FFUJ_12277), CARRA (FFUJ_11802). Arrows show the position of the hybridization signals, while the red letters for chromosome numbers show the location of these genes on defined chromosomes according to the genome sequence. Chromosome numbers on the right postulate the order of chromosomes I–XII according to the estimated chromosome sizes (kb). * Chromosome I is not shown: it was not separated under the used conditions and stayed in the slots of the gel. ** Chomosome IV is numbered according to the orthologous chromosome IV in F. verticillioides. However, this chromosome is significantly smaller in F. fujikuroi. Black lines indicate chromosomes that where confirmed by Southern blot hybridization. Dashed lines show postulated positions of the remaining chromosomes according to their size. (TIFF) [file ppat.1003475.s001.tiff]

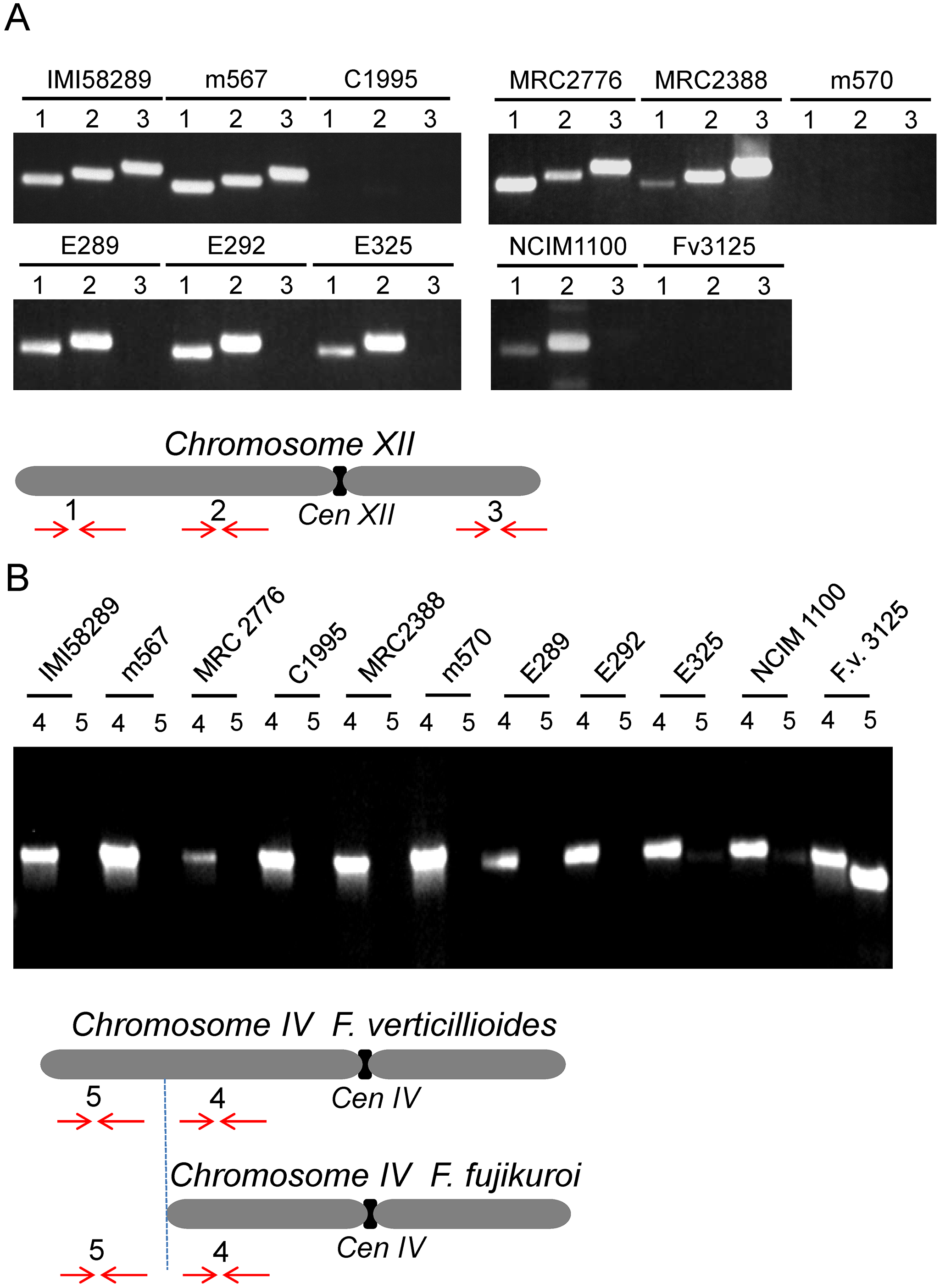

Supplement: Figure S2 — Variability of chromosomes XII and IV. A: PCR analysis reveals that chromosome XII is not present in all F. fujikuroi strains. 1, 2 and 3: PCR products derived from primer pairs designed from the sequence of genes FFUJ_14099, FFUJ_ 14193, and FFUJ_ 14245, respectively. The genes FFUJ_14099, FFUJ_14193, and FFUJ_14245 are located 0.7, 0.4 and 0.08 Mb from the left telomere of chromosome XII. The positions of these genes on chromosome XII are shown in the scheme below. B: Chromosome IV is shorter than in F. verticillioides. Primers were designed from the sequence of gene FVEG_11841 (4) present on chromosome IV in both F. fujikuroi and F. verticillioides (PCR bands were amplified in all strains tested) and FVEG_12503 (5), missing in F. fujikuroi but present in F. verticillioides (PCR bands amplified only in F. verticillioides, right lanes). The homolog of FVEG_11841 in F. fujikuroi, FFUJ_14790, is located 3.1 Mb from the left telomer. For FVEG_12503 no homolog is present in the F. fujikuroi genome. The positions of these genes on chromosome IV are shown in the scheme below. (TIFF) [file ppat.1003475.s002.tiff]

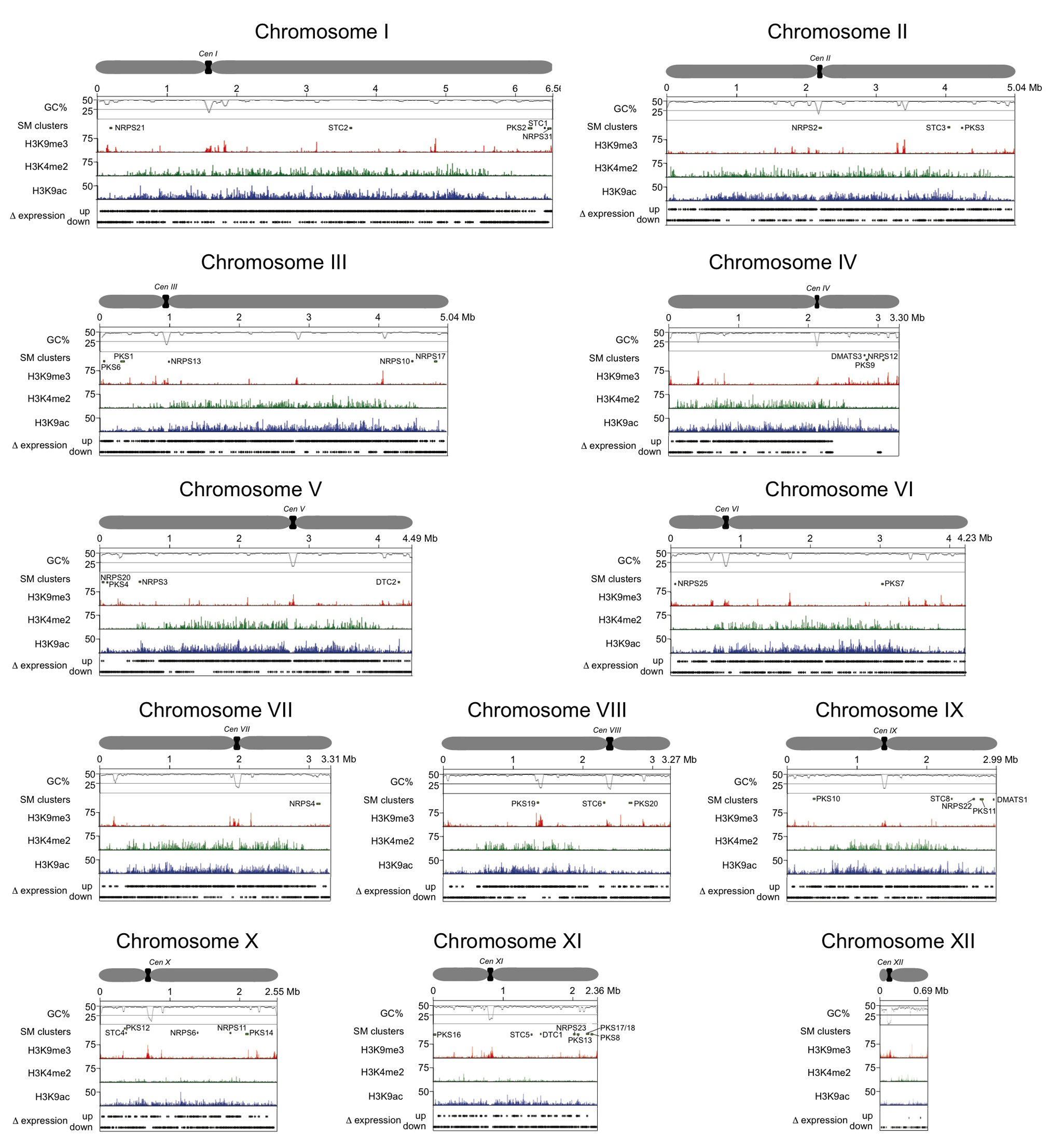

Supplement: Figure S3 — Characterization of F. fujikuroi chromosomes I–XII: variation in GC-content, acetylation and methylation statues of histone H3 and expression under low nitrogen conditions. For each chromosome, a diagram showing the position of the centromere is shown at the top; below this in descending order are: GC content, location of SM biosynthetic gene clusters, acetylation and methylation states of histone H3 protein, and changes in gene expression. Variation in histone H3 modification status serves as marker for chromosomal regions in which genes are expressed (H3K9ac and H3K4me2) or silent (H3K9me3). “Δ expression up” indicates a more than twofold increase in gene expression during growth of F. fujikuroi in a nitrogen-rich medium, whereas “Δ expression down” indicates an at least twofold decrease in gene expression. SM biosynthetic gene cluster locations are indicated by arrows labeled with the PKS, NRPS or TC (DTC means diterpene cyclase; STC means sesquiterpene cyclase) gene in each cluster (see Figure 3, Table 4 and Table S4). F. fujikuroi genes (FFUJ locus ID) to the left and right of the predicted centromere on each chromosome was identified and compared to the location of predicted orthologs in F. verticillioides (FVEG locus ID). “+” and “−”. (TIF) [file ppat.1003475.s003.tif]

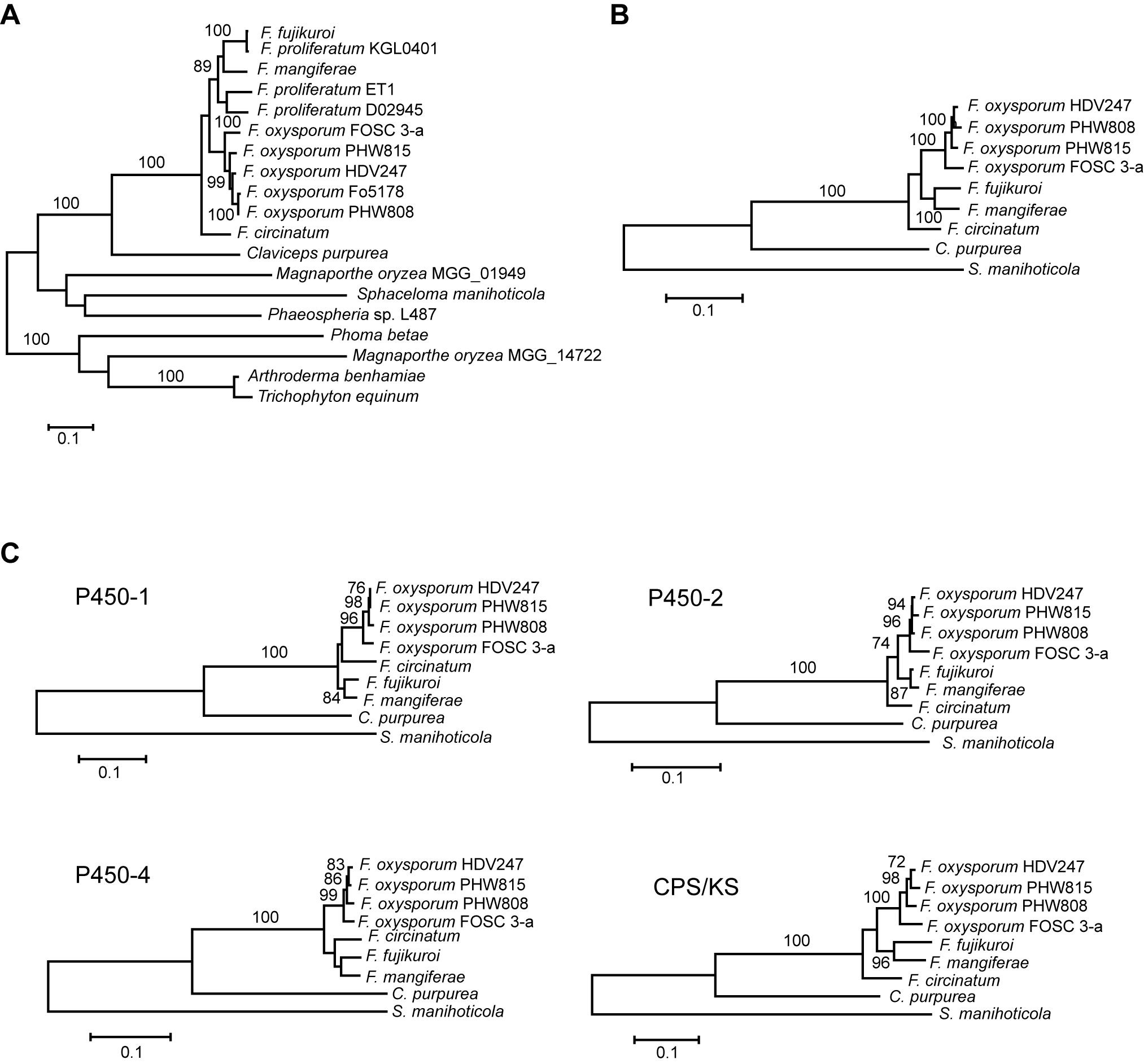

Supplement: Figure S4 — Maximum likelihood trees of selected GA biosynthetic genes. A: tree generated from Cps/Ks and related diterpene synthases from multiple genera of fungi; B: tree generated from concatenated sequences of GA enzymes/genes common to Fusarium species, Claviceps purpurea and Sphaceloma manihoticola. C: trees of four GA biosynthetic enzymes/genes (P450-1, P450-2, P450-4 and Cps/Ks) that are common to Fusarium, Claviceps and Sphaceloma; All trees were inferred from alignments generated by Muscle of deduced amino acid sequences, and all gaps in the alignment were removed prior to maximum likelihood analysis. Numbers near branches are bootstrap values based on 500 pseudoreplicates. Only bootstrap values >70 are shown. Muscle and maximum likelihood analyses were conducted using MEGA version 5 [163]. Sequences for F. proliferatum, and non-Fusarium genera other than Claviceps were obtained from the NCBI database via BLASTp analysis with F. fujikuroi homologues: NCBI accessions for terpene synthases for which accession or strain numbers are not shown in Figure S4A: A. benhamiae XP_003013365, Phaeospheria sp. L487, O13284; P. betae, BAD29971; S. manihoticola, CAP07655; T. equinum, EGE08989. The Claviceps sequence was supplied by Prof. Paul Tudzynski, University of Münster (unpublished data). F. oxysporum sequences were obtained from NCBI (strain Fo5176) or the Broad Institute's Fusarium Comparative Database. (TIF) [file ppat.1003475.s004.tif]

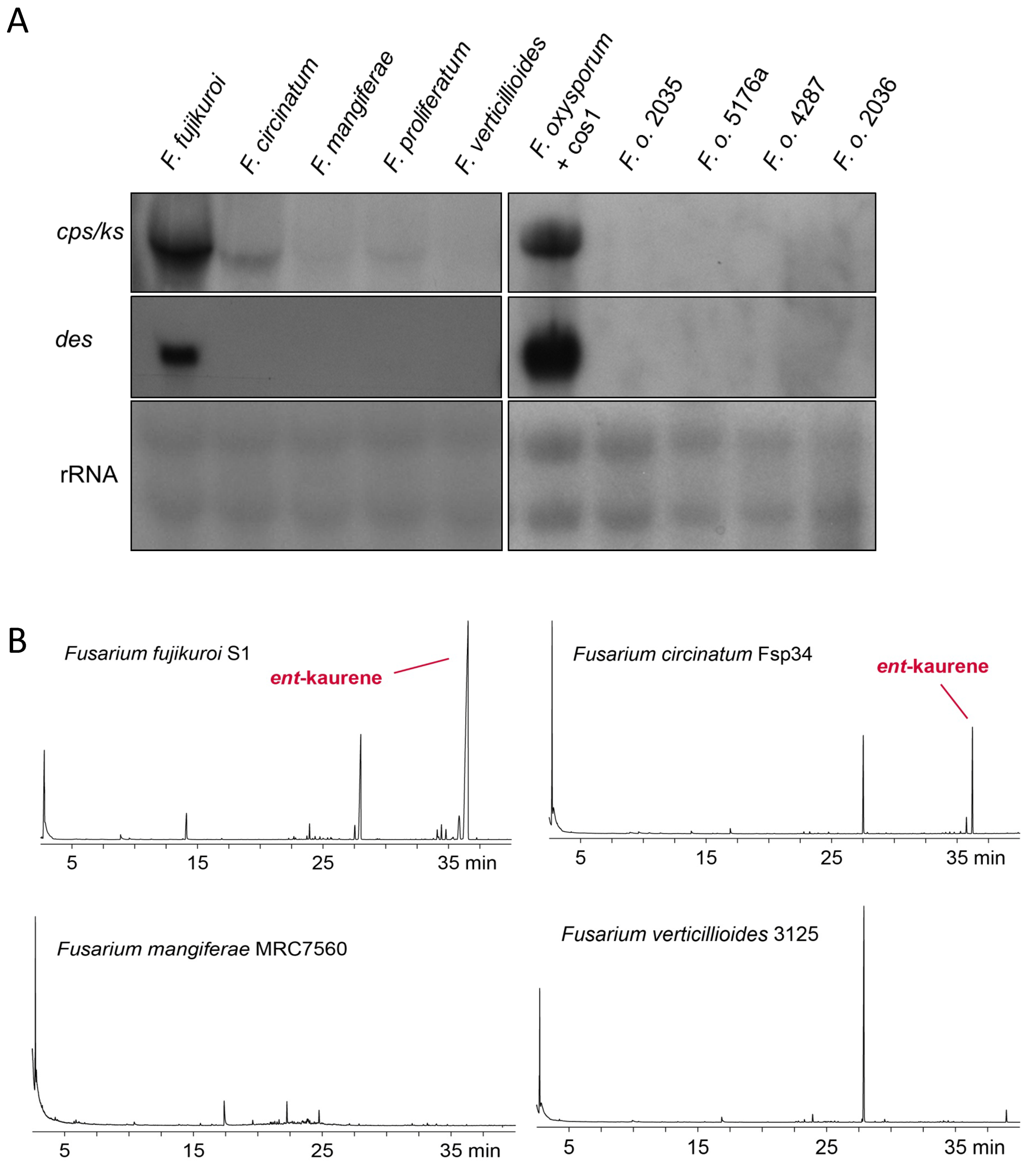

Supplement: Figure S5 — Gibberellin biosynthesis in species of the GFC. A: Expression of gibberellins biosynthetic genes cps/ks and des after three days of cultivation under nitrogen-limiting conditions (6 mM glutamine). All strains except for F. verticillioides contain the entire GA gene cluster. B: GC-MS analysis of ent-kaurene, the first specific intermediate of the GA pathway. (TIF) [file ppat.1003475.s005.tif]

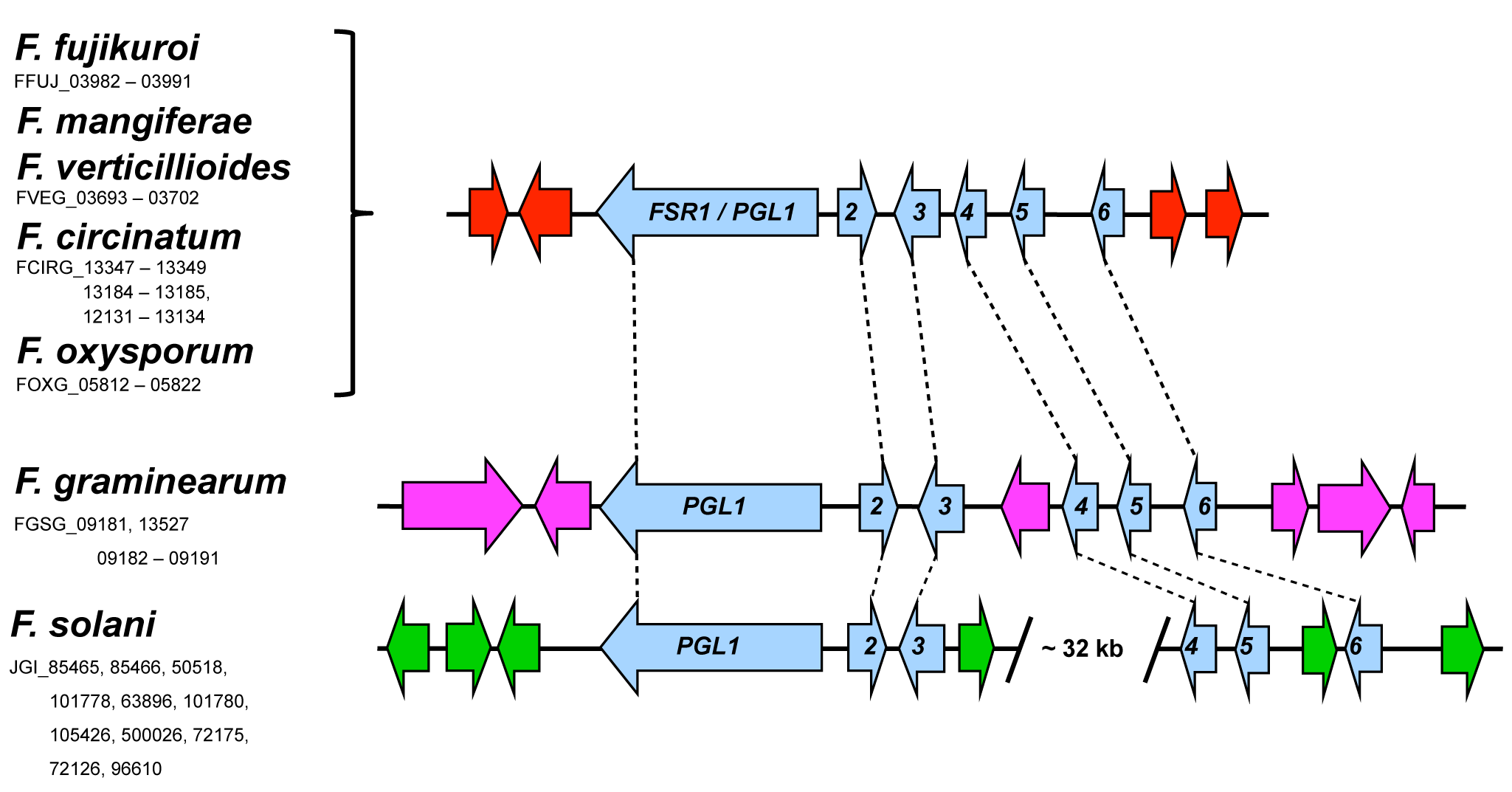

Supplement: Figure S7 — Comparison of the fusarubin biosynthetic gene ( FSR/PGL ) cluster in genomes sequences of Fusarium . Horizontal arrows that are the same color represent genes, or gene sets, that have closely related homologues in two or more species. Blue arrows represent FSR/PGL genes, and the numbers within these arrows correspond to FSR/PGL gene numbers rather than gene/protein model designations from genome databases. For those that are available, gene designations are indicated below species names. In F. solani, all FSR/PGL genes are not located within a contiguous cluster of genes. (TIF) [file ppat.1003475.s007.tif]

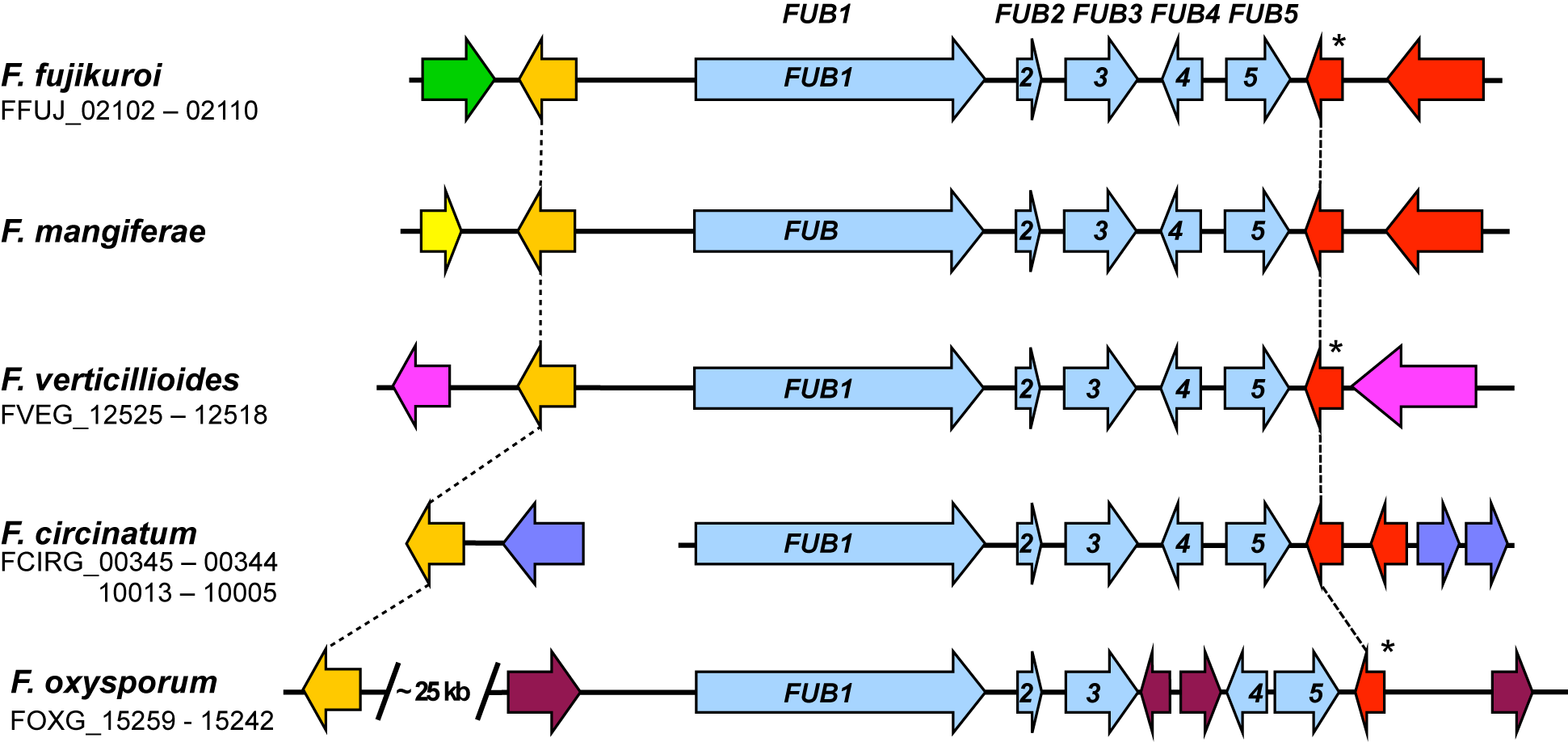

Supplement: Figure S8 — Comparison of the putative fusaric acid biosynthetic gene ( FUB ) cluster in genomes sequences of Fusarium . Horizontal arrows that are the same color represent genes, or gene sets, that have closely related homologues in two or more species. Blue arrows represent FUB genes, and the numbers within these arrows correspond to FUB gene numbers rather than gene/protein model designations from genome sequence databases. For those that are available, gene designations are indicated below species names. In F. oxysporum, the FUB cluster is interrupted by two genes. The asterisks mark genes without annotation. (TIF) [file ppat.1003475.s008.tif]

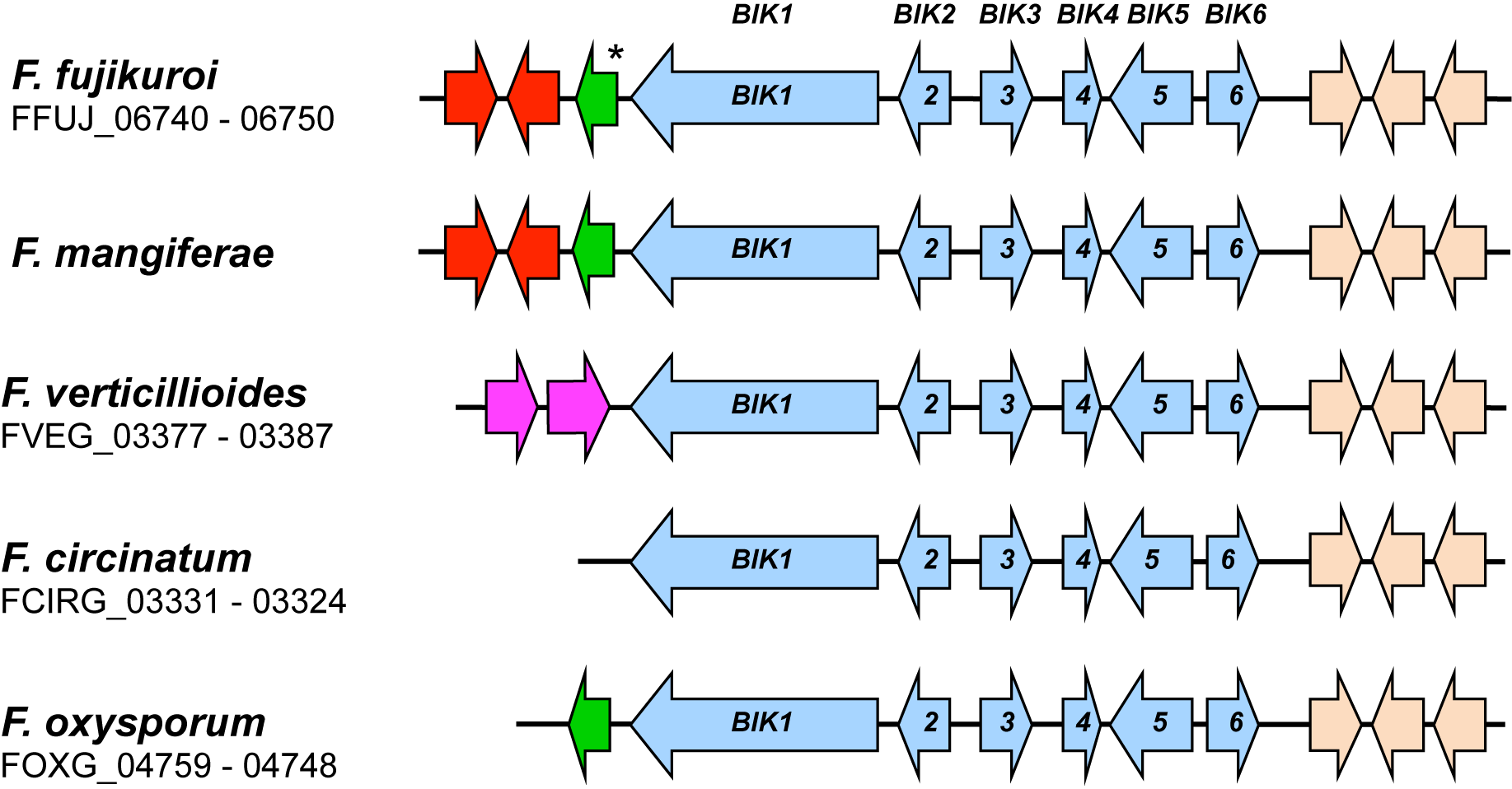

Supplement: Figure S9 — Comparison of the bikaverin biosynthetic ( BIK ) cluster in genomes sequences of Fusarium . BIK genes are represented by blue horizontal arrows, and the numbers within these arrows correspond to BIK gene numbers rather than gene/protein model designations from genome sequence databases. For those that are available, gene designations are indicated below species names. The asterisks marks FFUJ_14916. (TIF) [file ppat.1003475.s009.tif]

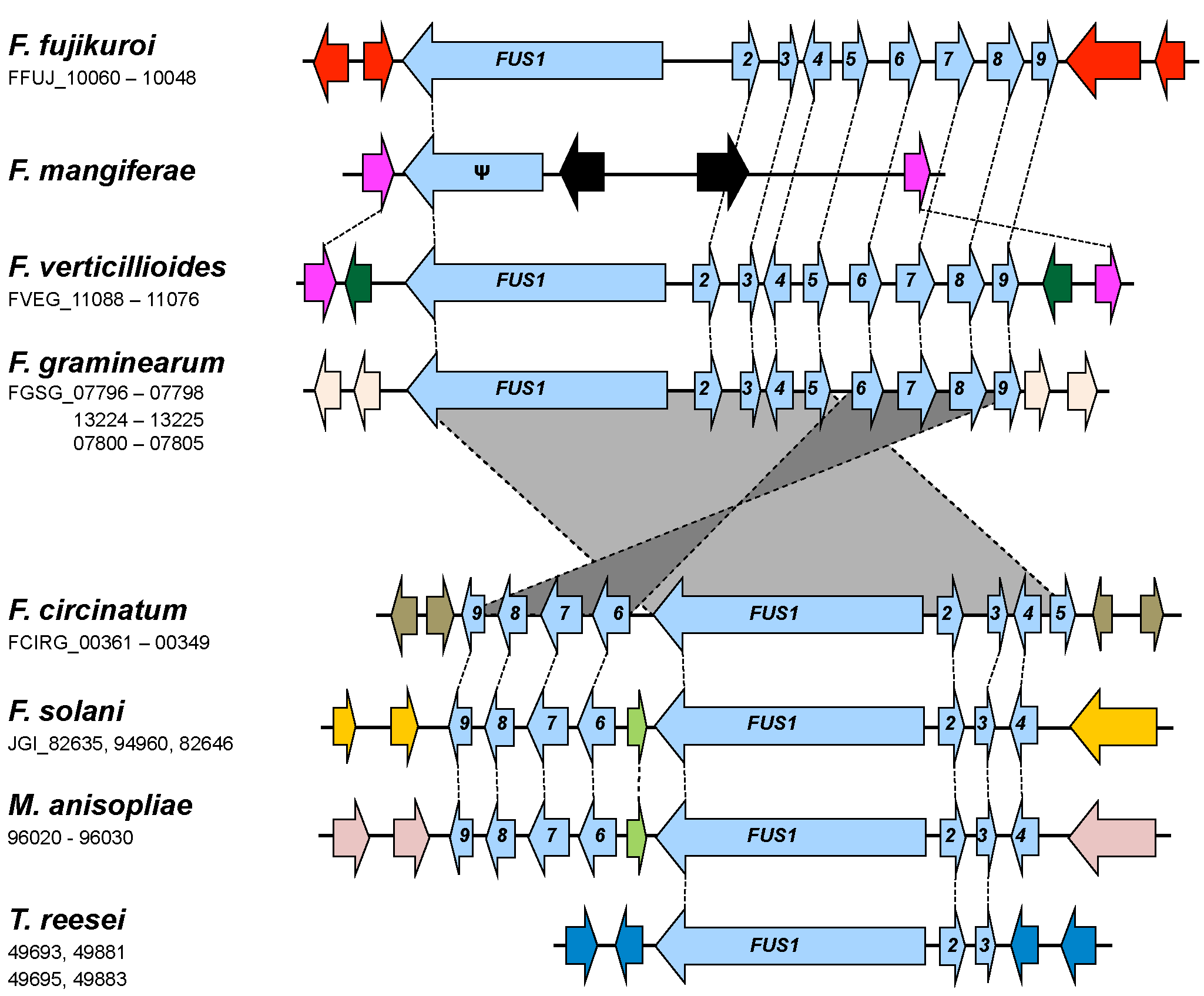

Supplement: Figure S10 — Comparison of the fusarin biosynthetic gene ( FUS ) cluster in genome sequences of Fusarium and the related fungus Metarhizium anisopliae as well as remnants of the FUS cluster in F. mangiferae and Trichoderma reesei. FUS genes are represented by blue horizontal arrows, and the numbers in the arrows correspond to FUS gene numbers. For those that are available, gene designations are indicated below species names. Ψ indicates a pseudogene. (TIF) [file ppat.1003475.s010.tif]

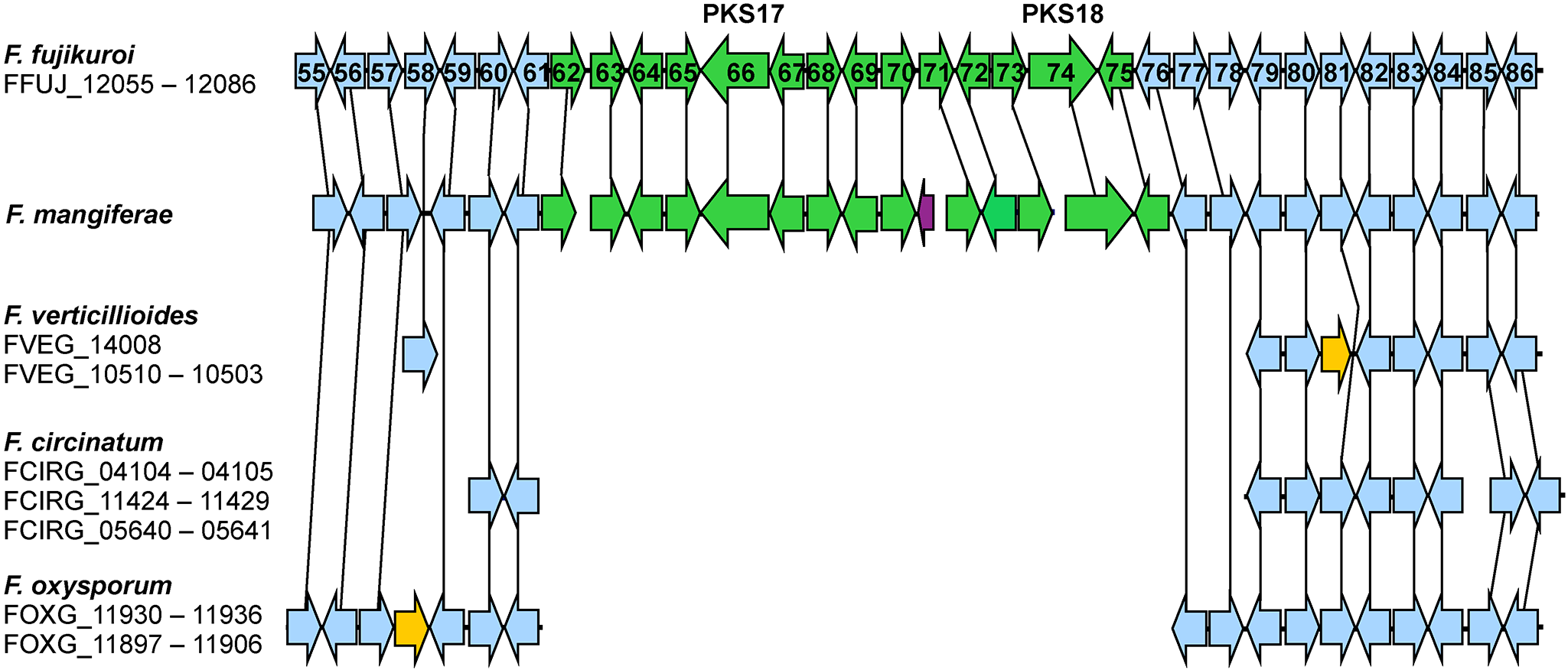

Supplement: Figure S11 — Comparison of the putative PKS17 – PKS18 gene cluster in the genome sequences of F. fujikuroi and F. mangiferae . The cluster genes are represented by green horizontal arrows, and cluster flanking genes are represented by blue or yellow arrows; yellow arrows represent genes that were not detected in the flanking regions of F. fujikuroi or F. mangiferae. Synteny of some cluster flanking regions is partially conserved in F. verticillioides, F. circinatum, and F. oxysporum, which lack the cluster. For those that are available, gene designations are indicated below species names. (TIF) [file ppat.1003475.s011.tif]

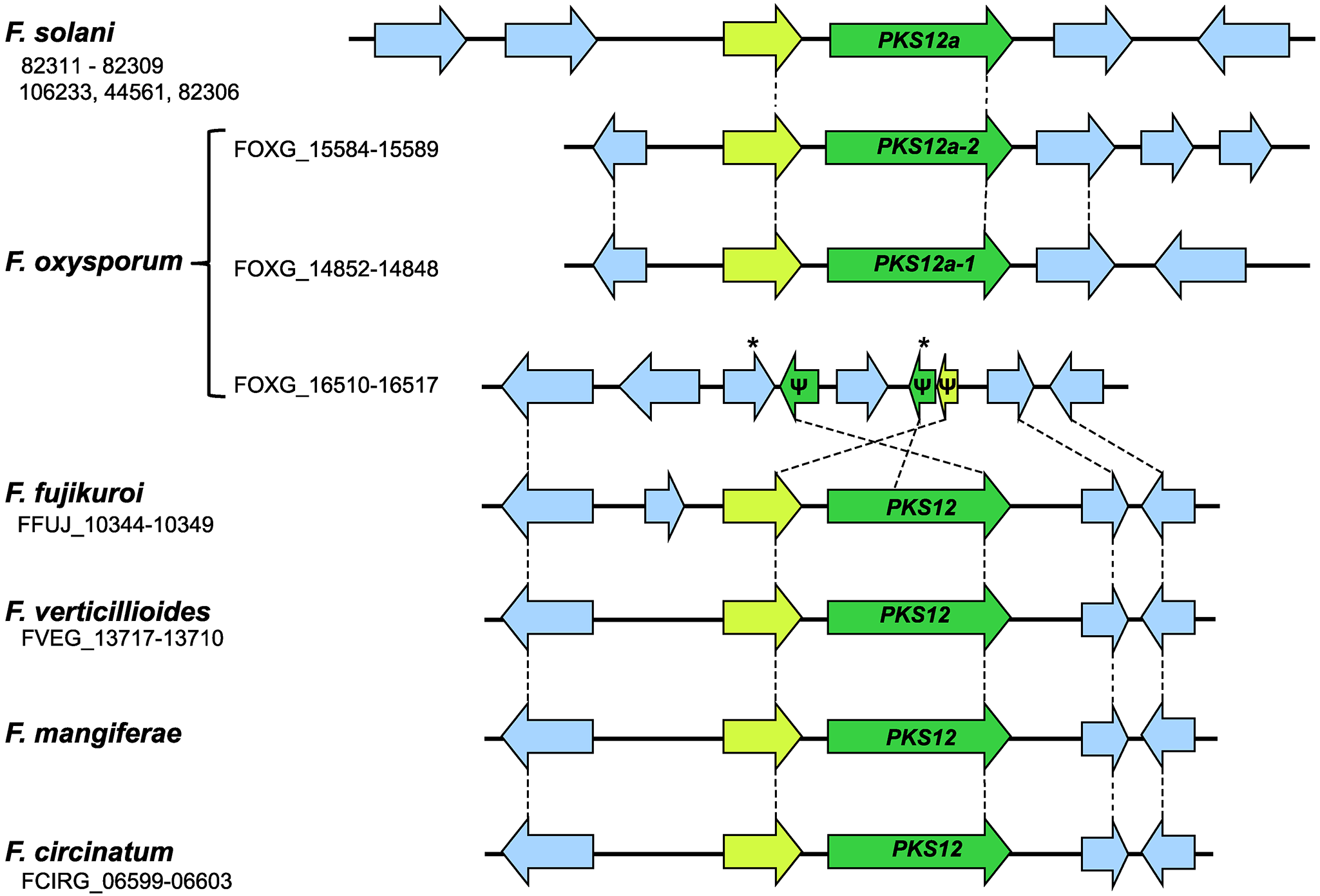

Supplement: Figure S12 — Comparison of the putative, two-gene PKS12 clusters (green arrows) in genome sequences of Fusarium . The second gene in the cluster is predicted to encode a methyltransferase. Blue arrows represent cluster flanking genes, which exhibit partial synteny conservation among the species examined. Based on phylogenetic analysis (Figure 5), PKS12 homologues have been arbitrarily designated as PKS12 and PKS12a until additional information becomes available. PKS12 is absent in the F. solani genome sequence, and only a remnant of it (indicated by Ψ) is present in the genome sequences of F. oxysporum. However, F. solani has a PKS12a homologue, and F. oxysporum strain Fol 4287 has two PKS12a paralogues (FOXG_14850 and FOXG_15586), which are part of a larger region of duplicated DNA that includes two putative methyltransferase genes, one on either side of each PKS12a paralogue. Other F. oxysporum genome sequences examined have only one PKS12a homologue. (TIF) [file ppat.1003475.s012.tif]

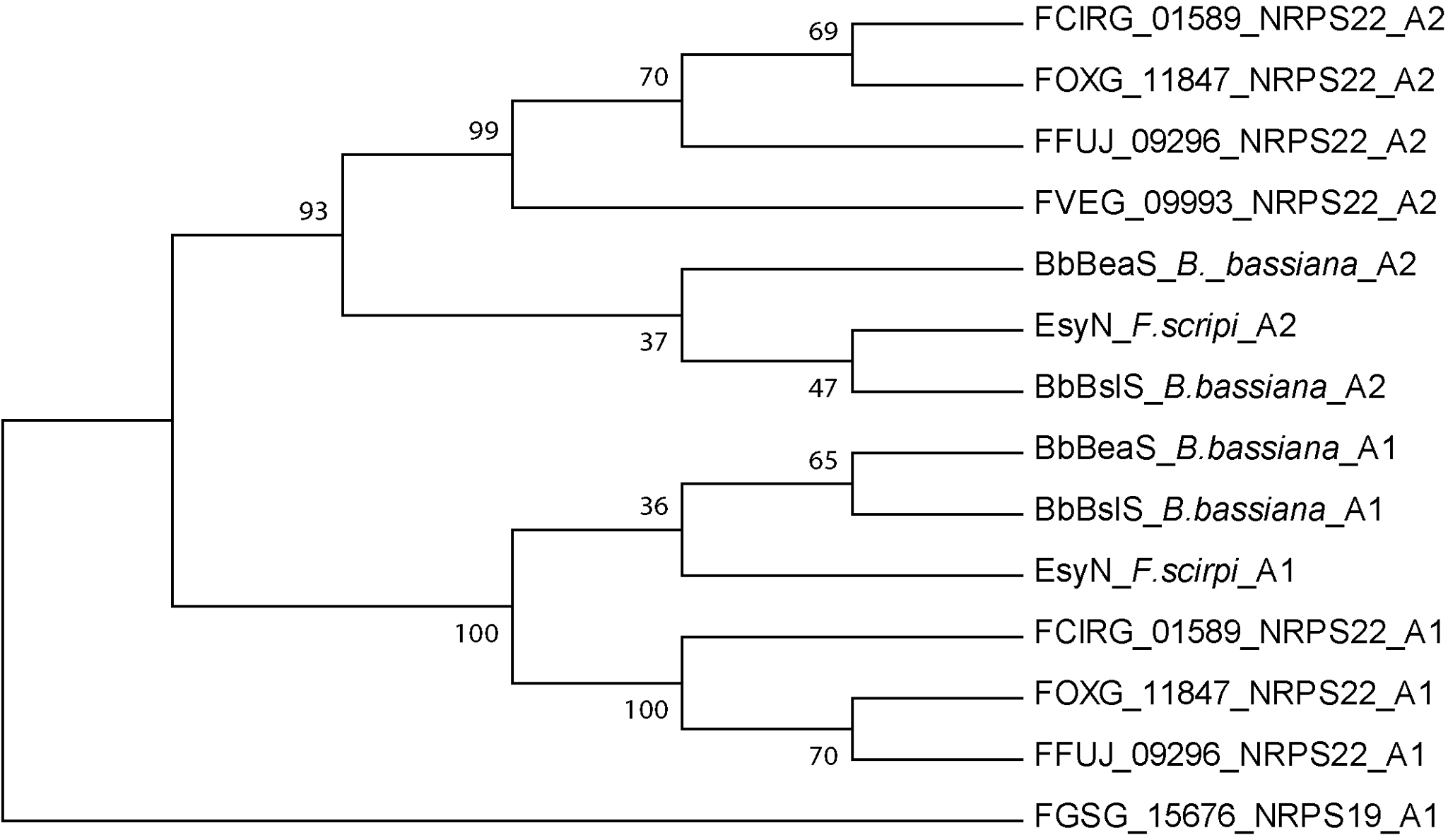

Supplement: Figure S13 — Phylogenetic tree of Fusarium NRPS22 and Beauveria bassiana BeaS and BslS as well as F. scirpi EsyN. The tree was generated by maximum parsimony analysis of alignements of deduced amino acid sequences of the A domains. The protein/gene designations FCIRG, FFUJ, FOXG and FVEG correspond to NRPSs deduced from genome sequences of F. circinatum, F. fujikuroi, F. oxysporum f. sp. lycopercisi strain Fol 4287, and F. verticillioides respectively. The tree is rooted with the A1 domain of the F. graminearum NRPS19. (TIF) [file ppat.1003475.s013.tif]

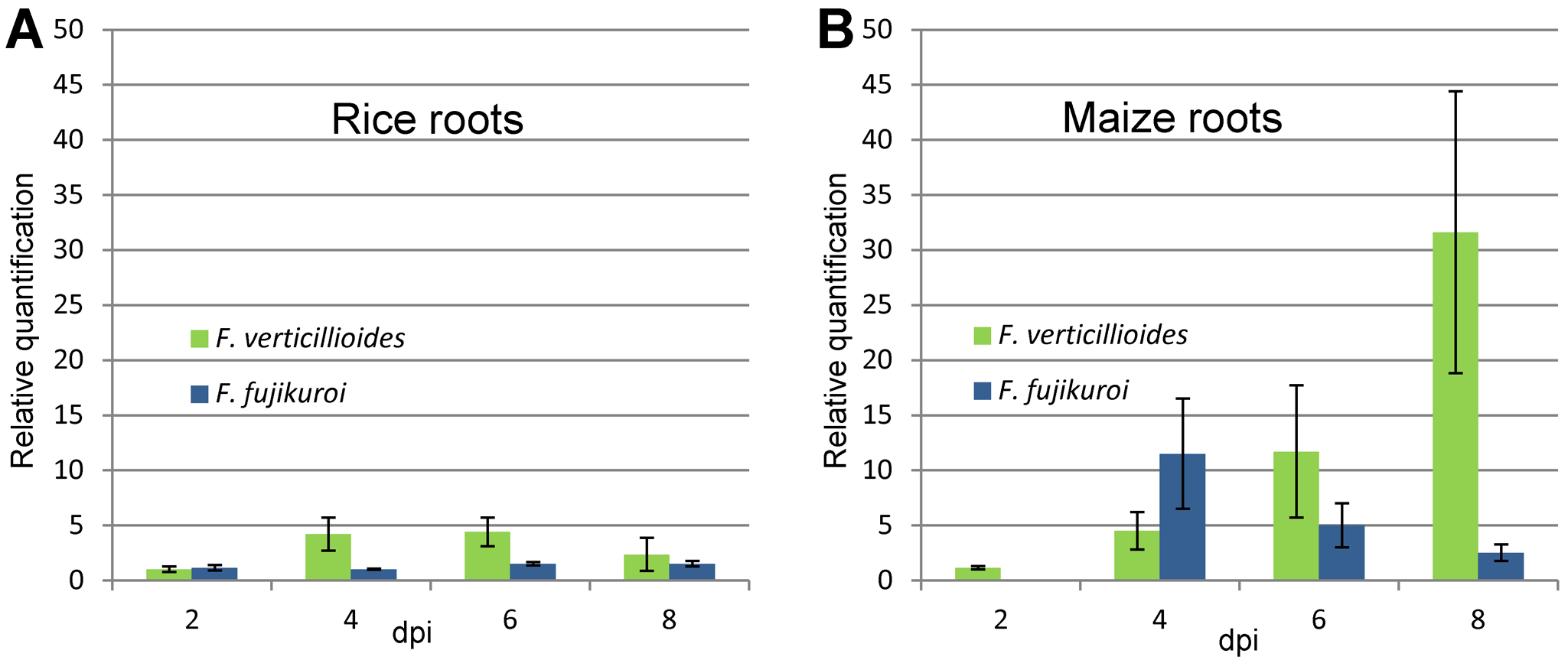

Supplement: Figure S14 — Relative expression of the fumonisin biosynthetic gene FUM1 from F. fujikuroi and F. verticillioides in rice and maize roots. Rice (A) and maize (B) roots were infected with Fusarium fujikuroi and F. verticillioides spores and every 2 days RNA was isolated from three or five plants and used in real time PCR analysis. The expression levels were obtained using the delta-delta Ct and were normalized against three reference genes encoding a related actin, the GDP-mannose transporter and ubiquitin. The expression levels of the F. verticillioides FUM1 at 2 days in rice was arbitrarily set as 1, and all other expression levels were reported relative to it. (TIF) [file ppat.1003475.s014.tif]

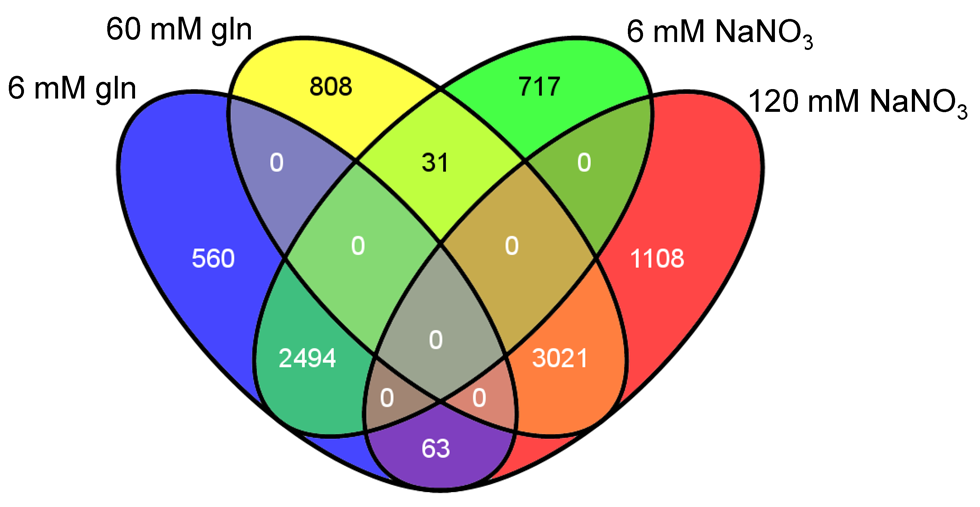

Supplement: Figure S15 — Venn diagram representing the distribution of nitrogen-and pH-regulated genes. Number of differentially regulated genes in wild-type F. fujikuroi cultivated under conditions that vary in nitrogen content and pH. The data were obtained by microarray analyses. The conditions were as follows: 6 mM gln (glutamine) constitutes, acidic low nitrogen; 60 mM gln, acid high nitrogen; 6 mM NaNO3 alkaline low nitrogen; and 120 mM NaNO3 alkaline high nitrogen. (TIF) [file ppat.1003475.s015.tif]

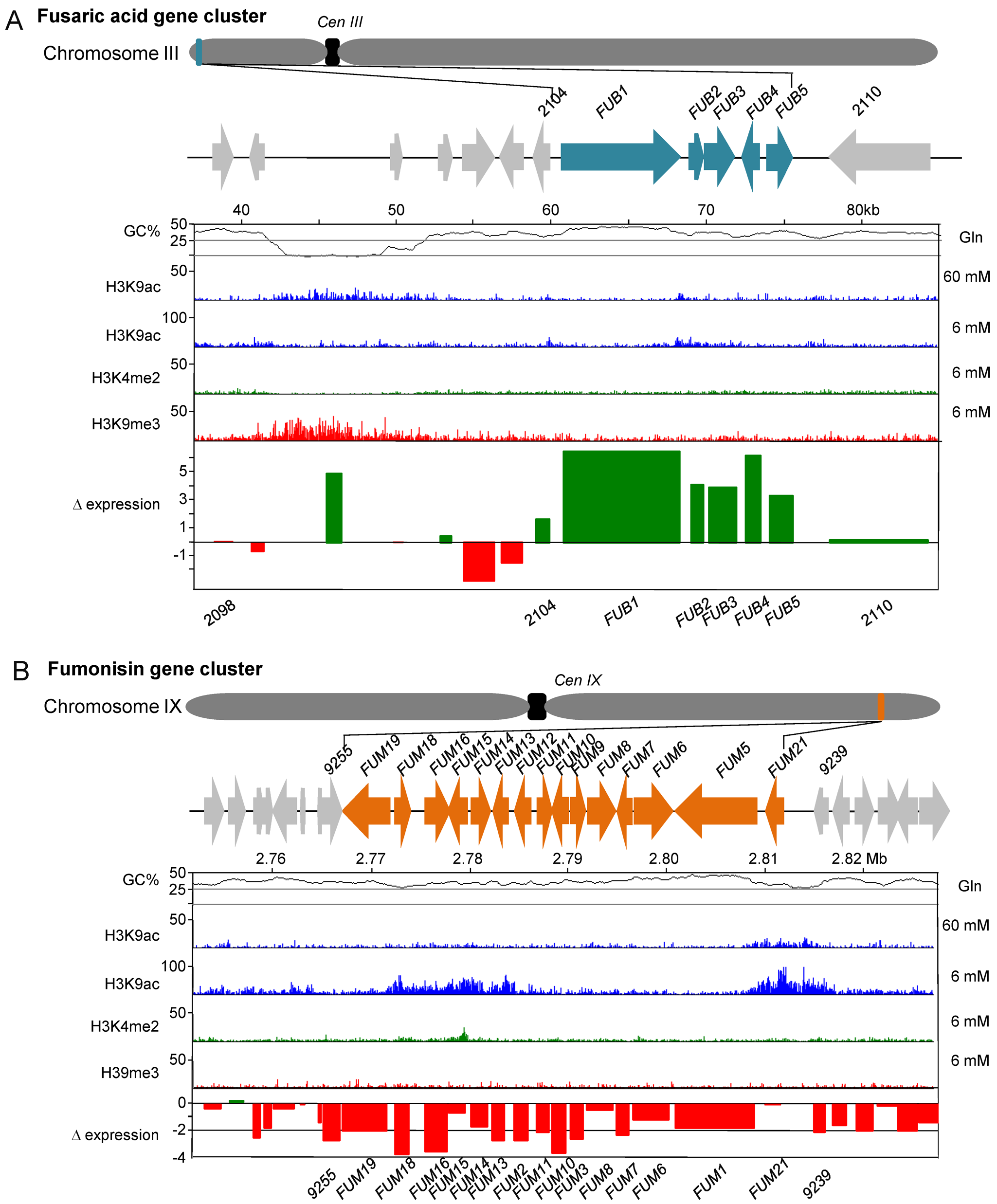

Supplement: Figure S16 — Expression pattern and distribution of active histone marks at the A: Fusaric acid (FUB) and B: at the fumonisin gene cluster. (TIF) [file ppat.1003475.s016.tif]
