# Supplementary material for: Deciphering the Cryptic Genome: Genome-wide Analyses of the Rice Pathogen Fusarium fujikuroi Reveal Complex Regulation of Secondary Metabolism and Novel Metabolites
Source: PLoS Pathog. 2013 Jun 27;9(6):e1003475. doi: 10.1371/journal.ppat.1003475 (PMC3694855; doi:10.1371/journal.ppat.1003475)
Supplement: Table S1 — Putative F. fujikuroi centromeric regions. Coordinates on chromosomes (Chr) I to XII include 0.5–1 kb of euchromatic regions on either side of pericentric and centromeric DNA and no attempt has been made to separate pericentric and centromeric regions. As none of the 14 scaffolds contain telomere repeats, all chromosome ends are unfinished. “Incompl R” indicates that the right arm of the chromosome does not have heterochromatin as indicated by presence of H3K9me3. (DOCX) [file ppat.1003475.s017.docx]

**Table S1: Putative *F. fujikuroi* centromeric regions.** Coordinates on chromosomes (Chr) I to XII include 0.5 - 1 kb of euchromatic regions on either side of pericentric and centromeric DNA and no attempt has been made to separate pericentric and centromeric regions. As none of the 14 scaffolds contain telomere repeats, all chromosome ends are unfinished. “Incompl R” indicates that the right arm of the chromosome does not have heterochromatin as indicated by presence of H3K9me3.

**Chr Sc coordinates size H3K9me3 in *Cen* Chr. compl?**

I 2 1,571,000 – 1,644,000 73 kb enriched on edges most

II 3 2,163,000 – 2,216,000 53 kb left, less right, edge most

III 4 927,000 – 994,000 67 kb enriched on edges most

IV 5 2,101,000 – 2,143,000 42 kb enriched on edges incompl R+L

V 7 2,721,000 – 2,796,000 75 kb enriched on edges most

VI 8 763,000 – 830,000 67 kb left, less right, edge incompl L

VII 9 1,922,000 – 2,004,000 82 kb enriched on edges incompl R

VIII 10 2,343,000 – 2,418,000 75 kb left, less right, edge most

IX 11 1,358,000 – 1,418,000 60 kb right, less left, edge incompl R+L

X 12 671,000 – 742,000 71 kb left, less right, edge most

XI 13 788,000 – 861,000 73 kb enriched on edges incompl R

XII 14 96,000 – 159,000 63 kb right, less left, edge most
