# Supplementary material for: Deciphering the Cryptic Genome: Genome-wide Analyses of the Rice Pathogen Fusarium fujikuroi Reveal Complex Regulation of Secondary Metabolism and Novel Metabolites
Source: PLoS Pathog. 2013 Jun 27;9(6):e1003475. doi: 10.1371/journal.ppat.1003475 (PMC3694855; doi:10.1371/journal.ppat.1003475)
Supplement: Table S2 — Nearest genes to F. fujikuroi centromeres and synteny with F. verticillioides . Nearest F. fujikuroi genes (FFUJ locus ID) to the left and right of the predicted centromere on each chromosome was identified and compared to the location of predicted orthologs in F. verticillioides (FVEG locus ID). “+” and “−” indicate strands, “t” indicates last gene on a specific F. verticillioides contig, “i”, indicates internal, non-terminal gene. (DOCX) [file ppat.1003475.s018.docx]

**Table S2: Nearest genes to *F. fujikuroi* centromeres and synteny with *F. verticillioides.*** Nearest *F. fujikuroi* genes (FFUJ locus ID) to the left and right of the predicted centromere on each chromosome was identified and compared to the location of predicted orthologs in *F. verticillioides* (FVEG locus ID). “+” and “-“ indicate strands, “t” indicates last gene on a specific *F. verticillioides* contig, “i”, indicates internal, non-terminal gene.

**Chromosome I *F. fujikuroi F. verticillioides***

Left FFUJ_01580 FVEG_09453 Chr1 sc13: 3126-4526 + t

Right FFUJ_01581 FVEG_01517 Chr1 sc1: 4623514-4624435 - t

**Chromosome II**

Left FFUJ_04619 FVEG_06492 Chr2 sc7: 2063525-2065367 + t

Right FFUJ_04618 FVEG_04300 Chr2 sc4: 2615607-2617367 + t

**Chromosome III**

Left FFUJ_02435 FVEG_12815 Chr3 sc20: 818852-821676 + t

Right FFUJ_02436 FVEG_07771 Chr3 sc10: 3905-6150 + t

**Chromosome IV**

Left FFUJ_13658 FVEG_05101 Chr4 sc5: 2325190-2325757 - t

Right FFUJ_13659 FVEG_12170 Chr4 sc18: 980476-982239 + t

FFUJ_13660

**Chromosome V**

Left FFUJ_07603 FVEG_02570 Chr5 sc3: 4082-5380 - t

Right FFUJ_07605 FVEG_08898 Chr5 sc12: 6325-8788 + t

**Chromosome VI**

Left FFUJ_05604 FVEG_13082 Chr6 sc22: 3068-4291 - t

Right FFUJ_05605 FVEG_02568 Chr6 sc2: 3217773-3220738 + t

(FFUJ_14424)

**Chromosome VII**

Left FFUJ_08498 FVEG_07159 Chr7 sc8: 1954239-1955687 + t

Right FFUJ_08497 FVEG_14171 unmap sc33: 15536-21104 +

**Chromosome VIII**

Left FFUJ_12597 FVEG_13839 Chr8 sc25: 4356-5228 + t

Right FFUJ_12598 FVEG_13323 Chr8 sc23: 6620-8733 - t

**Chromosome IX**

Left FFUJ_09746 FVEG_11377 Chr9 sc16: 1290010-1295391 - t

Right FFUJ_09745 FVEG_10477 Chr9 sc14: 1451438-1452410 - t

**Chromosome X**

Left FFUJ_10474 FVEG_13588 Chr10 sc24: 921-1397 + t

Right FFUJ_10475 FVEG_14116 unmap sc29: 89290-89654 - t

**Chromosome XI**

Left FFUJ_11521 FVEG_12816 Chr11 sc21: 4587-7142 + t

Right FFUJ_11522 FVEG_10963 Chr11 sc15: 1340243-1341211 + t

(FFUJ_14933)

**Chromosome XII (no homologous chromosome in *F. verticillioides*)**

Left FFUJ_14240 FVEG_13987 unmap sc26: 79-393 + t

Right FFUJ_14239 FVEG_12883 Chr11 sc21: 172434-172973 + i
