# Supplementary material for: Deciphering the Cryptic Genome: Genome-wide Analyses of the Rice Pathogen Fusarium fujikuroi Reveal Complex Regulation of Secondary Metabolism and Novel Metabolites
Source: PLoS Pathog. 2013 Jun 27;9(6):e1003475. doi: 10.1371/journal.ppat.1003475 (PMC3694855; doi:10.1371/journal.ppat.1003475)
Supplement: Table S5 — Gibberellin production by Fusarium spp. A: Gibberellin production by Fusarium species of the GFC and F. oxysporum 4287 (outgroup). F. oxysporum 4287 does not contain a GA gene cluster, but produces GAs after transforming it with the entire GA gene cluster from F. fujikuroi (cos1). B: Gibberellin production by F. oxysporum isolates with an entire GA gene. (DOCX) [file ppat.1003475.s021.docx]

**Table: S5A Gibberellin production by Fusaria with available genome sequence**

|  |  | | GA_3_ | GA_4_ | GA_7_ |
| --- | --- | --- | --- | --- | --- |
| ***F*. *fujikuroi* IMI58289** | -N | 6 mM glutamine | **+++** | **++** | **++** |
|  |  | 6 mM NaNO_3_ | **++** | **++** | **++** |
|  | +N | 60 mM glutamine | **-** | **-** | **-** |
|  |  | 120 mM NaNO_3_ | **+** | **+** | **-** |
|  |  | entire gene cluster | yes | | |
| ***F*. *circinatum* Fsp34** | -N | 6 mM glutamine | **-** | **-** | **-** |
|  |  | 6 mM NaNO_3_ | **-** | **-** | **-** |
|  | +N | 60 mM glutamine | **-** | **-** | **-** |
|  |  | 120 mM NaNO_3_ | **-** | **-** | **-** |
|  |  | entire gene cluster | yes | | |
| ***F*. *mangiferae* MRC7560** | -N | 6 mM glutamine | **-** | **-** | **-** |
|  |  | 6 mM NaNO_3_ | **-** | **-** | **-** |
|  | +N | 60 mM glutamine | **-** | **-** | **-** |
|  |  | 120 mM NaNO_3_ | **-** | **-** | **-** |
|  |  | entire gene cluster | yes | | |
| ***F*. *verticillioides* 3125** | -N | 6 mM glutamine | **-** | **-** | **-** |
|  |  | 6 mM NaNO_3_ | **-** | **-** | **-** |
|  | +N | 60 mM glutamine | **-** | **-** | **-** |
|  |  | 120 mM NaNO_3_ | **-** | **-** | **-** |
|  |  | entire gene cluster | no | | |
| ***F*. *oxysporum* 4287** | -N | 6 mM glutamine | **-** | **-** | **-** |
|  |  | 6 mM NaNO_3_ | **-** | **-** | **-** |
|  | +N | 60 mM glutamine | **-** | **-** | **-** |
|  |  | 120 mM NaNO_3_ | **-** | **-** | **-** |
|  |  | gene cluster | no | | |

- not detectable

+ peak intensity up to 10^5^ ++ peak intensity from 10^5^ to 10^6^ **?**

no sufficient genome data

**Table S5B Gibberellin production by *F. oxysporum* isolates with an entire GA gene cluster**

|  | | GA_3_ | GA_4_ | GA_7_ | **gene cluster** |
| --- | --- | --- | --- | --- | --- |
| ***F*. *oxysporum* 4287 +cos 1** | 6 mM NH_4_NO_3_ | +++ | ++ | +++ | **yes** |
| ***F*. *oxysporum* 4287** |  | **-** | **-** | **-** | **no** |
| ***F*. *oxysporum* 5176a** |  | **-** | **-** | **-** | **yes** |
| ***F*. *oxysporum* 2036** |  | **-** | **-** | **-** | **yes** |
| ***F*. *oxysporum* 2035** |  | **-** | **-** | **-** | **yes** |
| ***F*. *oxysporum* 2000** |  | **-** | **-** | **-** | **yes** |

++ peak intensity from 10^5^ to 10^6^ +++ peak intensity higher than 10^6^
