# Supplementary material for: Deciphering the Cryptic Genome: Genome-wide Analyses of the Rice Pathogen Fusarium fujikuroi Reveal Complex Regulation of Secondary Metabolism and Novel Metabolites
Source: PLoS Pathog. 2013 Jun 27;9(6):e1003475. doi: 10.1371/journal.ppat.1003475 (PMC3694855; doi:10.1371/journal.ppat.1003475)
Supplement: Table S6 — Secondary metabolite production in F. fujikuroi IMI58289, F. circinatum Fsp34, F. mangiferae MRC7560, F. verticillioides 3125 and F. oxysporum 4287. The strains were cultivated under four different culture conditions. Bikaverin, O-methylfusarubin, fusarins, fumonisin B1, fusaric acid and beauvericin were analyzed by HPLC-FTMS. (DOCX) [file ppat.1003475.s022.docx]

**Table S6**

|  |  | | **bikaverin** | ***O*-methyl-fusarubin** | **fusarins** | **fumonisin B1** | **fusaric acid** | **beauvericin**  **(ME)** |
| --- | --- | --- | --- | --- | --- | --- | --- | --- |
| ***F*. *fujikuroi* IMI58289** | -N | 6 mM glutamine | **+++** | **-** | **-** | **+** | **-** | **+** |
|  |  | 6 mM NaNO_3_ | **+** | **+++** | **-** | **-** | **++** | **+** |
|  | +N | 60 mM glutamine | **+** | **-** | **+++** | **-** | **++** | **+** |
|  |  | 120 mM NaNO_3_ | **+** | **-** | **++** | **-** | **+++** | **++** |
|  |  | entire gene cluster | **yes** | **yes** | **yes** | **yes** | **yes** | **yes** |
| ***F*. *circinatum* Fsp34** | -N | 6 mM glutamine | **++** | **-** | **-** | **-** | **-** | **++** |
|  |  | 6 mM NaNO_3_ | **++** | **+** | **-** | **-** | **+** | **+** |
|  | +N | 60 mM glutamine | **++** | **-** | **-** | **-** | **++** | **+++** |
|  |  | 120 mM NaNO_3_ | **++** | **-** | **-** | **-** | **++** | **+++** |
|  |  | entire gene cluster | **yes** | **yes** | **yes** | **no** | **yes** | **yes** |
| ***F*. *mangiferae* MRC7560** | -N | 6 mM glutamine | **+++** | **++** | **-** | **-** | **-** | **+++** |
|  |  | 6 mM NaNO_3_ | **+** | **++** | **-** | **-** | **-** | **+++** |
|  | +N | 60 mM glutamine | **++** | **-** | **-** | **-** | **+++** | **+++** |
|  |  | 120 mM NaNO_3_ | **+** | **-** | **-** | **-** | **+++** | **+++** |
|  |  | entire gene cluster | **yes** | **yes** | **(-)** | **no** | **yes** | **?** |
| ***F*. *verticillioides* 3125** | -N | 6 mM glutamine | **++** | **+** | **-** | **+++** | **-** | **-** |
|  |  | 6 mM NaNO_3_ | **+** | **+** | **-** | **-** | **+** | **-** |
|  | +N | 60 mM glutamine | **+** | **-** | **++** | **-** | **+++** | **-** |
|  |  | 120 mM NaNO_3_ | **-** | **-** | **-** | **-** | **++** | **-** |
|  |  | entire gene cluster | **yes** | **yes** | **yes** | **yes** | **yes** | **no** |
| ***F*. *oxysporum* 4287** | -N | 6 mM glutamine | **+++** | **-** | **-** | **-** | **-** | **+++** |
|  |  | 6 mM NaNO_3_ | **++** | **-** | **-** | **-** | **-** | **+++** |
|  | +N | 60 mM glutamine | **++** | **-** | **-** | **-** | **++** | **+++** |
|  |  | 120 mM NaNO_3_ | **+** | **-** | **-** | **-** | **+++** | **+++** |
|  |  | entire gene cluster | **yes** | **yes** | **no** | **no** | **yes** | **yes** |

- n.d. (-) cluster non functional

+ peak intensity up to 10^5^ ? no sufficient genome data

++ peak intensity up to 10^6^

+++ peak intensity higher than 10^6^

(ME) analyzed out of the extracted mycelium
