# Supplementary material for: Deciphering the Cryptic Genome: Genome-wide Analyses of the Rice Pathogen Fusarium fujikuroi Reveal Complex Regulation of Secondary Metabolism and Novel Metabolites
Source: PLoS Pathog. 2013 Jun 27;9(6):e1003475. doi: 10.1371/journal.ppat.1003475 (PMC3694855; doi:10.1371/journal.ppat.1003475)
Supplement: Table S8 — Primers used for all experiments. (DOCX) [file ppat.1003475.s024.docx]

**Tab. S8**-Material and Methods: Primers used in this study

| **Primer designation** | **Sequence** |
| --- | --- |
| POliC_seq_F1 | CCTTTCCCATCATCCATCTCCTC |
| DsRed_seq_R1 | GGCGAAGGGCAGGGGGCCGCCC |
| aps2-OE-for | TACCCCGCTTGAGCAGACATATGTCGCCACCAAGTCAAGATTG |
| aps2-OE-rev | GCGGATAACAATTTCACACAGGAAACAGCAGCCGAGATGAAGAAGGACAG |
| aps2-seq-rev | CTCCGAAGCTGAGGTATCAC |
| aps2-seq-rev2 | GTTCGAGGAGAGATCGAACTG |
| aps8-OE-for | TACCCCGCTTGAGCAGACATatgggactccacgatctccta |
| aps8-OE-rev | GCGGATAACAATTTCACACAGGAAACAGCcaaggataactggcagggatc |
| aps8-seq1-for | cgttgaaaggcaagtcctac |
| aps8-seq2-for | ggtatggcttacaacgctttg |
| aps8-seq-rev | ctgacttgcttgtggtggaac |
| gpd-yeast-for | agctctgtacagtgaccggtgac |
| gpd-yeast-rev | GCGGATAACAATTTCACACAGGAAACAGCAAGCTTgtgatgtctgctcaagcggggta |
| hph-OE-Prom | GTCACCGGTCACTGTACAGAGCTGACAGAAGATGATATTGAAGGAGC |
| hph-OE-Term | GTAACGCCAGGGTTTTCCCAGTCACGACGgatttcagtaacgttaagtggat |
| nat-OE-Prom | GTCACCGGTCACTGTACAGAGCTGCCGGATTGGTCAAGATTTGCGTCCG |
| nat-OE-Term | GTAACGCCAGGGTTTTCCCAGTCACGACGGACCAGGAGTTTCATAACATCCACGG |
| pks19TF-OE-for | TACCCCGCTTGAGCAGACATATGTCCCATCGGCAATCGTGCGA |
| pks19TF-OE-rev | GCGGATAACAATTTCACACAGGAAACAGCCTTCCAGCATGCTTCACTAAC |
| pks19-OE-for | TACCCCGCTTGAGCAGACATACAATGGGAAGCCAA |
| pks19-OE-rev | GCGGATAACAATTTCACACAGGAAACAGCGACACTCCACCG |
| pks19-TF-seq1 | GACCCTCAAACTCTAGTCG |
| pks19-TF-seq2 | CGACGAGGCTAGTTCCAATTC |
| pks19-seq1 | CGCCATCGACACAGCG |
| FF_12241-f1 | CCTCGTGGTATTGGTGCC |
| FF_12241-r1 | CCCATGCGCTCATAAGCC |
| FF_12242-f1 | GGTGTCTGCAGTGCCATTGG |
| FF_12242-r1 | CCACAAGGCACATAACGCC |
| FF_12243-f1 | CACATGAGAAGGCAAGGC |
| FF_12243-r1 | CCGTTGACTTGAGCC |
| FF_12244-f1 | GTCTACTTACTCCAGGC |
| FF_12244-r1 | GGTCTCCATGTTGAGG |
| FF_14099-f1 | CGGACCCAATGGTCTACTTG |
| FF_14099-r1 | GCGCAGATGCTTCAACCTC |
| FF_14193-f1 | CCCCAACAGAAGAACAGCTC |
| FF_14193-r1 | CTTGAGAAAAGTCGGCCATC |
| FF_14245-f1 | CTGCAGCTAAAGGGCTTCTG |
| FF_14245-r1 | CCATACAAGGCTGCAATGTG |
| FRACRTPCRFW | GAGAACGAGCGTGTCTTGATTGAGCC |
| FRACRTPCRRV | TTTCCTCCGCAGAATGAAGAAGGACTC |
| FGMTRTPCRFW | GAGGACTCGCGCAACCGTATCTTC |
| FGMTRTPCRRV | CTTGTTGAGAGCACCAACCATGGAGTA |
| FBUPRTPCRFW | CCAACCCTGACGATCCTCTTGTGC |
| FUBRTPCRRV | TACTTTCGAGTCCACTCCCGAGCTG |
| FFUM1RTPCRFW2 | GCCTCATGTGAGGAAGACCC |
| FFUM1RTPCRRV2 | GTTGGGTCCATCTCTTCGACAGG |
| FAPS1RTPCRFW | CTGCTTCACTGCTTGGACGCTTTCC |
| FAPS1RTPCRRV | CCAATGTGAGTTATGCCAATACTCCTGG |
| FPKS19RTPCRFW | GCTCTGGGTAACATGCTCGAGATCG |
| PKS19RTPCRRV | CATTCTCTAATCCGAATAGCCACAAGCG |
| FCPSKSRTPCRFW2 | GACGACGAGGCTGAAGATTACCTGAG |
| FCPSCSRTPCRRV2 | CGCGCAAGCCATCACCATCGATTTG |
