# Supplementary material for: Deciphering the Cryptic Genome: Genome-wide Analyses of the Rice Pathogen Fusarium fujikuroi Reveal Complex Regulation of Secondary Metabolism and Novel Metabolites
Source: PLoS Pathog. 2013 Jun 27;9(6):e1003475. doi: 10.1371/journal.ppat.1003475 (PMC3694855; doi:10.1371/journal.ppat.1003475)
Supplement: Table S9 — Overview of the metabolites that were analyzed by HPLC-FTMS. The analytes were identified by their retention time and isotope pattern compared to standard substances. For the estimation of the produced amount, the m/z of their most intense ions were observed. (DOCX) [file ppat.1003475.s025.docx]

**Table S9: Retention time and most intense MS-signal of analyzed metabolites**

| metabolite | retention time [min] | *m/z* [most intense adduct] |
| --- | --- | --- |
| gibberellic acid 3 | 16.4 | 369.1308 [M+Na]^+^ |
| gibberellic acid 4 | 34.7 | 355.1516 [M+Na]^+^ |
| gibberellic acid 7 | 33.5 | 353.1361 [M+Na]^+^ |
| bikaverin | 42.4 | 383.0762 [M+H]^+^ |
| *O*-methylfusarubin | 25.6 | 321.0969 [M+H]^+^ |
| fusarins | 36-38 | 454.1836 [M+Na]^+^ |
| fumonisin B_1_ | 30.2 | 722.3958 [M+H]^+^ |
| fusaric acid | 12.9 | 180.1019 [M+H]^+^ |
| beauvericin | 50.9 | 806.3987 [M+Na]^+^ |
