# Supplementary material for: Deciphering the Cryptic Genome: Genome-wide Analyses of the Rice Pathogen Fusarium fujikuroi Reveal Complex Regulation of Secondary Metabolism and Novel Metabolites
Source: PLoS Pathog. 2013 Jun 27;9(6):e1003475. doi: 10.1371/journal.ppat.1003475 (PMC3694855; doi:10.1371/journal.ppat.1003475)
Supplement: Table S10 — Overview of the analysed amino acids. The amino acids that are obtained after acid hydrolysis of apicidin and the apicidin metabolite were identified based on the accurate mass of their [M+H]+ ions. (DOCX) [file ppat.1003475.s026.docx]

| **amino acid** | ***m/z* [M+H]^+^** |
| --- | --- |
| isoleucin | 132.1019 |
| pipecolic acid | 130.0863 |
| 2-amino-8-oxodecanoic acid | 202.1438 |
| phenylalanine | 166.0863 |
| ‘new’ amino acid | 190.1074 |

**Table S10: Exact masses of [M+H]^+^ ions of analyzed amino acids**
